# Resistance gene–guided genome mining reveals the roseopurpurins as inhibitors of cyclin-dependent kinases

**DOI:** 10.1073/pnas.2310522120

**Published:** 2023-11-20

**Authors:** Kyle L. Dunbar, Bruno Perlatti, Nicholas Liu, Amber Cornelius, Daniel Mummau, Yi-Ming Chiang, Lawrence Hon, Monika Nimavat, Jason Pallas, Sina Kordes, Ho Leung Ng, Colin J. B. Harvey

**Affiliations:** ^a^Hexagon Bio, Menlo Park, CA 94025; ^b^Proteros Biostructures GmbH, Planegg D-82152, Germany

**Keywords:** genome mining, biosynthesis, natural products

## Abstract

With the significant increase in the availability of microbial genome sequences in recent years, resistance gene–guided genome mining has emerged as a powerful approach for identifying natural products with specific bioactivities. Here, we present the use of this approach to reveal the roseopurpurins as potent inhibitors of cyclin-dependent kinases (CDKs), a class of cell cycle regulators implicated in multiple cancers. We identified a biosynthetic gene cluster (BGC) with a putative resistance gene with homology to human CDK2. Using targeted gene disruption and transcription factor overexpression in *Aspergillus uvarum*, and heterologous expression of the BGC in *Aspergillus nidulans,* we demonstrated that roseopurpurin C (**1**) is produced by this cluster and characterized its biosynthesis. We determined the potency, specificity, and mechanism of action of **1** as well as multiple intermediates and shunt products produced from the BGC. We show that **1** inhibits human CDK2 with a *K*_iapp_ of 44 nM, demonstrates selectivity for clinically relevant members of the CDK family, and induces G1 cell cycle arrest in HCT116 cells. Structural analysis of **1** complexed with CDK2 revealed the molecular basis of ATP-competitive inhibition.

## Significance

Resistance gene–guided genome mining presents a solution to a longstanding challenge in natural product drug discovery by allowing the prediction of a natural product’s molecular target directly from the sequence of the gene cluster responsible for its biosynthesis. We apply the approach to reveal roseopurpurin C as a potent inhibitor of Cyclin-dependent kinase 2 (CDK2). This work represents an example of the use of resistance gene–guided genome mining in fungi for the identification of an inhibitor of a target that has been clinically validated for the treatment of multiple cancers.

Bioactive natural products have provided significant therapeutic benefits for many diseases; however, it has historically been challenging to establish the mechanism of action of these natural products and identify the target protein(s) with which they interact. With the rise in available genome sequences, resistance gene–guided genome mining has emerged as a powerful tool to identify natural products targeting a specific protein of interest. This approach relies on the insight that in some biosynthetic gene clusters (BGCs), the producing organism encodes within the BGC a resistance gene in the form of a copy of the molecular target of the BGC’s product (1). This resistance gene is not required for the biosynthesis of any natural products but serves to protect the host from the toxicity of its own metabolites. In fungi, this phenomenon was first appreciated with the identification of a copy of 3-hydroxy-3-methylglutaryl coenzyme A (HMG-CoA) reductase within the lovastatin BGC of *Aspergillus terreus* (2, 3). Multiple other resistance gene–encoding BGCs have since been identified in fungi (4), including BGCs that produce inhibitors of ergosterol biosynthesis (squalestatin (5), restricticin (6), and hymeglusin (7)), the proteasome (fellutamide B (8)), glycolysis (heptelidic acid (9)), oxidative phosphorylation (aurovertin and citreoviridin (10)), and calcineurin (cyclosporine A (11)). In each of these cases, the identification of a resistance gene validated the relationship between a known natural product and a molecular target that had been empirically determined previously, in some cases decades prior to the characterization of the BGC. More recently, resistance gene–guided genome mining has been used more prospectively to identify aspterric acid (12) as an inhibitor of branched-chain amino acid biosynthesis, a promising point of intervention for herbicides for use in agriculture. Here, we extend this approach to the identification of **1** as an inhibitor of CDK2 demonstrating the use of resistance gene–guided genome mining in fungi for the identification of an inhibitor of a clinically validated target in oncology.

Fungal natural products have been shown to be potent and specific inhibitors of a variety of human kinases (13, 14). With this in mind, we sought to use resistance gene–guided mining of fungal genomes to identify inhibitors of the cyclin-dependent kinases (CDKs). The CDKs are a family of kinases that are critical factors in both the control of the cell cycle and protein translation. In fungi, the CDK family comprises 6 proteins. In humans, this family has diverged to comprise 20 proteins (15). The misregulation of several CDK proteins in a variety of cancers makes them attractive drug targets (16).

**Fig. 1.**
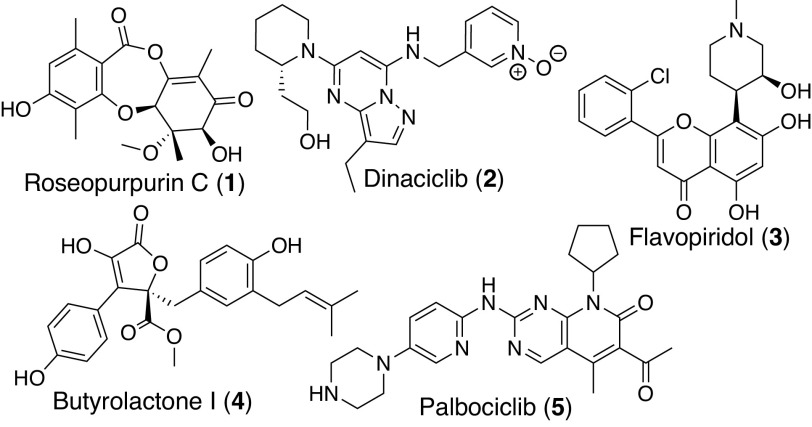
Characterized inhibitors of human CDKs.

Several inhibitors of the CDK protein family have been identified using both traditional screening and computational methods (17, 18). These include synthetic compounds dinaciclib (**2**, CDK2/5/9) (19) and palbociclib (**5**, CDK4/6) (20) along with flavopiridol (**3**, CDK1/2/4/7) (21) and butyrolactone I (**4**, CDK2) (22), two compounds of natural origin (Fig. 1). Here, we expand the list of CDK inhibitors by using resistance gene–guided genome mining to identify a family of BGCs present across multiple genomes that encode a resistance gene with homology to human CDK2. Through a combination of in situ cluster engineering in *Aspergillus uvarum* and heterologous expression in *Aspergillus nidulans*, we demonstrate that these BGCs produce roseopurpurin C (**1**), a depsidone natural product. We characterize the activity of **1,** revealing it as a potent ATP-competitive inhibitor of CDK2 (*K*_iapp_ of 44 nM) with selectivity for CDK family kinases which induces G1 phase cell cycle arrest. We profile several precursors and shunt products of **1** to define a preliminary structure–activity relationship, and, in combination with the co-crystal structure of **1** bound to human CDK2, we define the structural basis for CDK2 engagement.

## Results

### Identification and Characterization of the Roseopurpurin BGC.

**Fig. 2.**
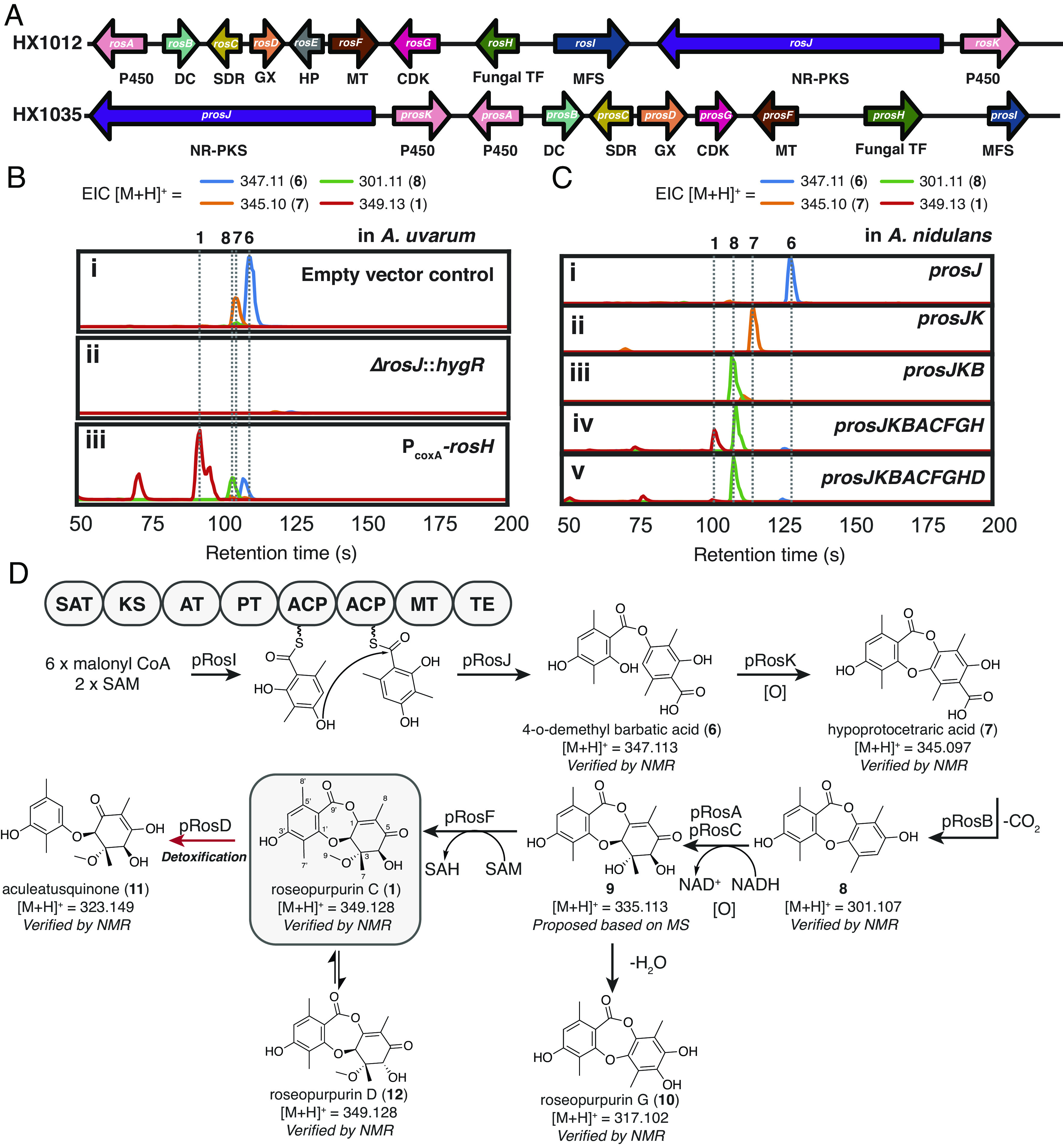
Biosynthesis of **1**. (*A*) Overview of BGCs HX1012 and HX1035. Abbreviations: P450 = cytochrome P450, DC = decarboxylase, SDR=short-chain dehydrogenase/reductase, MT= methyltransferase, Fungal TF = fungal transcription factor, MFS = major facilitator superfamily transporter, GX = glyoxalase, HP = hypothetical protein (*B*) Extracted ion chromatograms (EICs) of **1** and related compounds in cultures of *A. uvarum* i), core-gene disruption mutant *ΔrosJ::hygR* ii) and transcription factor overexpression strain P_coxA_-*rosH* iii). All cultures grown in GLX media. (*C*) Products of BGC HX1035 expressed in *A. nidulans*. (*D*) Proposed biosynthetic pathway for **1** and related intermediates and shunt and degradation products. Domain abbreviations: SAT = starter unit ACP transacylase, KS = ketosynthase, AT = acyltransferase, PT = Product template, ACP = acyl carrier protein, MT = methyltransferase, TE = thioesterase.

To find gene clusters capable of producing inhibitors of CDK2, we looked within our library of annotated BGCs for those that contained a gene encoding a protein that had ≥50% amino acid identity with human CDK2. This search yielded seven related BGCs from diverse fungal species, including BGC HX1012 in the genome of *A. uvarum* and BGC HX1035 in the genome of *Phaeosphaeria* sp. TTI001159 (Fig. 2*A* and *SI Appendix*, Fig. S1). The biosynthetic enzymes encoded in these BGCs comprise one nonreducing-polyketide synthase (NR-PKS) core protein, multiple putative tailoring enzymes, a cluster-specific transcription factor, a major facilitator superfamily (MFS) transporter, and a CDK2 homolog. Metabolites are produced by BGCs through the combined activity of the enzymes encoded by the core genes and most or all of the tailoring genes. The consecutive nature of the transformations in these pathways leads to the production of multiple metabolites, as both intermediates and shunt products, at varying levels of maturity from a single BGC. From a therapeutic discovery perspective, it is valuable to profile the bioactivity of metabolites at all stages of maturation, as each metabolite provides potentially valuable data information about the structure–activity relationship of the compound class.

To identify the metabolites produced by BGC HX1012, we profiled the metabolome of *A. uvarum*. However, in addition to BGC HX1012, the genome of *A. uvarum* encodes 75 additional BGCs (per antiSMASsh 6.0 (23)), which can each produce unique metabolites. Given the abundance of metabolites, it can be challenging to determine which are produced by a specific BGC. To determine the metabolites specific to BGC HX1012, we employed targeted gene disruption of the core NR-PKS gene (*rosJ*, Fig. 2*A*) in *A. uvarum* by replacing it with a hygromycin resistance marker (*hygR*). As we were unsure which growth conditions would induce BGC HX1012 expression, *A. uvarum* Δ*rosJ*::*hygR* was cultured alongside wild-type *A. uvarum* (empty vector control) in 12 diverse growth media (*SI Appendix, Supplementary Methods*). Liquid chromatography-mass spectrometry (LC–MS) analysis of these cultures yielded clear differential production of metabolites with [M+H]^+^ = 347.11 and [M+H]^+^ = 345.10 across the majority of conditions (representative trace in GLX media shown in Fig. 2 *B* *i* and *ii*). These metabolites were isolated and characterized as depside 4-*o*-demethyl barbatic acid (**6**, *SI Appendix*, Table S9 and Figs. S20–S23) (24) and depsidone hypoprotocetraric acid (**7**, *SI Appendix*, Table S10 and Figs. S24–S27) (25), respectively.

Based on the tailoring enzymes encoded in BGC HX1012, we reasoned that metabolites **6** and **7** would be early intermediates in the biosynthetic pathway. In addition to biosynthetic genes and a CDK homolog, BGC HX1012 also contains *rosH,* a Zn(II)_2_Cys_6_ transcription factor. Such transcription factors within BGCs have shown to be specific regulators of BGC expression, and overexpression of these transcription factors has proven an effective means of BGC activation in fungi (26–28). We employed this approach to increase the expression of all genes in the cluster in order to generate more mature cluster products. *rosH* was cloned under the control of P_coxAN_, the strong constitutive promoter of the *A. nidulans* cytochrome C oxidase, and transformed into *A. uvarum*. The resulting strain was cultured in the same 12 media used above. Across most conditions, overexpression of the cluster-specific transcription factor yielded stable or decreased production of **6** and **7** with concurrent increases in signals for related compounds **8** (*SI Appendix*, Table S11 and Figs. S28–S31) previously isolated from *Penicillium sclerotiorum* (29), and roseopurpurin C (**1**, *SI Appendix*, Table S8 and Figs. S15–S19) previously isolated from *Aspergillus fumigatus* and *Penicillium roseopurpurin* (30, 31), as well as small amounts of depsidone roseopurpurin G (**10**, *SI Appendix*, Table S12 and Figs. S32–S35), aculeatusquinone C (**11**, *SI Appendix*, Table S13 and Figs. S36–S39) (32), and roseopurpurin D (**12**, *SI Appendix*, Table S14 and Figs. S40–S43), the C-4 epimer of **1** (Fig. 2*B* and *SI Appendix*, Fig. S2).

To gain deeper insight into the biosynthesis of the roseopurpurins and to enable the production of as many BGC-associated metabolites as possible, we sought to characterize BGC HX1012 by heterologous expression in *A. nidulans*. Heterologous expression of BGC HX1012 produced only trace amounts of cluster-related metabolites. However, heterologous expression of BGC HX1035, a closely related BGC (core gene identity = 51%, Fig. 2*A*) from the genome of TTI001159, produced high levels of **1**. The observation that related BGCs will produce different amounts of metabolites when expressed in a heterologous host is not uncommon and highlights the benefit of having access to multiple related BGCs.

pRosJ, the core NR-PKS in BGC HX1035, is similar (39% amino acid identity) to DrcA, an NR-PKS involved in the biosynthesis of duricamidepside, a fungal depside related to **1**. Consistent with the activity reported for DrcA (33), we see that the heterologous expression of pRosJ alone is capable of producing 3-methylorsellinic acid and catalyzing depside bond formation to yield **6** (Fig. 2 *C*, *i*). Oxidative ether formation for conversion of **6** to depsidone **7** is subsequently catalyzed by cytochrome P450 pRosK (Fig. 2 *C*, *ii*), followed by decarboxylation by pRosB to yield compound **8** (Fig. 2 *C*, *iii*). Addition of the genes for either cytochrome P450 pRosA or reductase pRosC alone to this system yielded no additional significant products, but the concerted expression of both led to the generation of small amounts of a compound with [M+H]^+^ = 335.11 (*SI Appendix*, Fig. S3*A*). We hypothesize that this signal comes from intermediate **9**, a compound that undergoes spontaneous dehydration and rearomatization to yield **10**. Addition of the gene for *O*-methyltransferase pRosF led to the ultimate production of **1** (Fig. 2 *C*, *iv*) and its C-4 epimer **12**.

This scheme demonstrates that glyoxalase pRosD serves no obvious role in the biosynthesis of **1**. Heterologous expression of *prosD* alongside the genes necessary for the production of **1** led to the conversion of **1** to **11** by cleavage of the central ring (Fig. 2 *C*, *v* and *SI Appendix*, Fig. S3*B*).

### Roseopurpurins Are Potent Inhibitors of CDKs.

**Fig. 3.**
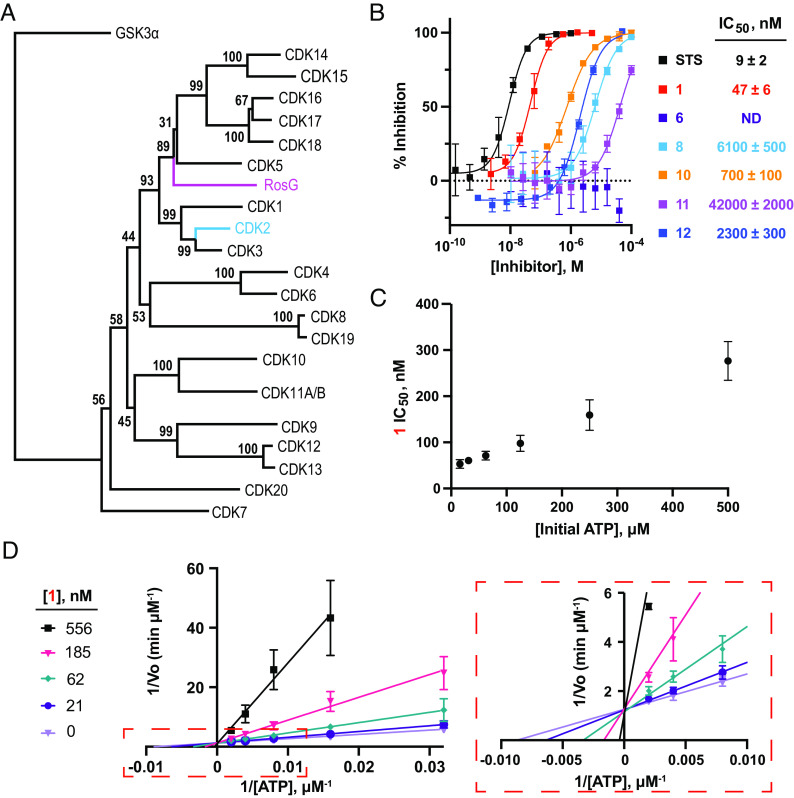
BGC HX1012/HX1035-derived metabolites are ATP-competitive CDK inhibitors. (*A*) Neighbor-joining phylogenetic tree of *A. uvarum* RosG and human CDK enzymes with 1,000 bootstrap values. GSK3α is the outgroup. (*B*) Inhibition of CDK2/cyclin E1 phosphorylation of histone H1 with BGC HX1012/HX1035-derived metabolites. Data represent the average ± the SD of the mean, n = 3. ND = not determined; inhibition did not exceed 50% at the highest concentration tested (100 µM). STS, staurosporine. (*C*) Potency of 1 in CDK2/cyclin E1 biochemical assay with varying concentrations of adenosine 5′-triphosphate (ATP). Data represent the average ± the SD of the mean, n = 4. (*D*) Lineweaver-Burk plot of 1 vs. different concentrations of ATP for CDK2/cyclin E1. A zoomed-in view of the plot is displayed in the dashed box. Data represent the average ± the SD of the mean, n ≥ 2.

**1** and **10** have been previously isolated from *A. fumigatus* and *P. roseopurpurin* and found to be generally cytotoxic, but neither their BGCs nor their molecular target were known (30, 31). Based on the presence of a putative CDK2 resistance gene (Fig. 3*A*), we hypothesized that the metabolites would be inhibitors of human CDK2. To test this hypothesis, the BGC HX1012/HX1035-derived metabolites (**1**, **6**, **8**, **10**, **11**, and **12**) were assayed for their ability to inhibit the phosphorylation of histone H1 by cyclin E1-activated CDK2 using a luciferase-coupled ADP-detection biochemical assay (ADP-Glo; Promega). Inhibition of CDK2 was observed for five of the six BGC-derived metabolites (**1**, **8**, **10**, **11**, and **12**; Fig. 3*B*), with **1** displaying the most potent inhibition (IC_50_ = 47 ± 6 nM). Intriguingly, the inhibitory activity of the compounds increased as the compounds advanced through the biosynthetic pathway. Moreover, epimerization of the 4-hydroxyl group of **1** to afford **12** resulted in an approximately 50-fold reduction in activity. To ensure that inhibition of the luminescence signal was attributable to CDK2 inhibition rather than to inhibition of the ADP-detection enzymes, control reactions where CDK2 was replaced with 10 µM adenosine 5′-diphosphate (ADP) were performed. None of the metabolites inhibited the detection of ADP in the control reactions, demonstrating that the activity was due to the inhibition of the CDK2/cyclin E1 complex (*SI Appendix*, Fig. S4).

To determine whether inhibition of CDK2 by the BGC HX1012-/HX1035-derived metabolites is a general feature of the compound class, 19 commercially available depside and depsidone natural products were screened for CDK2-inhibitory activity. None of the commercial depsidones or depsides had an IC_50_ lower than 10 µM (*SI Appendix*, Fig. S5).

### **1** Is an ATP-Competitive CDK2 Inhibitor.

We next sought to characterize the mechanism of action of **1**. The IC_50_ of **1** was within fivefold of the concentration of CDK2/cyclin E1 complex in the biochemical assay, suggesting that tight-binding inhibition may be observed under the assay conditions (34), which would complicate mechanism of action studies. To test for tight binding inhibition under the assayed conditions, we determined the IC_50_ of **1** at varying concentrations of CDK2/cyclin E1. Indeed, tight-binding inhibition was observed at concentrations of cyclin E1-activated CDK2 greater than 7.5 nM (*SI Appendix*, Fig. S6*A*). To remove confounding effects of tight binding inhibition, **1** was checked for ATP-competitive inhibition of cyclin E1-activated CDK2 with 5 nM enzyme. Consistent with an ATP-competitive mechanism of inhibition, the IC_50_ of **1** was dependent on the concentration of ATP (Fig. 3*C* and *SI Appendix*, Fig. S6*B*). Moreover, kinetic curves revealed that treatment with **1** increases the *K*_M_ for ATP without affecting *k*_cat_ (Fig. 3*D* and *SI Appendix*, Fig. S6 *C* and *D*). Fitting the kinetic curves to a competitive inhibition model using PRISM resulted in a *K*_iapp_ for **1** of 44 nM ± 8 nM, which is consistent with the observation that tight-binding inhibition is observed at enzyme concentrations greater than 7.5 nM.

### **1** Inhibits CDK2 in Human Cell Lines and Induces Cell-Cycle Arrest.

**Fig. 4.**
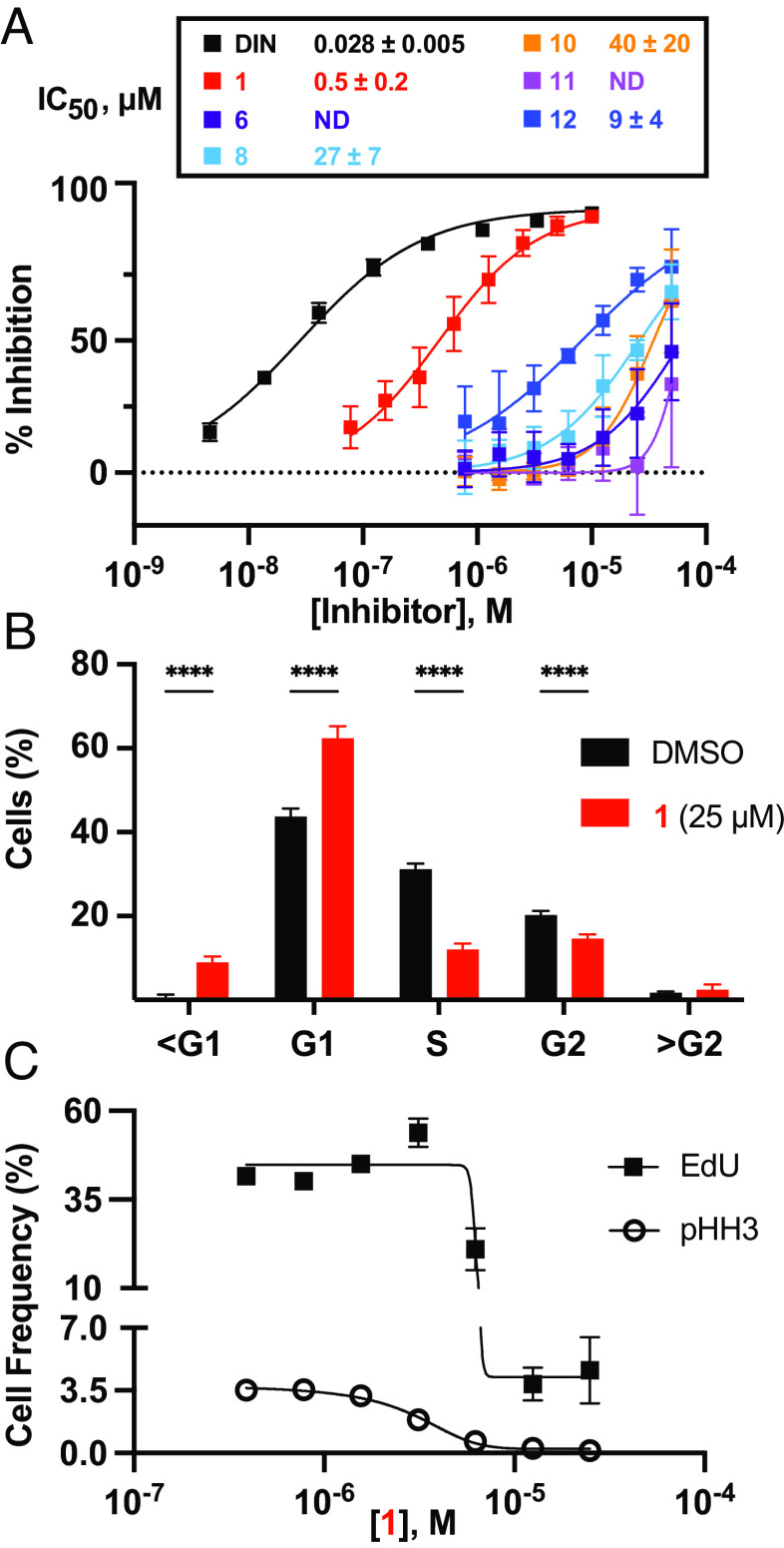
1 inhibits CDKs in human cells, inducing cell cycle arrest. (*A*) Inhibition of luciferase-CDK2/cyclin E1 nanoBRET signal with BGC HX1012/HX1035-derived metabolites. Data represent the average ± the SD of the mean, n = 6. ND = not determined; inhibition did not exceed 50% at the highest concentration tested (50 µM). DIN, dinaciclib. (*B*) Cell cycle distribution measured by DAPI after 24 h treatment with 25 µM 1 compared to dimethyl sulfoxide (DMSO) in synchronized HCT116 cells (means ± SD; n = 6). ****P < 0.0001, two-way ANOVA with Sidak post hoc test. (*C*) EdU and pHH3 incorporation measured by flow cytometry with varied concentrations of 1 in synchronized HCT116 cells (means ± SD; n = 6).

To determine whether the BGC HX1012/HX1035-derived metabolites can engage with CDK2 in a cellular context, we profiled the activity of **1**, **6**, **8**, **10**, **11**, and **12** in a nanoBRET-based CDK2 engagement assay (35) in HEK293 cells. We observe inhibition of CDK2 in cells and consistent with the biochemical assay data, **1** was the most potent inhibitor among the BGC HX1012/HX1035-derived metabolites (IC_50_ = 500 ± 200 nM; Fig. 4*A*).

CDKs are critical regulators of the eukaryotic cell cycle and their inhibition causes disruptions to cell cycle progression. Depending on the cyclin-bound state, CDK2 promotes S-phase entry (cyclin E-bound) and drives the S/G2 transition in the cell cycle (cyclin A-bound) (36). Based on the biochemical and nanoBRET data demonstrating **1** is a potent inhibitor of CDK2, we expected the metabolite to induce G1 phase arrest in G0 synchronized cells. To test this, HCT116 cells were synchronized at G0 by serum starvation (*SI Appendix*, Fig. S7 *A* and *B*) and released into serum-containing medium containing **1**. For comparison, control treatments were performed with known G1 arrest-inducing CDK inhibitors (flavopiridol, KO3861, dinaciclib, and palbociclib) and a G2/M arrest-inducing microtubule polymerization inhibitor (nocodazole). Following a 24-h inhibitor treatment, cell cycle stages were assessed by measuring DNA content (G0/G1, S, and G2/M phase alterations), EdU incorporation (S phase alterations), and histone H3 Ser10 phosphorylation (pHH3) levels (M phase alterations) via flow cytometry. Based on DNA content, treatment with 25 µM **1** resulted in a significant increase in the percentage of cells in the G1 stage and a corresponding decrease in the percentage of cells in the S and G2 stages compared to a DMSO-treated control (Fig. 4*B* and *SI Appendix*, Fig. S7*C*). Consistent with these results, treatment with **1** also significantly reduced the percentage of EdU and pHH3 positive cells in a dose-dependent manner (Fig. 4*C* and *SI Appendix*, Fig. S7*D*) suggesting that the metabolite prevents cells from entering S phase. Similar results were obtained in cells treated with the known G1-arresting CDK inhibitors flavopiridol, dinaciclib, KO386, and palbociclib but not in cells treated with the G2-arresting microtubule inhibitor nocodazole (*SI Appendix*, Fig. S7 *C* and *D*).

Taken together, these data demonstrate that **1** is cell permeable and inhibits CDK2 in a cellular environment, thereby arresting cells in the G1 phase of the cell cycle.

### **1** Is CDK-Selective Type 1 Kinase Inhibitor.

**Fig. 5.**
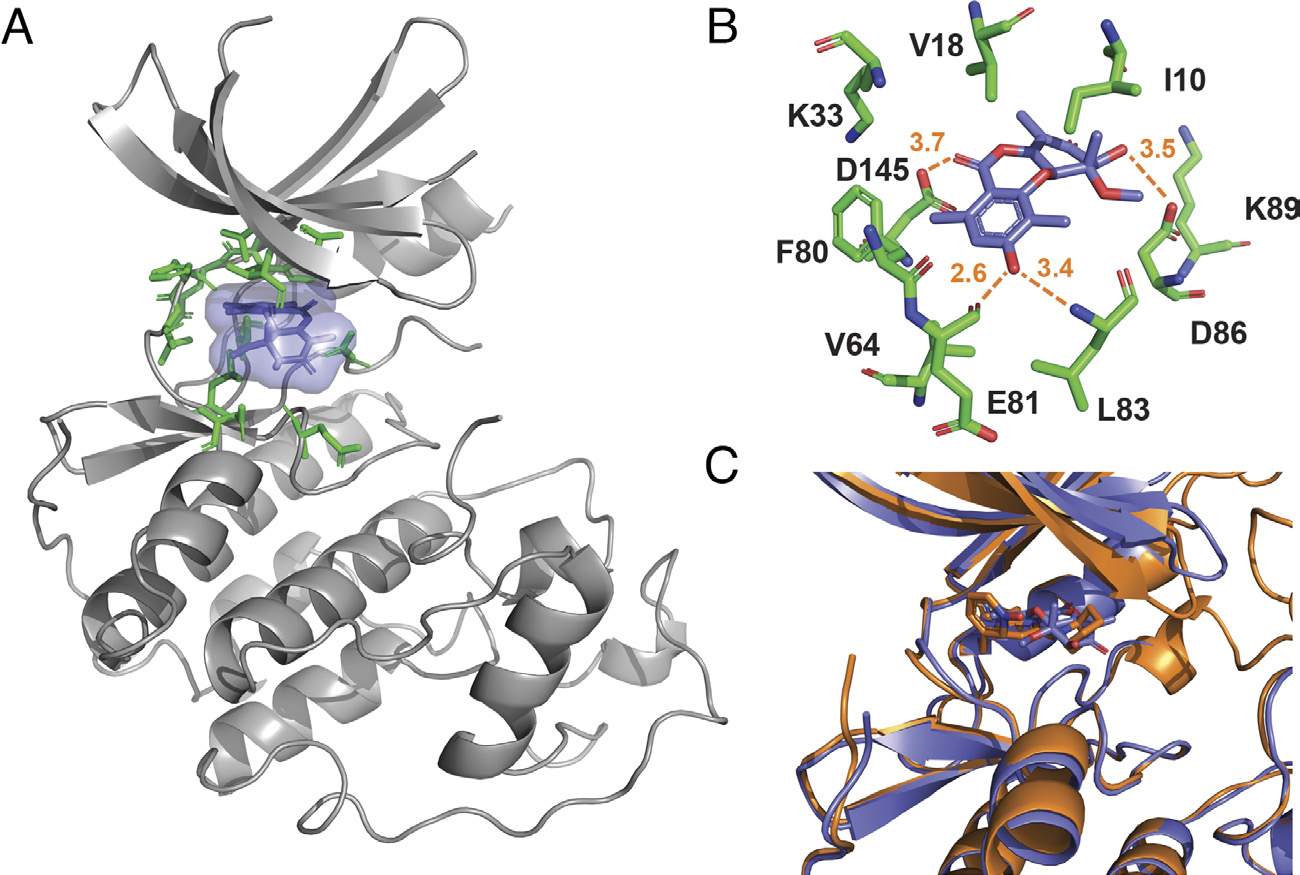
1 binds to the ATP-binding pocket of CDK2. (*A*) Structure of **1** (blue) bound to CDK2. Residues in the ATP-binding pocket are colored green. (*B*) Zoomed-in view of ATP-binding site with key **1**-binding residues displayed. (*C*) Overlay of **1**:CDK2 (blue) and dinaciclib:CDK2 (orange; PDB 4KD1).

To obtain a deeper understanding of the mechanism of CDK2 inhibition by **1**, we determined the co-crystal structure of **1** bound to CDK2 at a resolution of 2.62 Å. The co-crystal structure revealed that **1** is bound to the ATP-binding pocket, consistent with the ATP-competitive mechanism of inhibition (Fig. 5*A*). The 3′-OH group of **1** formed hydrogen bonds with the amide backbone of Glu81 and Leu83 in the hinge region of CDK2 (Fig. 5*B*). Notably, the hydrogen bond with Glu81 is unusually short, 2.6 Å, suggesting its energetic favorability. The structure also revealed key hydrogen bond interactions between the 4-OH and the 9’-carbonyl with Asp86 and Asp145, respectively (Fig. 5*B*). These critical contacts in the ATP-binding pocket with the 4-OH, as well as the 9′-carbonyl, explain the potency differences observed with the intermediate/shunt metabolites produced by BGC HX1012/HX1035 (Figs. 3*B* and 4*A*).

Next, we compared the binding mode of **1** with the crystal structures of five type I ATP-competitive inhibitors (dinaciclib, SU9516, CGP74514A, AZA-5438, and purvalanol B) and one type II inhibitor (K03861) bound to CDK2 (*SI Appendix*, Fig. S8; PDB: 4KD1, 3PY0, 6GUK, 6GUH, 1CKP, and 5A14). Similar to the type I ATP-competitive kinase inhibitors, **1** makes favorable interactions with the kinase hinge region. Indeed, the binding mode of **1** was similar to that of dinaciclib (Fig. 5*C* and *SI Appendix*, Fig. S8) (37, 38) despite sharing no chemical similarity or conserved pharmacophore-protein interactions.

Hinge-binding, ATP-competitive inhibitors are the predominant class of kinase antagonists identified to date (39). However, given the conservation in protein sequence and structure of the ATP-binding pocket across the kinase superfamily, selectivity is a significant challenge for compounds with an ATP-competitive mechanism of action. Given the relatively small size of **1** and the common mechanism of inhibition, we anticipated that kinase selectivity for **1** would be limited. To evaluate selectivity, **1** was assayed for its ability to inhibit a panel of 30 diverse kinases spanning seven kinase families (*SI Appendix*, Fig. S9) using a luciferase-dependent ADP-detection assay (Table 1 and *SI Appendix*, Fig. S10). For reference, the pan-kinase inhibitor staurosporine was also profiled (Table 1 and *SI Appendix*, Fig. S11). Of the 30 kinases profiled, **1** inhibited seven with a nanomolar IC_50_. Of these, five were in the CDK family, with CDK2 being the most sensitive (potency shift relative to data in Fig. 3*B* is due to tight-binding inhibition). Moreover, in contrast to the pan-CDK inhibitors dinaciclib and flavopiridol, **1** shows modest selectivity for CDK2 over CDK1 and strong selectivity for CDK2 over CDK9 (36, 40), both of which are associated with toxicity (16). Outside of the CDK family, only AMPK and CAM4K were inhibited with nanomolar IC_50_s. This starkly contrasts staurosporine, which inhibited 24 kinases with a nanomolar IC_50_ and showed no kinase family selectivity. These data indicate that despite the small size of **1** and common mechanism of action, selectivity for CDK proteins evolved, consistent with the phylogeny of the putative CDK resistance gene.

**Table 1.** Kinase selectivity of **1** and staurosporine

	IC_50_ ± STDEV (nM)			IC_50_ ± STDEV (nM)	
	1 (n = 3)	Staurosporine (n = 2)		1 (n = 3)	Staurosporine (n = 2)
CDK1/Cyclin A2	950 ± 160	3 ± 1	FGFR1	8,000 ± 5,000	<1
CDK2/Cyclin E1	160 ± 20	43 ± 6	JAK3	>50,000	<1
CDK3/Cyclin E1	370 ± 150	37 ± 2	LCK	30,000 ± 10,000	8 ± 3
CDK5/p35	260 ± 160	<1	SYK	12,000 ± 3,000	<1
CDK6/Cyclin D3	720 ± 180*	150 ± 40*	MINK1	>50,000	6.0 ± 1
CDK9/Cyclin K	19,000 ± 3,000	20 ± 7	PAK1/CDC42	>50,000	60 ± 30*
CLK3	2,900 ± 400	1,200 ± 100	IRAK4	>50,000	15 ± 7
Aurora A	13,000 ± 3,000	11 ± 4	TAK1-TAB1	17,000 ± 4,000	8 ± 1
CK2α1	4,000 ± 1,000	>20,000	GSK3β	1,100 ± 200	8 ± 2
IKKβ	>50,000	>20,000	p38α	>50,000	>20,000
CK1α1	1,200 ± 200	>20,000	AMPK α1/β1/γ2	100 ± 15	<1
CK1γ1	>50,000	>20,000	CAMK4	600 ± 200	21 ± 2
PKCα	>50,000	<1	CHK1	>50,000	5 ± 1
ROCK1	23,000 ± 4,000	4 ± 1	DAPK1	>50,000	98 ± 7
AKT1	6,000 ± 3,000	18 ± 7	MAPKAPK2	>50,000	160 ± 30

IC_50_ values represent the averages from the indicated number of experiments ± SD of the mean. Asterisk denotes enzymes where <70% maximal inhibition was observed.

## Discussion

Resistance gene–guided genome mining provides a means of identifying bioactive metabolites and linking them to their protein target without the extensive time, labor, and cost associated with traditional “grind-and-find” natural products discovery. As demonstrated here, this approach can also provide an advanced starting point for drug discovery as the metabolites have already been evolutionarily engineered to interact with their target. Additional structure–activity relationship information can be obtained by screening precursor metabolites and shunt products for binding and bioactivity, resulting in a well-developed data package prior to initiating any additional work (i.e., a medicinal chemistry campaign).

The availability of large numbers of microbial genomes presents opportunities for resistance gene–guided genome mining. The number of BGCs is large: up to 100 per genome, across thousands of genomes. Here, we apply this technique to reveal the molecular targets of the known compound **1**; demonstrating that it is a potent and specific inhibitor of the CDKs.

To identify natural product inhibitors of human CDK2, we looked for BGCs containing a putative resistance gene with homology to human CDKs, ultimately identifying the roseopurpurin BGC in the genomes of *A. uvarum*, *Phaeosphaeria* sp. TTI001159 and five additional publicly available fungal genomes (*SI Appendix*, Fig. S1). In order to identify the products of these clusters, we first characterized this BGC by in situ engineering, generating mutants with the core NR-PKS knocked out and the BGC-specific transcription factor overexpressed. The results from these in situ engineering studies facilitated the identification of a series of BGC products and provided insights into the possible regulation of this BGC. The titers of early pathway intermediates **6** and **7** are detectable across all conditions tested and are influenced to a relatively small extent by transcription factor overexpression. **1**, the most bioactive cluster product, is produced by the wild-type strain at significant levels in only a few growth conditions and its titer is increased significantly, in some cases several orders of magnitude, upon transcription factor overexpression (*SI Appendix*, Fig. S2). These results suggest that the regulation of this BGC is not as simple as it being “on” or “off”, as transcriptional control appears much tighter for those enzymes responsible for the later steps in the pathway.

Further characterization of this cluster by heterologous expression in *A. nidulans* yielded additional insights. In addition to establishing a series of transformations ultimately leading to the formation of **1**, we found glyoxalase RosD leads to the conversion of **1** to **11**. **11** proved significantly less potent than **1** in both biochemical and cellular assays (Figs. 3*B* and 4*A*), leading us to hypothesize that the primary role of RosD is detoxification. This BGC also contains *rosJ*, a gene encoding a MFS drug pump; a feature common in fungal BGCs and that has been shown to be a mechanism of self-protection (41) in addition to the putative resistance gene *rosG*.

While this work was in progress, a report emerged of human CDK2 inhibition by **1** and **12** isolated from cultures of *A. fumigatus*, a species whose genome contains the rosepurpurin gene cluster (42), corroborating our observations that these metabolites are inhibitors of human CDK2. By profiling the activity of **1** against a panel of human kinases, we demonstrate that it is both potent and quite specific for the CDK1/2/3, CDK5, and CDK4/6 kinase subfamilies. That we see good selectivity for these CDK subfamilies, but only modest selectivity within them is likely caused by the significant divergence in the evolution of the CDKs between fungi and humans and the ATP-competitive mechanism of inhibition. Most fungal species have only 6 CDK paralogs, while the human genome has 20. Indeed, the entire CDK1/2/3/4/6 and CDK5 subfamilies can each be traced back to a single homolog in fungi, represented by CDC28 and Pho85 in *S. cerevisiae*, respectively (15). Outside of the CDK branch of the CMGC kinase family, nanomolar inhibition of two CAMK family Ser/Thr kinases, AMPKα1 and CAMK4 was observed (Table 1 and *SI Appendix*, Figs. S9 and S10). This potent inhibition can likely be explained based on alignments of the **1**-CDK2 structure with published AMPKα1 (PDB 4CFF) and CAMK4 structures (PDB 2W4O), which reveal that the key **1**-CDK2 binding interactions are maintained with both proteins despite significant sequence divergence in the ATP-binding pockets (*SI Appendix*, Figs. S12–S14).

The structure of **1**-bound CDK2 revealed that ATP-competitive inhibition was achieved through binding to the ATP pocket of CDK2 and provided a structural rationale for the preliminary structure–activity relationships obtained through profiling precursor metabolites and shunt products in the **1** maturation pathway. Three key hydrogen-bonding interactions were identified in the structure. First, the 3′-OH group of **1** interacts with the amide backbone of Glu81 and Leu83 in the hinge region of CDK2. Similar hinge-region interactions are observed for structurally diverse natural products with ATP-competitive kinase inhibitory activity (43) and synthetic kinase inhibitors, including dinaciclib (39). Second, the 4-OH group of **1** forms a hydrogen bond with the side chain of Asp86. Epimerization of the 4-OH in **1** to afford **12** results in a 50-fold reduction in potency (Fig. 3*B*). Furthermore, comparison of the potency of **8** (which lacks the 4-OH) and **7**, reveals that the addition of this critical hydrogen-bond donor improves potency by approximately ninefold (Fig. 3*B*). Third, the 9′-carbonyl of **1**, forms a hydrogen bond with Asp145. The coordination of this central ring potentially helps orient the metabolite in the binding site. Consistent with this prediction, **11** is approximately 900-fold less active than **1**.

**1** represents a new scaffold for type 1 kinase inhibitors of CDK proteins. The potency and specificity of its activity demonstrate the utility of resistance gene–guided genome mining to identify metabolites with bioactivities relevant to human disease. Combined with the initial SAR that arises from profiling intermediate metabolites in the biosynthetic pathway, this work demonstrates the ability of this approach to yield advanced starting points for drug discovery campaigns. Furthermore, fungi and humans share thousands of genes with significant homology (44, 45), giving resistance gene–guided genome mining of fungal genomes the potential to generate inhibitors of promising targets across a wide range of disease areas.

## Materials and Methods

### Reagent Sourcing.

Unguinol, leoidin, nornidulin, stictic acid, nidulin, lobaric acid, salazinic acid, penicillide, galbinic acid, 7-chlorofolipastatin, folipastatin, and psoromic acid were sourced from Cayman chemicals. Gangaleoidin, lecanoric acid, evernic acid, atranorin, dihydroatranorin, and fumarprotocetraric acid were sourced from Microsource Discovery Systems. Dinaciclib was sourced from MedChemExpress. Staurosporine was sourced from LC labs. All compounds were dissolved in DMSO and used without further purification.

### Strains and Fermentation Conditions.

*A. uvarum* (CBS121591, Country of origin: Italy) was obtained from the Centraalbureau voor Schimmelcultures (CBS) fungal collection through the Westerdijk Fungal Biodiversity Institute. *A. uvarum* was grown on Difco™ PDA (Potato Dextrose Agar) Plates from BD biosciences for activation. Strain TTI001159 (*Phaeosphaeria* sp.) was obtained through collaboration with the lab of Gerald Bills at the University of Texas Health Science Center. BgHX18 (pyrG89; pyroA4; nkuA::argB; riboB2; easA::CRISPR; tdiA::CRISPR; stcA::CRISPR,easb::pyrG89), bgHX16 (pyrG89; pyroA4; nkuA::argB; riboB2; easA::pyroA; tdiA::CRISPR; stcA::CRISPR), and bgHX17 (pyrG89; pyroA4; nkuA::argB; riboB2; easA::CRISPR; tdiA::CRISPR; stcA::CRISPR) are modified versions of *A. nidulans* A1145 purchased from the Fungal Genetics Stock Center and used for heterologous expression of genes from TTI001159. *Escherichia coli* strain DH10B was used for cloning. *Saccharomyces cerevisiae* strain JHY692 (MATa his3Δ1 leu2Δ0 ura3Δ0 met15Δ0 SAL1+ HAP1+ CAT5(91M) MIP1(661T) MKT1(30G) RME1(INS-308A) TAO3(1493Q) prb1Δ pep4Δ ADH2p-npgA-ACS1) was used for homologous recombination of DNA fragments to assemble the vectors used in heterologous expression.

### Plasmid Construction for Heterologous Expression in *A. nidulans* and *A. uvarum*.

Plasmids for heterologous expression were constructed as described previously (46). Briefly, plasmids pHex315, pHex318, and pHex344 were used as vectors to insert genes which contain auxotrophic markers for riboflavin (riboB), uracil (pyrG), and pyridoxine (pyroA), respectively. All genes were designed as fully spliced sequences and ordered as gene fragments from Twist Bioscience. Plasmids pHex315, pHex318, and pHex344 contain auxotrophic markers for riboB, pyrG, and pyroA, respectively, and served as the backbone vectors for all plasmid assembly. Vectors were linearized by digestion with pacI, and gene fragments for assembly were generated as PCR products using synthetic gene fragments as templates and the primers defined in *SI Appendix*, Table S1. The plasmids described in *SI Appendix*, Table S2 were assembled by yeast homologous recombination by combining 0.05 pmol each of the prepared fragments and transforming them in JHY692 using the Frozen-EZ Yeast Transformation II Kit™ (Zymo Research).

Gene fragments for construction of gene knockouts and transcription factor overexpression strains in *A. uvarum* were amplified using PCR genomic DNA as a template. For transcription factor overexpression, *rosH* was cloned under the constitutive promoter, P_coxAN_ in the pHex385 backbone. The pHex385 backbone contains a hygromycin selectable marker (hph) and the AMA1 fungal origin of replication.

### Transformation and Heterologous Production in *A. nidulans* and *A. uvarum*.

Spores of *A. nidulans/A. uvarum* were inoculated into 25 mL YG medium and incubated in stationary culture at 30 °C overnight. After incubation, the biomass was collected using sterilized tweezers and washed with 10 mL of water twice. The germlings were then transferred into 25 mL of digestion buffer (Supplementary methods) containing 3 g of VinoTaste^®^ Pro (Novozymes) and 100 mg of Yatalase™ Enzyme (Takara Bio Cat#T017). The digestion was incubated for 5 h at 30 °C and 80 rpm. The digestion mix was poured into a 50-mL conical tube and overlaid with 10 mL of 0.4 M ST Buffer (*SI Appendix, Supplementary Methods*) and centrifuged at 1,800 × g for 15 min. The protoplasts were then removed from the interface of the two buffers and transferred to sterile tubes. The protoplasts were washed with 0.6 M KCl, collected by centrifugation (1,800 × g for 10 min), and resuspended in Transformation Buffer (*SI Appendix, Supplementary Methods*). DNA was added to the transformation mix and incubated on ice for an hour. PEG solution (60% w/v PEG 3350, 50 mM CaCl_2_, and 50 mM Tris-HCl) was added to the protoplast solution and incubated at room temperature for 20 min. The cells were then plated onto solid SMM (with the addition of 100 µg/µL hygromycin for *A. uvarum*). After transformants appeared on the plates, the *A. nidulans/A. uvarum* spores were restreaked onto solid GMM production medium at 37 °C for 3 d. The spores were collected with 0.01% Tween 80 solution and transferred into liquid GMM media for metabolite production. *A. uvarum* strains were grown in a panel of 12 growth media (*SI Appendix, Supplementary Methods*).

### Sample Analysis of *A. nidulans* Transformants and *A. uvarum* Extracts.

Strains were grown in 96 well deep well plates with 500 µL of media. *A. nidulans* was grown at 30 °C for 3 d and *A. uvarum* was grown at 30 °C for 7 d. The plate was then frozen and lyophilized. The samples were then resuspended in 1:1 MeOH and injected onto the LC-MS for analysis.

### Kinase Phylogeny and Structural Alignments.

Kinase sequences were collected from UniProt (*SI Appendix*, Table S5), trimmed to include only the kinase domain in the alignment, and aligned using MAFFT v7 (https://mafft.cbrc.jp/alignment/server/) (47) using the default settings. The phylogenetic trees were reconstructed using the neighbor-joining method with 1,000 bootstrap replicates.

Structural alignments between **1**-bound CDK2 (PDB: 8OY2) and either dinaciclib-bound CDK2 (PDB: 4KD1), SU9516-bound CDK2 (PDB: 3PY0), CGP74514A-bound CDK2 (PDB: 6GUK), AZA-5438-bound CDK2 (PDB: 6GUH), purvalanol B-bound CDK2 (cyan; 1CKP), K03861-bound CDK2 (PDB: 5A14), AMPKα1 (PDB: 4CFF), or CAMK4 (PDB: 2W4O) were generated using TM-align (48). The aligned structures were visualized using PyMOL (Schrödinger).

### CDK2/Cyclin E1 ATPase Assay.

Active CDK2/cyclin E1 complex (baculovirus expressed in sf9 insect cells), histone H1 (purified from calf thymus tissues), and the ADP-Glo reagents were purchased from Promega. Typical reactions (20 µL final volume) contained 15 nM active CDK2/cyclin E1 complex, 0.1 mg/mL histone H1, 150 µM ATP, 20 mM MgCl2, 0.1 mg/mL BSA, 50 μM Dithiotheritol (DTT), and 5% DMSO (± inhibitor) in 40 mM TRIS buffer at pH 7.5. Following a 30-min preincubation of the enzyme and inhibitor in the reaction mixture at room temperature, reactions were initiated with the addition of ATP and histone H1. Kinase reactions were carried out at room temperature for 30 min before reactions were quenched with the addition of one volume of ADP-Glo™ reagent (Promega). Following a 60-min incubation at room temperature to remove excess ATP, two volumes of Kinase Detection Reagent (Promega) were added to the sample. The luminescence signal was allowed to develop for 30 min before being measured on a Spark multimode plate reader (Tecan). The percent inhibition was calculated by the comparison of the signal to DMSO (0% inhibition) and 55 nM dinaciclib (100% inhibition) controls. IC_50_ values were calculated from the dose–response curves using Prism 9 (four-parameter, variable slope).

Tight binding inhibition assays were performed as above except that the concentration of active CDK2/cyclin E1 complex was varied.

### ADP-Glo Counter Screen.

Reactions (20 µL) were performed with 140 µM ATP, 10 µM ADP, 20 mM MgCl2, 0.1 mg/mL BSA, 50 μM DTT, and 5% DMSO (± inhibitor) in 40 mM TRIS buffer at pH 7.5. Following a 30-min preincubation of the mixture, the ADP levels were quantified using the ADP-Glo kinase detection assay described above.

### Mechanism of Inhibition Studies.

Reactions (20 µL final volume) were performed with 5 nM active CDK2/cyclin E1 complex, 20 mM MgCl2, 0.1 mg/mL BSA, 50 μM DTT, and 5% DMSO (± inhibitor) in 40 mM TRIS buffer at pH 7.5. ATP and inhibitor concentrations were varied. Following a 30-min preincubation of the enzyme, inhibitor, and ATP in buffer at room temperature, reactions were initiated with the addition of histone H1 peptide (Promega) to a final concentration of 0.19 mg/mL. Kinase reactions were carried out at room temperature for 40 min before reactions were quenched with the addition of one volume of ADP-Glo™ reagent (Promega) reagent. Following a 60-min incubation at room temperature to remove excess ATP, two volumes of Kinase Detection Reagent (Promega) were added to the sample. The luminescence signal was allowed to develop for 30 min before being measured on a Spark multimode plate reader (Tecan). For each concentration of ATP, a DMSO control (0% inhibition) and a 5 µM **1** control (100% inhibition) were run. Samples were blanked to the 5 µM **1** control to account for the presence of contaminating ADP in the ATP stock. ADP levels in reactions were determined by the comparison of the blank-corrected samples to an ADP standard curve. IC_50_ values were calculated from the dose–response curves using the variable slope (four-parameter) model in Prism 9. The *K*_iapp_ was calculated from the kinetic curves using the competitive inhibition model in Prism 9.

### Kinase Selectivity ATPase Assay.

Active kinases and their corresponding substrates were sourced from Promega as part of the CMGC kinase (catalog number: V6856) and general kinase (catalog number: V6928) panels. Reactions (10 µL final volume) were performed according to the manufacturer’s protocol with a 30-min compound preincubation with active kinase at room temperature in the reaction mixture. Reactions were initiated by the addition of ATP (10 µM final) and kinase substrate. Following a 60-min reaction at room temperature, reactions were quenched with the addition of one volume of ADP-Glo™ reagent (Promega) reagent. Following a 60-min incubation at room temperature to remove residual ATP, two volumes of Kinase Detection Reagent (Promega) were added to the sample. The luminescence signal was allowed to develop for 30 min before being measured on a Spark multimode plate reader (Tecan). The percent inhibition was calculated by the comparison of the signal to DMSO (0% inhibition) and no-enzyme (100% inhibition) controls. IC_50_ values were calculated from the dose–response curves using Prism 9 (four parameter, variable slope).

### NanoBRET CDK2/Cyclin E1 Intracellular Target Engagement Assay.

NanoBRET was performed using the NanoBRET Target Engagement Intracellular Kinase Assay (Promega) in HEK293 cells (ATCC CRL-1573). HEK293 cells were cultured in Roswell Park Memorial Institute (RPMI) (Invitrogen) with 10% fetal bovine serum (FBS; Invitrogen). For transfection, HEK293 cells were trypsinized and resuspended in Opti-MEM I Reduced Serum, no phenol red medium (Invitrogen) with 1% FBS at a concentration of 2 × 10^5^ cells/mL. Five hundred microliters of a 10 μg/mL solution of DNA was prepared by adding 1 μg/mL of CDK2-NanoLuc Fusion Vector (Promega; NV2781) to 9 μg/mL of CCNE1 Expression Vector (Promega; NV2641) in Opti-MEM I Reduced Serum, no phenol red media. Thirty microliters of FuGENE HD Transfection Reagent (Promega) was added to each milliliter of DNA mixture. The lipid-DNA mixture was incubated at room temperature for 20 min. Postincubation, 10 mL of cells was added to the lipid–DNA mixture and plated in a 96-well clear bottom white plate (Corning 3903) at a concentration of 2 × 10^4^ cells/well. Cells were incubated at 37 °C with 5% CO_2_ for 20 to 30 h.

Posttransfection, the media were replaced with fresh Opti-MEM I Reduced Serum, no phenol red medium. NanoBRET Tracer reagent K-10 (NV2641) was added at a concentration of 50 μM (1% DMSO final) along with 0.2% DMSO (± inhibitor). Plates were incubated at 37 °C with 5% CO_2_ for 2 h. Following incubation, plates were removed from the incubator and allowed to equilibrate to room temperature for 15 min. A 3× Complete Substrate Plus Inhibitor Solution was made by adding 30 μL of NanoBRET Nano-Glo Substrate (Promega), 10 μL of Extracellular NanoLuc Inhibitor (Promega), and 4,960 μL of Opti-MEM reduced serum medium, no phenol red (Invitrogen). Fifty microliters of 3× Complete Substrate Plus Inhibitor Solution was added to each well, and the BRET signals (450 nm donor emission; 610 nm acceptor emission) were read on a GloMax Discover plate reader (Promega). A raw BRET ratio was generated by dividing the acceptor emission value by the donor emission value for each sample and compared to DMSO (0% inhibition) and 10 µM dinaciclib (100% inhibition) controls. IC_50_ values were calculated from the dose–response curves using Prism 9 (four-parameter, variable slope). Experiments were performed in triplicate with technical duplicates in each run.

### Cell Cycle Analysis Assay.

HCT-116 (ATCC CCL-247) cells were grown in DMEM + GlutaMAX (Thermo Fisher Scientific 10566-016) supplemented with 10% FBS (Thermo Fisher Scientific 16140-071) at 37 °C with 5% CO_2_. For synchronization, cells were seeded in 96-well microtiter plates at a density of 1.5 × 10^5^ cells/ml in DMEM + GlutaMAX supplemented with 0.5% FBS for 96 h. After 96 h, the media were replaced with DMEM + GlutaMAX with 10% FBS, and test compounds in DMSO were added. DMSO levels were normalized. Cells were incubated for 24 at 37 °C and 5% CO2. Two hours prior to harvest, 20 µM of EdU was added from the Click-iT Plus EdU Flow Cytometry Assay Kit (Thermo Fisher Scientific C10633) to the media and incubated at 37 °C and 5% CO_2_. After 2 h, the media were removed, and cells were washed once with PBS and trypsinized. Cells were collected in a v-bottom 96-well plate and spun at 300 g for 5 min and washed again with PBS. Cells were fixed and permeabilized with eBiosciences FoxP3/Transcription factor fixation kit (Thermo Fisher Scientific 00-5523-00) for 20 min. After fixation, cells were spun at 800 × g and washed twice with permeabilization buffer. Click-iT Plus reaction cocktail was added and incubated for 30 min in the dark at room temperature. Cells were washed twice with permeabilization buffer and spun at 800 × g. Cells were stained with pHH3 for 30 min in the dark at room temperature. Cells were washed twice with permeabilization buffer and spun at 800 × g. FxCycle Violet (Thermo Fisher Scientific F10347) was added at a concentration of 1 µg/mL for 30 min at room temperature in the dark. After incubation, cells were immediately acquired on a MACSQuant 10 cytometer (Miltenyi). Analysis was performed with Flowjo (BD Biosciences). The Dean–Jett Fox algorithm was used for cell cycle staging. *P* values were calculated using a two-way ANOVA with Sidak post hoc test in Prism 9. Experiments were performed in triplicate with technical duplicates in each run.

### CDK2 Crystallization.

Recombinant human CDK2 was expressed with an N-terminal histidine tag in *E. coli*. Purification was performed by nickel affinity and size exclusion chromatography. Final protein was concentrated to 20 mg/mL in a buffer containing 20 mM Tris HCl pH 8.0, 150 mM NaCl, and 2mM DTT for crystallography. Apo crystals of CDK2 were grown by vapor diffusion at 4 °C, mixing equal volumes of protein and well solution (MES pH 7.0, PEG3350, ammonium acetate). Crystals grew within 3 to 5 d and were soaked with **1** at a final concentration of 10 mM overnight. Crystals were cryoprotected with 15% glycerol and flash-frozen in liquid nitrogen.

Data of CDK2-**1** crystal was collected at the SWISS LIGHT SOURCE (SLS, Villigen, Switzerland). Data were processed using the software autoPROC, XDS, and AIMLESS (49–53). The crystals belong to the space group P 21 21 21 with one monomer in the asymmetric unit. The phase information was obtained by molecular replacement, using the coordinates of a previously solved CDK2 structure as a search model. Subsequent model building and refinement were performed according to standard protocols with COOT (54) and the software package CCP4 (52), respectively. Model quality was evaluated with MolProbity (55). Data collection and refinement statistics are provided in Supplementary Information *SI Appendix*, Tables S6 and S7. The structure was visualized using PyMOL (Schrödinger).

### Isolation of Compounds.

Cultures were extracted by liquid:liquid extraction with 1 volume of ethyl acetate. After shaking for 2 h, extracts were filtered, and the organic phase was collected and dried. The crude extract was subjected to HPLC fractionation using a semipreparative Phenyl column (XBridge BEH Phenyl OBD, 10 × 250 mm, 5 µm), with a gradient method using 0.1% aqueous formic acid (A)/acetonitrile with 0.1% formic acid (B) at 4 mL/min (5% B for 1 min; 5 to 40% B in 3 min; 40 to 100% B in 10 min; hold 100% B for 7 min). The collected fractions were analyzed using liquid chromatography-high resolution mass spectrometry (LC-HRMS) using 0.1% aqueous formic acid (A)/acetonitrile with 0.1% formic acid (B) with a linear gradient of 12.5 to 100% B in 5 min, and desired fractions were pooled. They were further fractionated using a semipreparative C18 column (XBridge BEH Shield RP18 OBD, 10 × 250 mm, 10 μm) with gradient method (35% for 3 min; 35 to 75% in 41 min; 75 to 100% in 1 min; hold 100% for 6 min) using the same solvents as above, at 4 mL/min, yielding **1** (r.t. 14.2 min, 8.0 mg), **6** (r.t. 36.8 min, 4.2 mg), **7** (r.t. 27.2 min, 1.4 mg), **8** (r.t. 23.7 min, 1.2 mg), **10** (r.t. 17.8 min, 2.2 mg), **11** (r.t. 7.7 min, 2.3 mg), and **12** (r.t. 16.2 min, 2.0 mg).

HPLC purifications were performed using a Thermo Vanquish Flex HPLC. HPLC-HRMS analyses were acquired using a Thermo Vanquish HPLC system coupled to a Q-Exactive Orbitrap high-resolution mass spectrometer at 120 K resolution for MS1 and 17.5 K resolution for MS/MS. For elucidation of chemical structures, 1D and 2D NMR spectra were obtained on Bruker AV600 spectrometer (^1^H 600 MHz, ^13^C 150 MHz) equipped with a BBO cryoprobe. Chemical shifts were referenced using the solvent peak or TMS (δ 0).

## Supplementary Material

Appendix 01 (PDF)

## Data Availability

Coordinates and structure factors for the CDK2-bound roseopurpurin C (**1;** deposited to PDB as HB-29260) structure were deposited to the PDB with the accession number 8OY2 (56). The sequence of gene cluster HX1035 was deposited in NCBI with the accession number OR063823 (57). All primer and plasmid sequences and compound characterization data are included in *SI Appendix*.
