# Supplementary material for: Resistance gene–guided genome mining reveals the roseopurpurins as inhibitors of cyclin-dependent kinases
Source: Proc Natl Acad Sci U S A. 2023 Nov 20;120(48):e2310522120. doi: 10.1073/pnas.2310522120 (PMC10691236; doi:10.1073/pnas.2310522120)
Supplement: Supplementary file 1 — Appendix 01 (PDF) [file pnas.2310522120.sapp.pdf]

PNAS

Supporting Information for

Resistance gene-guided genome mining reveals the roseopurpurins as inhibitors of cyclin-dependent kinases (CDKs)

Kyle L Dunbar[1*], Bruno Perlatti[1*], Nicholas Liu[1*], Daniel Mummau[1], Yi-Ming Chiang[1], Amber Cornelius[1], Lawrence Hon[1], Monika Nimavat[1], Jason Pallas[1], Sina Kordes[2], Ho Leung Ng[1], Colin JB Harvey[1**]

[1]Hexagon Bio, Menlo Park, CA, 94025

[2]Proteros Biostructures GmbH Bunsenstr 7a, D-82152, Martinsried, Germany

** Corresponding Author: Colin JB Harvey

Email: colin@hexagonbio.com

This PDF file includes:

This PDF file includes: **1**
Supplementary Methods **4**
Solutions and buffers for transformation of *A. nidulans* and *A. uvarum* 4
Media Used for Roseopurpurin Production in *A. uvarum* 4
Supplementary Tables **7**
Table S1: Primers used in this study 7
Table S2: Plasmids used in this study 9
Table S3: Engineered strains used in this study 10
Table S4: Comparison of homologous clusters in *A. uvarum* and TTI001159 10
Table S5. IDs of sequences used for phylogenetic trees 11
Table S6. Data collection and processing statistics for CDK2 structure 12
Table S7. CDK2 structure refinement statistics 13
Table S8. NMR Spectroscopic data for **1** (Roseopurpurin C) 14
Table S9. NMR Spectroscopic data for **6** (4-O-demethylbarbatic acid) 15
Table S10. NMR Spectroscopic data for **7** (Hypoprotocetraric acid) 16
Table S11. NMR Spectroscopic data for **8** (3,8-dihydroxy-1,4,6,9-tetramethyl-dibenzo [b,e][1,4]dioxepin-11-one) 17
Table S12. NMR Spectroscopic data for **10** (Roseopurpurin G) 18

Table S13. NMR Spectroscopic data for **11** (Aculeatusquinone C)....................19
Table S14. NMR Spectroscopic data for **12** (Roseopurpurin D)....................20
Supplementary Figures....................21
Figure S1: Homologous BGCs containing resistance genes with homology to human CDK2 occur across multiple genomes....................21
Figure S2: Production of **1** and precursors in wild-type and engineered *A. uvarum* across cultures in multiple media....................22
Figure S3: Characterization of the biosynthesis of 1 by heterologous expression in *A. nidulans*....................23
Figure S4. BGC HX1012/HX1035-derived metabolites do not inhibit the ADP-detection assay....................24
Figure S5. Commercial depsides and depsidones are not potent inhibitors of CDK2/cyclin E1-dependent Histone H1 phosphorylation....................25
Figure S6. **1** is an ATP-competitive inhibitor of CDK2 displaying tight-binding inhibition....................26
Figure S7. Cell Cycle arrest by serum starvation and cell cycle inhibitors....................27
Figure S8. Structural comparison of **1** bound to CDK2 and other CDK2 inhibitors....................28
Figure S9. Phylogeny of kinases profiled in the selectivity panel....................29
Figure S10. Roseopurpurin C (**1**) kinase selectivity profile....................34
Figure S11. Staurosporine kinase selectivity profile....................39
Figure S12. Sequence alignment of AMPKα1 and CAMK4 with select CDKs....................40
Figure S13. Structural alignment of AMPKα1 and 1-bound CDK2....................41
Figure S14. Structural alignment of CAMK4 and 1-bound CDK2....................42
Figure S15. The ^{1}H NMR spectrum of **1** in CD_3OD (600 MHz)....................43
Figure S16. The ^{13}C NMR spectrum of **1** in CD_3OD (150 MHz)....................43
Figure S17. The ^{1}H-^{13}C HSQC NMR spectrum of **1** in CD_3OD (600 MHz)....................44
Figure S18. The ^{1}H-^{13}C HMBC NMR spectrum of **1** in CD_3OD (600 MHz)....................44
Figure S19. The ^{1}H-^{1}H NOESY NMR spectrum of **1** in CD_3OD (600 MHz)....................45
Figure S20. The ^{1}H NMR spectrum of **6** in CD_3OD (600 MHz)....................45
Figure S21. The ^{13}C NMR spectrum of **6** in CD_3OD (150 MHz)....................46
Figure S22. The ^{1}H-^{13}C HSQC NMR spectrum of **6** in CD_3OD (600 MHz)....................46
Figure S23. The ^{1}H-^{13}C HMBC NMR spectrum of **6** in CD_3OD (600 MHz)....................47
Figure S24. The ^{1}H NMR spectrum of **7** in CD_3OD (600 MHz)....................47
Figure S25. The ^{13}C NMR spectrum of **7** in CD_3OD (150 MHz)....................48
Figure S26. The ^{1}H-^{13}C HSQC NMR spectrum of **7** in CD_3OD (600 MHz)....................48
Figure S27. The ^{1}H-^{13}C HMBC NMR spectrum of **7** in CD_3OD (600 MHz)....................49
Figure S28. The ^{1}H NMR spectrum of **8** in CD_3OD (600 MHz)....................49
Figure S29. The ^{13}C NMR spectrum of **8** in CD_3OD (150 MHz)....................50
Figure S30. The ^{1}H-^{13}C HSQC NMR spectrum of **8** in CD_3OD (600 MHz)....................50
Figure S31. The ^{1}H-^{13}C HMBC NMR spectrum of **8** in CD_3OD (600 MHz)....................51
Figure S32. The ^{1}H NMR spectrum of **10** in CD_3OD (600 MHz)....................51
Figure S33. The ^{13}C NMR spectrum of **10** in CD_3OD (150 MHz)....................52
Figure S34. The ^{1}H-^{13}C HSQC NMR spectrum of **10** in CD_3OD (600 MHz)....................52
Figure S35. The ^{1}H-^{13}C HMBC NMR spectrum of **10** in CD_3OD (600 MHz)....................53
Figure S36. The ^{1}H NMR spectrum of **11** in CD_3OD (600 MHz)....................53
Figure S37. The ^{13}C NMR spectrum of **11** in CD_3OD (150 MHz)....................54
Figure S38. The ^{1}H-^{13}C HSQC NMR spectrum of **11** in CD_3OD (600 MHz)....................54
Figure S39. The ^{1}H-^{13}C HMBC NMR spectrum of **11** in CD_3OD (600 MHz)....................55

Figure S40. The ^{1}H NMR spectrum of **12** in CD_3OD (600 MHz)....................55
Figure S41. The ^{13}C NMR spectrum of **12** in CD_3OD (150 MHz).................... 56
Figure S42 The ^{1}H-^{13}C HSQC NMR spectrum of **12** in CD_3OD (600 MHz)..............56
Figure S43. The ^{1}H-^{13}C HMBC NMR spectrum of **12** in CD_3OD (600 MHz)..............57
References..57

Supplementary Methods

Solutions and buffers for transformation of *A. nidulans* and *A. uvarum*

1. **20X Nitrate Salt Solution:**
    a. Sodium nitrate (120 g/L)
    b. Potassium chloride (10.4 g/L)
    c. Magnesium sulfate heptahydrate (10.4 g/L)
    d. Potassium phosphate monobasic (30.4 g/L)
2. **Fungal 1000X Trace Elements:**
    a. EDTA (10 g/L)
    b. Zinc sulfate heptahydrate (4.4 g/L)
    c. Manganese chloride (1.01 g/L)
    d. Colbalt chloride hexahydrate (0.32 g/L)
    e. Copper sulfate pentahydrate (0.315 g/L)
    f. Ammonium molybdate pentahydrate (0.22 g/L)
    g. Calcium chloride dihydrate (1.47 g/L)
    h. Iron sulfate heptahydrate (1.0 g/L)
3. **YG Medium:**
    a. Glucose (20 g/L)
    b. Yeast extract (10 g/L)
    c. 1000X trace elements (1 mL/L)
4. **Digestion Buffer (50 mL):**
    a. Magnesium sulfate heptahydrate (14.79 g)
    b. VinoTaste Pro (6 g)
    c. Yatalase (75 mg)
    d. 175 mM sodium phosphate buffer (up to 50 mL)
5. **0.4 M ST Buffer:**
    a. Sorbitol (72.86 g/L)
    b. 1 M Tris-HCl, pH 8.0 (20 mL/L)
6. **0.6 M KCl Solution:**
    a. Potassium chloride (44.7 g/L)
7. **Transformation Buffer: 0.6 M KCl, 50 mM $CaCl_2$ Solution:**
    a. Potassium chloride (44.7 g/L)
    b. Calcium chloride (7.4 g/L)
8. **PEG Solution (40 mL)**
    a. 24 g PEG 3,350 (60% (w/v))
    b. 0.29 g $CaCl_2$ heptahydrate / or 2 mL of 1 M stock solution
    c. 2 mL Tris-HCl pH 7.5 (2 mL from 1 M stock)
        i. Use a 50 mL sterile tube. Start with 2 mL Tris, add PEG and $CaCl_2$ and fill up with RO water (needs very little). Shake to make sure PEG and $CaCl_2$ is suspended in water. Put in 55°C water bath to dissolve and bring up to 40 mL as needed. Do not place the tube on ice as the PEG and C\a$Cl_2$ will crash out of solution.
9. **Sorbitol Minimal Media (SMM):**
    a. D-Glucose (10 g/L)
    b. 20X nitrate salts (50 mL/L)
    c. Sorbitol (218.6 g/L)
    d. 1000X trace elements (1 mL/L)

Media Used for Roseopurpurin Production in *A. uvarum*

1. PD

    a. 40 g/L Potato Dextrose Broth
2. MME
    a. 30g/L Malt Extract
    b. 3g/L peptone
    c. 0.01g/L $ZnSO4·7H2O$
    d. 0.005g/L $CuSO4·5H2O$
3. CYS80
    a. 80g/L sucrose
    b. 50g/L yellow cornmeal
    c. 1g/L yeast extract
4. WATM
    a. 2g/L NaNO3
    b. 30g/L saccharose
    c. 2g/L yeast extract
    d. 3g/L peptone
    e. 5g/L corn steep solids
    f. 1g/L $KH2PO4·H20$
    g. 0.2g/L KCl
    h. 0.5g/L $FeSO4·7H2O$
    i. 0.01g/L $ZnSO4·7H2O$
    j. 0.005g/L $CuSO4·5H2O$
5. Supermalt
    a. 50g/L Malt Extract
    b. 10g/L Yeast Extract
    c. 20 mg/L FeSO4.7H2O
    d. 7 mg/L ZnSO4.7H2O
6. MGTY
    a. 15 g/L Maltose
    b. 10 g/L Glycerol
    c. 10 g/L Tryptone
    d. 10 g/L Yeast extract
    e. 1 g/L KH_2PO_4
    f. 0.2 g/L $MgSO_4$
    g. 0.5 g/L $CaCl_2$
7. SMK
    a. 40 g/L Soluble Starch
    b. 1 g/L Yeast Extract
    c. 4.3 g/L Murashige & Skoog Salts
8. MMSY
    a. 40 g/L Mannitol
    b. 5 g/L Yeast Extract
    c. 4.3 g/L Murashige & Skoog Salts
    d.
9. MEY
    a. 20g/L Malt Extract
    b. 5g/L Yeast extract
10. GLX
    a. 10g/L peptone
    b. 21g/L malt extract

c. 40g/L glycerol

11. CYA
    a. 3g/L $NaNO_3$
    b. 5g/L yeast extract
    c. 30g/L sucrose
    d. 1.3g/L K_2HPO_4
    e. 10ml/L Czapek concentrate (10 mL/L)
12. YPSS
    a. 4g/L yeast extract
    b. 14g/L soluble starch
    c. 1g/L K_2HPO_4
    d. 0.5 g/L $MgSO_4 \cdot 7H_2O$

Supplementary Tables

Table S1: Primers used in this study

Primer Name	Primer Sequence	Plasmids Used In
Ba3uU0j0w5bi	CTTTCAACCATACATATTCCCCGTTC	pNL0078
0gCudLDxE4iS	CTCCCTCTTTGCCAGTTCTCC	pNL0075<br>pNL0076<br>pNL0077<br>pNL0078
PDELrFSyuB0C	CTTGAACGAGAGGCGTCAGCGAC	pNL0075<br>pNL0076<br>pNL0077<br>pNL0078
td7CbYPTeKut	CTGAGCACTTCTCCCTTTTATATTCCACAAAACATAACACAACCTTCACCATGGTGGGCACAATTGACACACACC	pNL0078
U8q1J1doZSPB	GCACCAAACAGGACAGCTGATCAAGG	pNL0075
IH1lLXduJlGh	ATGGATGGAGTGAACACTAC	pNL0078
YGX4kAOVe4Bz	TTTTATAAATTAGCCCTTCATG	pNL0078
8m5Hut2DMdSs	GGCAGACTTCTCTGCG	pNL0078
SNImelv5Avtf	ATCCTATTCTTTCATCGTAC	pNL0076<br>pNL0077<br>pNL0078
U8q1J1doZSPB	GCACCAAACAGGACAGCTGATCAAGG	pNL0076<br>pNL0077<br>pNL0078
BFck2StgbGZj	CTCACGAAGCTTGACTAACCATTACCCCGCCACATAGACACATCTAAACAATGGATACCCTCACCAGGCCCAGA	pNL0078
BFck2StgbGZj	CTCACGAAGCTTGACTAACCATTACCCCGCCACATAGACACATCTAAACAATGGATACCCTCACCAGGCCCAGA	pNL0077
wjWoGo3FGbFT	ATCACCGCTAGAACGTCTATCTCATCACCGACTTCTCATCCATCTTCAAAATGCAGGACGAAGTCAACCAATACGTAGA	pNL0078
upKMOhU4YeUT	CCATCATTTTCTAGAGAAACTCCTCTCTCAGAACCACCACAAACCATCACATGTACTGTGCTGAAACCACAAACAGTCAGACCACGTTTAGCGACGCCTTCCGGAGCAGGTCGTGGCCAGAGCGCTCG	pNL0076<br>pNL0077<br>pNL0078
tQe7cSidLbG6	AAAAGCCATGCCTTCGTGAT	pNL0077<br>pNL0078
CNSA15FuTAQY	AAGCATTTCAGCCCTTTTCTATGGGACAAAGG	pNL0077<br>pNL0078
XfMEmkpDDwcf	AAATGGGATTGTCTTGGTCAATCAAG	pNL0078

eGyscqDZ04ul	TTGCTTCGAAGACCTCATAGAATTTATGTCGCT	pNL0075<br>pNL0076<br>pNL0077<br>pNL0078
F7QLyNHITmSz	GCCACAGCTTGATGTAGCTTAGACCCT	pNL0075<br>pNL0076<br>pNL0077<br>pNL0078
GcGhrSiIH3uE	TAGGATATTTGCCAATGTCAAAGACTTCCGGTAGTGTTCACTCCATCCATCTATCCATACTGAGAAAGTCGAGGGAACAGC	pNL0078
dN2hEszOWCod	ATAATGAACCTCCAAGATGTATCCTAAGTCGGTAACCTCATTCTCGGCACCTACAAATGATCGAACAGAAATTTATAACCCGGAGCA	pNL0075
2qRtP7v2KLxc	GTTGGGCAGTTATTTGCAATGA	pNL0078
JWW4zkZUMH9T	GGTGAAGGTTGTGTTATGTT	pNL0078
of4jnKXZfyCv	TTTGAAGATGGATGAGAAGTCG	pNL0078
W44RR81Xl4Rj	TGTGATGGTTTGTGGTGGTTCTGAG	pNL0076<br>pNL0077<br>pNL0078
gV8gwo6LEMES	TTTTAGGTATATTTTTGGACGTGGATCAC	pNL0076<br>pNL0077<br>pNL0078
3FnizHFBw2tm	ACCCAACACGCATCACGTCCAATCCGCACATGAAGGGCTAATTTATAAAATTACAGCCGGCGTTTGAGATAAACCCT	pNL0078
PG89RXoqsKn2	ATAATGAACCTCCAAGATGTATCCTAAGTCGGTAACCTCATTCTCGGCACTTACAGCCGGCGTTTGAGATAAACCCT	pNL0077
KV3mrYD8bFTx	ATAATGAACCTCCAAGATGTATCCTAAGTCGGTAACCTCATTCTCGGCACTCATTCCTTGATTGTGACCTCGAGAAGGC	pNL0078
bGzWwY1UA4GK	CTTGGACCCGATGCAATTCCTTTGTCC	pNL0076<br>pNL0077<br>pNL0078
tWYOXTqwoABz	TGTTTAGATGTGTCTATGTG	pNL0077<br>pNL0078
D9dyHRY2ejYY	TTCACCTAGTGGATTTCCTAGCATAACATC	pNL0077<br>pNL0078
hqWDueMahIut	ATAATGAACCTCCAAGATGTATCCTAAGTC	pNL0076
DY092 1035 coxAp amidohydrolase F	GTTCTCCACGCCTTGTCCGTTGGCATTGCACCCACAATGGTGGGCACAATTGACACACAC	pNL0079
DY093 1035 amidohydrolase tAN717 R	ATCCTAAGTCGGTAACCTCATTCTCGGCACCTATCCATACTGAGAAAGTCGAGGGAACAG	pNL0079
HX1035-SDR-fix-F	CACGCCTTGTCCGTTGGCATTGCACCCACAATGTATTCATTTCAAGGCCACCAGGACC	pNL0080

HX1035-SDR-fix-R	CTAAGTCGGTAACCTCATTCTCGGCACTCATTGATTATCACTAACACGTACATAGTGCCC	pNL0080
coxAp 1012 glyoxalase	AGTTCTCCACGCCTTGTCCGTTGGCATTGCACCCACAATGCACCCACTGAATAACACCCC	pNL0082
1012 glyoxalase tAN0717 R	CCTCCAAGATGTATCCTAAGTCGGTAACCTCATTCTCGGCACTTAGGGCTCGCTCGGGGG	pNL0082
HX1021-HP-F-coxA	CACGCCTTGTCCGTTGGCATTGCACCCACAATGCCCTCGGGATATTGTCTCTTTTCTC	pNL0081
HX1021-HP-R-tAN0717	ATCCTAAGTCGGTAACCTCATTCTCGGCACTTAATCAGAATTACACGTCGCGAGGG	pNL0081

Table S2: Plasmids used in this study

Plasmid	Genes Expressed	Backbone Plasmid
pNL0075	*rosA*	pHex317
pNL0076	*rosAB*	pHex317
pNL0077	*rosABC*	pHex317
pNL0078	*rosABCDEH*	pHex317
pNL0079	*rosD*	pHex318
pNL0080	*rosE*	pHex344
pNL0081	Aspuva1_93046	pHex344
pNL0082	*rosG*	pHex318
pNL0083	empty plasmid	pHex344
pNL0084	empty plasmid	pHex317
pNL0085	Aspuva1_365195	pHex385
pNL0086	empty plasmid	pHex385

Table S3: Engineered strains used in this study

Strains	Description	Plasmids contained	Parent Strain
AN0045	*A. nidulans expressing prosJKB*	pNL0076+pNL0079+pNL0083	bghX17
AN0046	*A. nidulans expressing prosJKAB*	pNL0077+pNL0079+pNL0083	bghX17
AN0047	*A. nidulans expressing prosJKBC*	pNL0076+pNL0079+pNL0080	bghX17
AN0048	*A. nidulans expressing prosJKABC*	pNL0077+pNL0079+pNL0080	bghX17
AN0049	*A. nidulans expressing prosJKAB+Aspuva1_93046*	pNL0077+pNL0079+pNL0081	bghX17
AN0050	*A. nidulans expressing prosJKABCGDF*	pNL0078+pNL0082+pNL0083	bghX17
AN0051	*A. nidulans expressing prosJKABCGDF+Aspuva1_93046*	pNL0078+pNL0082+pNL0081	bghX17
AN0052	*A. nidulans expressing prosJKABCGF+Aspuva1_93046*	pNL0078+pNL0084+pNL0081	bghX17
AN0053	*A. nidulans expressing prosJ*	pNL0075	bghX18
AN0054	*A. nidulans expressing prosJK*	pNL0076	bghX18
AN0055	*A. nidulans expressing prosJKA*	pNL0077	bghX18
AN0056	*A. nidulans expressing prosJKABCF*	pNL0078	bghX18
AU0001	*A. uvarum with empty pHex385 plasmid*	pNL0086	*A. uvarum*
AU0002	*A. uvarum expressing rosH*	pNL0085	*A. uvarum*
AU0003	*A. uvarum with KO of rosJ*		*A. uvarum*

Table S4: Comparison of homologous clusters in *A. uvarum* and TTI001159

TTI001159	*A. uvarum*	Putative Function	% Identity
prosJ	*rosJ*	NR-PKS(SAT-KS-AT-PT-ACP-ACP-MT-TE)	50.94%
prosK	*rosK*	P450	62.73%
prosA	*rosA*	P450	70.29%
prosB	*rosB*	Amidohydrolase	75.08%
prosC	*rosC*	Reductase	64.67%
prosG	*rosG*	CDK2	65.41%
prosD	*rosD*	Glyoxylase	53.89%
prosF	*rosF*	Methyltransferase	74.50%
prosH	*rosH*	Transcription FActor	25.58%
prosI	*rosI*	Transporter	63.72%
-	*rosE*	Hypothetical Protein	

Table S5. IDs of sequences used for phylogenetic trees.

Protein	Uniprot ID		Protein	Uniprot ID
CDK1	P06493		CLK3	P49761
CDK2	P24941		Aurora A	O14965
CDK3	Q00526		CK2α1	P68400
CDK4	P11802		IKKβ	O14920
CDK5	Q00535		CK1α1	P48729
CDK6	Q00534		CK1γ1	Q9HCP0
CDK7	P50613		PKCα	P17252
CDK8	P49336		ROCK1	Q13464
CDK9	P50750		AKT1	P31749
CDK10	Q15131		FGFR1	P11362
CDK11A	Q9UQ88		JAK3	P52333
CDK11B	P21127		LCK	P06239
CDK12	Q9NYV4		SYK	P43405
CDK13	Q14004		MINK1	Q8N4C8
CDK14	O94921		PAK1	Q13153
CDK15	Q96Q40		IRAK4	Q9NWZ3
CDK16	Q00536		TAK1	O43318
CDK17	Q00537		GSK3β	P49841
CDK18	Q07002		p38α	Q16539
CDK19	Q9BWU1		AMPK α1	Q13131
CDK20	Q8IZL9		CAMK4	Q16566
GSK3a	P49840		CHK1	O14757
A. uvarum RosG	XP_025491502.1		DAPK1	P53355
			MAPKAPK2	P49137

Table S6. Data collection and processing statistics for CDK2 structure

	**Roseopurpurin C (1)**
X-ray source	PXII/X10SA (SLS[1])
Wavelength [Å]	0.9999
Detector	Dectris EIGER2 Si 16M
Temperature [K]	100
Space group	P 2_1 2_1 2_1
Cell: a; b; c; [Å]	54.36; 72.69; 72.96
α; β; γ; [°]	90.0; 90.0; 90.0
Resolution [Å]	2.62 (2.66-2.62)
Unique reflections	8294 (316)
Multiplicity	4.3 (2.1)
Completeness [%]	90.6 (70.7)
R_{pim} [%][5]	5.6 (43.7)
R_{sym} [%][3]	11.5 (59.4)
R_{meas} [%][4]	12.9 (74.2)
CC1/2 [%]	99.50 (73-90)
Mean(I)/sd[6]	8.8 (1.3)

[1] SWISS LIGHT SOURCE (SLS, Villigen, Switzerland)

[2] values in parenthesis refer to the highest resolution bin.

[3] $$Rsym = \frac{\sum_h \sum_i^{n_h} \left| \hat{I}_h - I_{h,i} \right|}{\sum_h \sum_i^{n_h} I_{h,i}} \quad \text{with} \quad \hat{I}_h = \frac{1}{n_h} \sum_i^{n_h} I_{h,i}$$

where $I_{h,i}$ is the intensity value of the ith measurement of h

Table S7. CDK2 structure refinement statistics

Ligand	Roseopurpurin C (1)
Resolution [Å]	51.49-2.62
Number of reflections (working /test)	7872 / 417
R_{cryst} [%]	22.0
R_{free}[%]	28.3
Total number of atoms:	
Protein	2211
Water	39
Ligand	25
Glycerol	6
Deviation from ideal geometry: [2]	
Bond lengths [Å]	0.005
Bond angles [°]	1.51
Bonded B's [$Å^2$] [3]	0.5
Ramachandran plot: [4]	
Most favoured regions [%]	93.66
Additional allowed regions [%]	5.97
Generously allowed regions [%]	1.35
Disallowed regions [%]	0.88

[1] Values as defined in REFMAC5, without sigma cut-off
[2] Root mean square deviations from geometric target values
[3] Calculated with MOLEMAN
[4] Calculated with PROCHECK

Table S8. NMR Spectroscopic data for 1 (Roseopurpurin C)

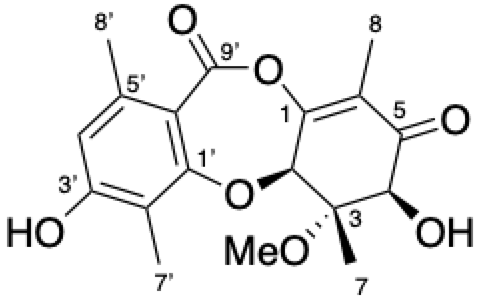

	Found		Literature[1]	
Position	δ_C	δ_H, m(J in Hz)	δ_C	δ_H, m(J in Hz)
1	162.8		162.7	
2	82.1	5.24, q (2.0)	82	5.24, d (2.0)
3	84.8		84.9	
4	76.5	4.41, s	76.4	4.4, s
5	198.9		198.9	
6	118.5		118.7	
7	13.4	1.14, s	13.3	1.14, s
8	8.6	1.88, d (2.0)	8.6	1.88, d (2.0)
3-OMe	51.1	3.41, s	51.1	3.41, s
1'	161.1		161.2	
2'	115.8		115.5	
3'	164.2		162.5	
4'	115.9	6.58, s	115.7	6.59, s
5'	144.4		144.5	
6'	112.5		112.5	
7'	8.6	2.18, s	8.6	2.18, s
8'	22.3	2.45, s	22.3	2.45, s
9'	162.7		162.9	

In CD_3OD, 600 MHz for ^1H and 150 MHz for ^{13}C NMR; Chemical shifts are reported in ppm. All signals are determined by ^1H, ^{13}C, HSQC and HMBC correlation.

HRMS $(M+H)^+$ *m/z* calculated for $(C_{18}H_{20}O_7+H)^+$ 349.1282; found 349.1274.

Table S9. NMR Spectroscopic data for 6 (4-O-demethylbarbatic acid)

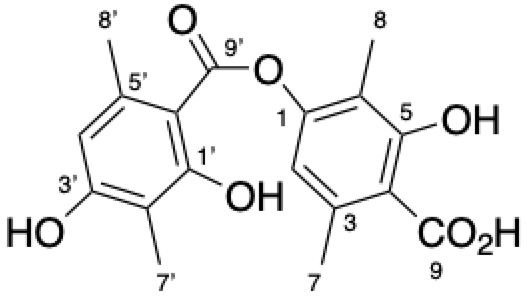

	Found (CD_3OD)		Reported (Acetone-d6) [2]	
Position	δ_C	δ_H, m(J in Hz)	δ_C	δ_H, m(J in Hz)
1	153.7		153	
2	117.5	6.54, s	116.7	6.72, s
3	141.6		140.9	
4	111.3		109.6	
5	164.3		164	
6	117.6		117.5	
7	24	2.59, s	23	2.68, s
8	9.4	2.02, s	7.3	2.04, s
9	175.1		170.4	
1'	165.5		163.3	
2'	110.5		109.2	
3'	162.5		160.5	
4'	112.2	6.33, s	111.3	6.47, s
5'	141.4		140.7	
6'	104		103.6	
7'	9.5	2.03, s	8.7	2.05, s
8'	24.7	2.58, s	23.7	2.63, s
9'	171.5		170.4	

In CD_3OD, 600 MHz for ^1H and 150 MHz for ^{13}C NMR; Chemical shifts are reported in ppm. All signals are determined by ^1H, ^{13}C, HSQC and HMBC correlation.

HRMS $(M+H)^+$ *m/z* calculated for $(C_{18}H_{18}O_7+H)^+$ 347.1125; found 347.1117.

Table S10. NMR Spectroscopic data for 7 (Hypoprotocetraric acid)

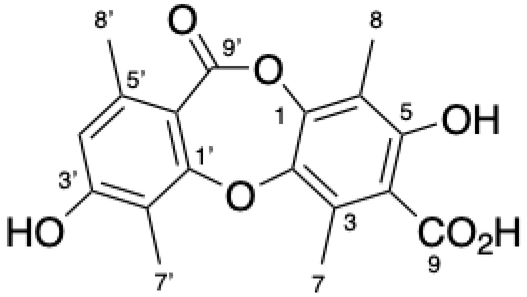

	Found (CD3OD)		Literature (CDCl3, DMSO-*d*6) [3]	
Position	δ_C	δ_H, m(J in Hz)	δ_C	δ_H, m(J in Hz)
1	148.5			
2	144.2			
3	131.1			
4	111.3			
5	160.1			
6	116.5			
7	18.3	2.70, s	2.66, s	
8	9.1	2.19, s	2.17, s	
9	174.8			
1'	162.8			
2'	114.5			
3'	162			
4'	115.5	6.56, s	6.59, s	
5'	143.1			
6'	113.2			
7'	10.3	2.31, s	2.28, s	
8'	21	2.36, s	2.35, s	
9'	165			

In CD_3OD, 600 MHz for ^1H and 150 MHz for ^{13}C NMR; Chemical shifts are reported in ppm. All signals are determined by ^1H, ^{13}C, HSQC and HMBC correlation.

HRMS $(M+H)^+$ *m/z* calculated for $(C_{18}H_{16}O_7+H)^+$ 345.0969; found 345.0974.

Table S11. NMR Spectroscopic data for 8 (3,8-dihydroxy-1,4,6,9-tetramethyl-dibenzo [b,e][1,4]dioxepin-11-one)

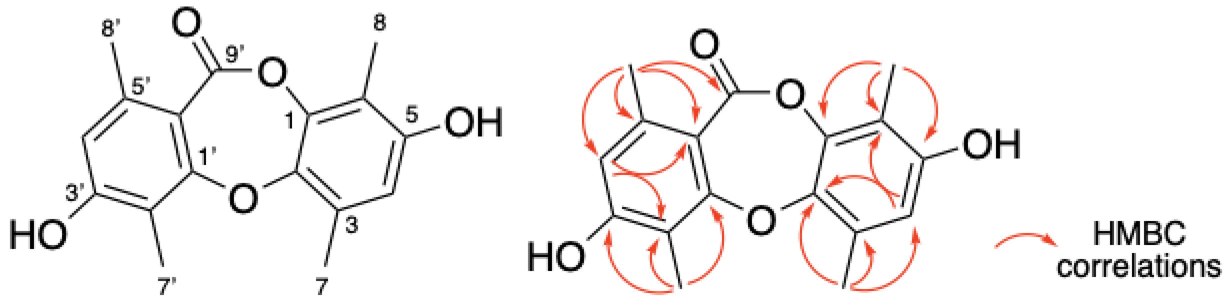

	8	
Position	δ_C	δ_H, m(J in Hz)
1	145	
2	144.4	
3	128.1	
4	114	6.43, s
5	153.8	
6	115.4	
7	17.8	2.35, s
8	9.3	2.12, s
1'	163.2	
2'	114.8	
3'	161.6	
4'	115.3	6.56, s
5'	143.1	
6'	112.5	
7'	9.9	2.29, s
8'	21.3	2.36, s
9'	166.5	

In CD_3OD, 600 MHz for ^1H and 150 MHz for ^{13}C NMR; Chemical shifts are reported in ppm. All signals are determined by ^1H, ^{13}C, HSQC and HMBC correlation.

HRMS $(M+H)^+$ *m/z* calculated for $(C_{17}H_{16}O_5+H)^+$ 301.1070; found 301.1062.

Table S12. NMR Spectroscopic data for 10 (Roseopurpurin G)

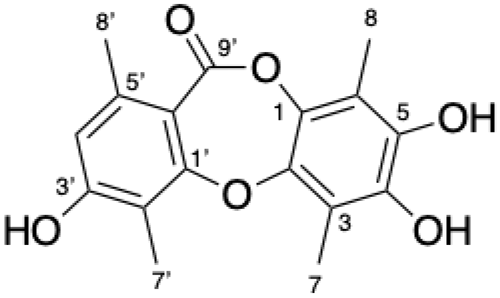

	Found		Literature [1]	
Position	δ_C	δ_H, m(J in Hz)	δ_C	δ_H, m(J in Hz)
1	137.6		137.6	
2	142.1		142	
3	115.6		115.6	
4	144.7		144.7	
5	142		142.1	
6	115.3		115.2	
7	10.8	2.31, s	10.7	2.31, s
8	9.6	2.17, s	9.6	2.18, s
1'	163.6		163.5	
2'	114.3		114.3	
3'	161.5		161.3	
4'	115.2	6.53, s	115.2	6.53, s
5'	143		142.9	
6'	113.6		113.6	
7'	10.0	2.30, s	10	2.30, s
8'	21.4	2.35, s	21.3	2.35, s
9'	167.7		167.1	

In CD_3OD, 600 MHz for ^1H and 150 MHz for ^{13}C NMR; Chemical shifts are reported in ppm. All signals are determined by 1H, 13C, HSQC and HMBC correlation.

HRMS $(M+H)^+$ *m/z* calculated for $(C_{17}H_{16}O_6+H)^+$ 317.1020; found 317.1010.

Table S13. NMR Spectroscopic data for 11 (Aculeatusquinone C)

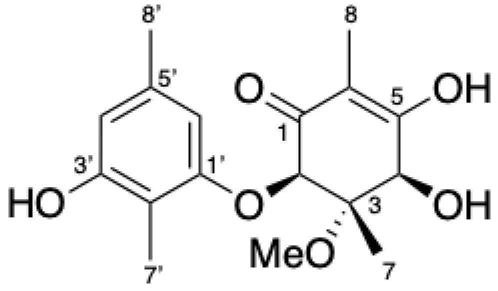

	Found		Literature [1]	
Position	δ_C	δ_H, m(J in Hz)	δ_C	δ_H, m(J in Hz)
1	190.3[a]		nd	
2	82.3	5.07, s	82.1	5.07, br s
3	84.6		84.5	
4	72.2	4.67, s	72.1	4.67, br s
5	178.7[a]		nd	
6	109.4		109.4	
7	13.0	1.23, s	13.0	1.24, s
8	7.7	1.73, s	7.7	1.73, s
3-OMe	51.1	3.20, s	51.1	3.21, s
1'	159.7		159.6	
2'	111.8		111.9	
3'	156.8		156.9	
4'	110.5	6.28, s	110.3	6.28, s
5'	136.9		137.0	
6'	107.3	6.29, s	107.1	6.30, br s
7'	8.7	2.1, s	8.7	2.1, s
8'	21.6	2.19, s	21.6	2.19, s

In CD_3OD, 600 MHz for ^1H and 150 MHz for ^{13}C NMR; Chemical shifts are reported in ppm. All signals are determined by ^1H, ^{13}C, HSQC and HMBC correlation.
[a] Determined by HMBC correlations

HRMS $(M+H)^+$ *m/z* calculated for $(C_{17}H_{21}O_6+H)^+$ 323.1489; found 323.1476.

Table S14. NMR Spectroscopic data for 12 (Roseopurpurin D)

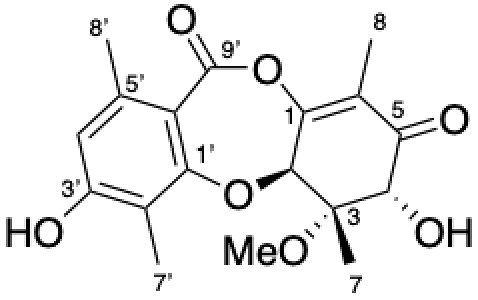

	Found		Literature [1]	
Position	δ_C	δ_H, m(J in Hz)	δ_C	δ_H, m(J in Hz)
1	157.1		157.1	
2	18.7	4.99, s	78.7	4.98, s
3	81.6		81.5	
4	77.3	4.39, s	77.3	4.39, s
5	198.3		198.3	
6	119.3		119.3	
7	16.5	1.68, s	16.4	1.67, s
8	7.9	1.75, s	7.9	1.76, s
3-OMe	52.1	3.33, s	52.1	3.34, s
1'	161.0		161.0	
2'	112.8		112.8	
3'	157.4		157.4	
4'	114.0	6.49, s	113.9	6.49, s
5'	141.7		141.7	
6'	109.9		109.8	
7'	9.0	2.07, s	8.9	2.08, s
8'	22.8	2.37, s	22.7	2.37, s
9'	164.2		164.2	

In CD_3OD, 600 MHz for ^1H and 150 MHz for ^{13}C NMR; Chemical shifts are reported in ppm. All signals are determined by ^1H, ^{13}C, HSQC and HMBC correlation.

HRMS $(M+H)^+$ *m/z* calculated for $(C_{18}H_{20}O_7+H)^+$ 349.1282; found 349.1267.

Supplementary Figures

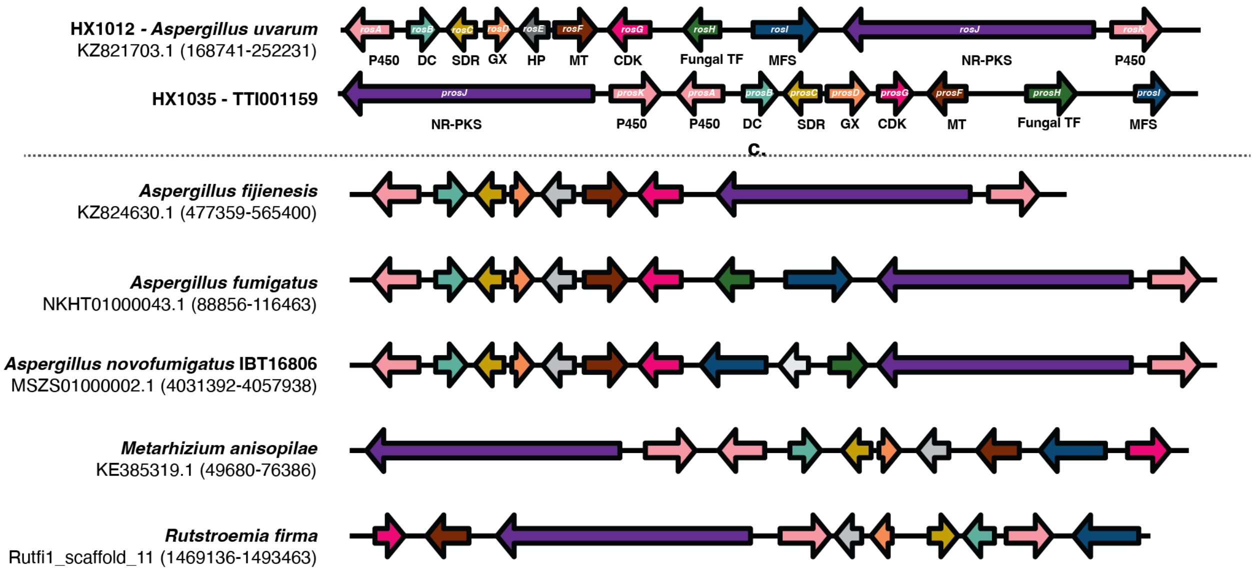

Figure S1: Homologous BGCs containing resistance genes with homology to human CDK2 occur across multiple genomes. Nucleotide sequence and coordinates (either NCBI or JGI ids) are denoted beneath the organism name**.**

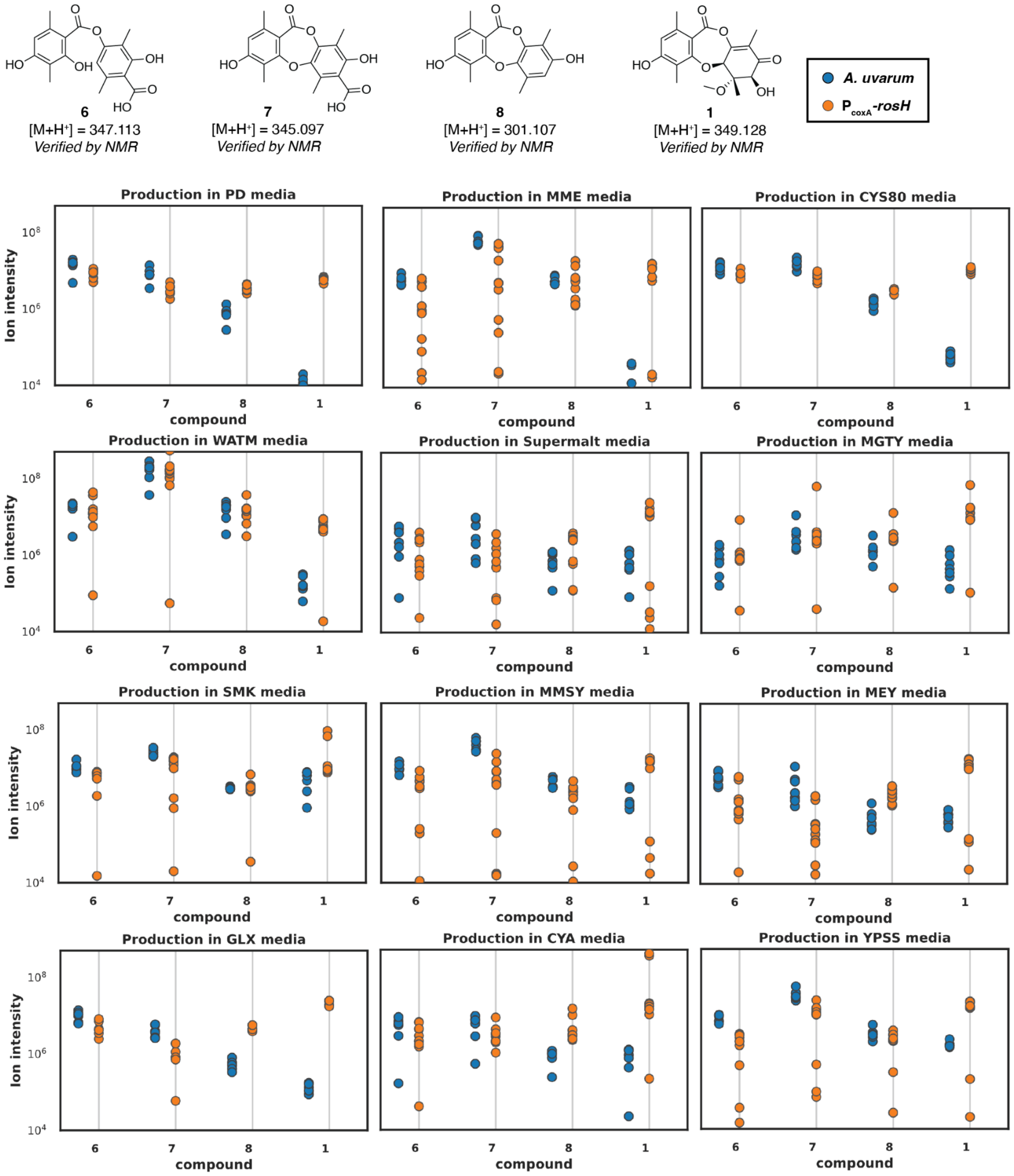

Figure S2: Production of 1 and precursors in wild-type and engineered *A. uvarum* across cultures in multiple media. All ion intensities represent the maximum intensity of the [M+H$^+$] ion for each compound. All measurements were taken on 6 biological replicates (n=6).

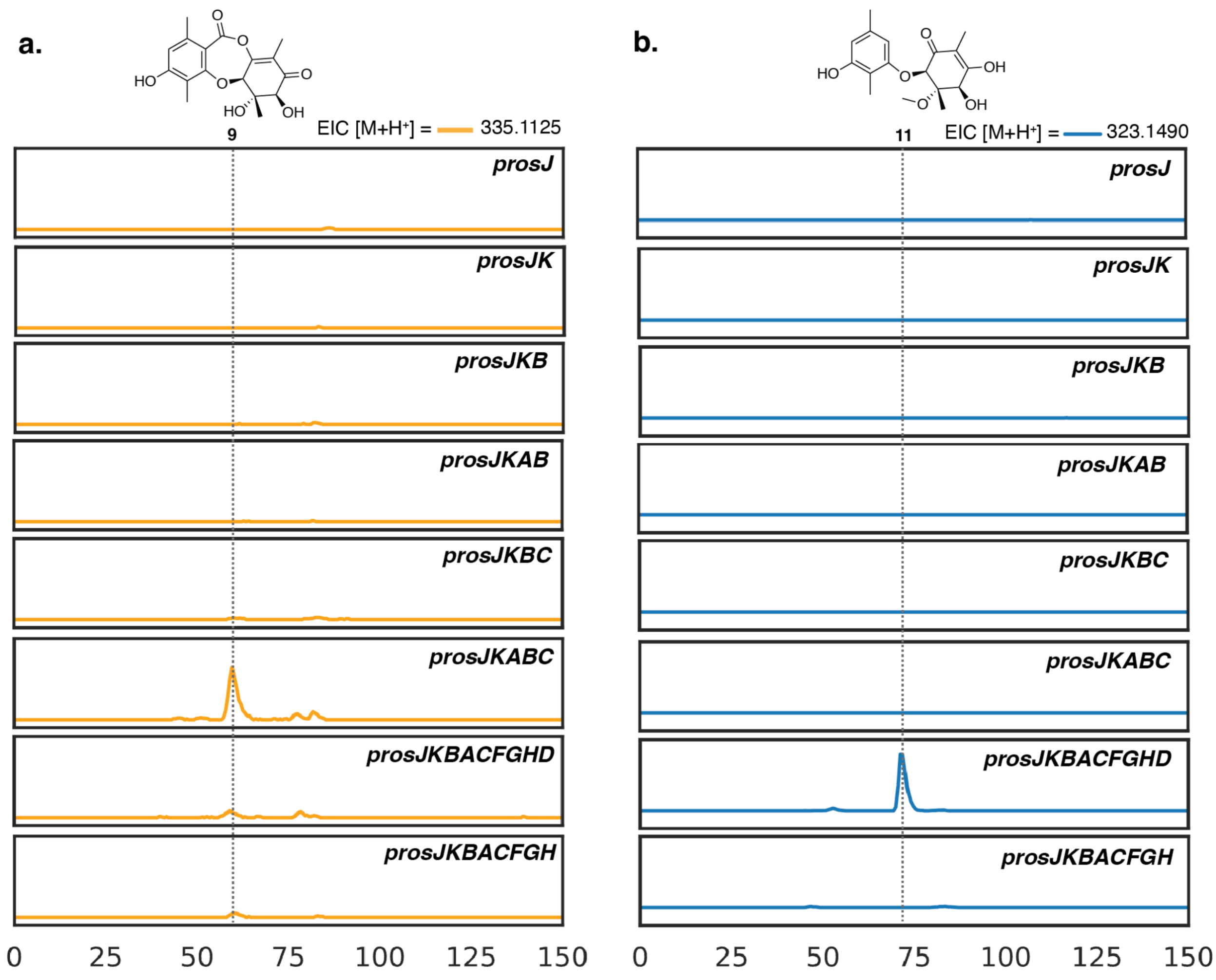

Figure S3: Characterization of the biosynthesis of 1 by heterologous expression in *A. nidulans*. a. Intermediate **9** is produced with the concerted expression of the core NR-PKS (*prosJ*), cytochrome p450s (*prosA, prosK*), the decarboxylase (*prosB*), and the reductase (*prosC*). **b**. Compound **11** is produced only in the presence of glyoxalase *prosD*. EIC = extracted ion chromatogram.

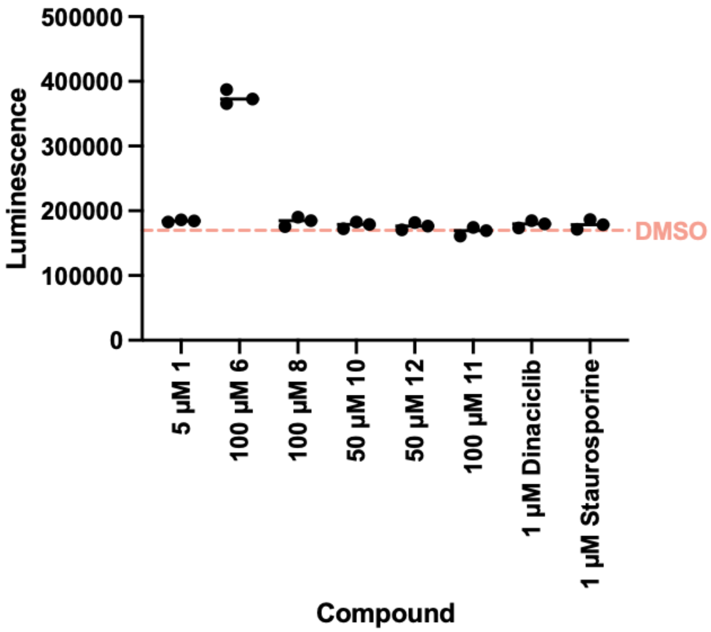

Figure S4. BGC HX1012/HX1035-derived metabolites do not inhibit the ADP-detection assay. Metabolites **1**, **6**, **8**, **10**, **11**, and **12** were tested for interference of ADP detection in the ADP-Glo (Promega) assay. Assays were performed by replacing CDK2/cyclin E1 with 10 µM ADP. No metabolite inhibited the detection of ADP by the ADP-Glo assay; however, **6** showed a 2-fold increase in luminescence signal relative to the DMSO control.

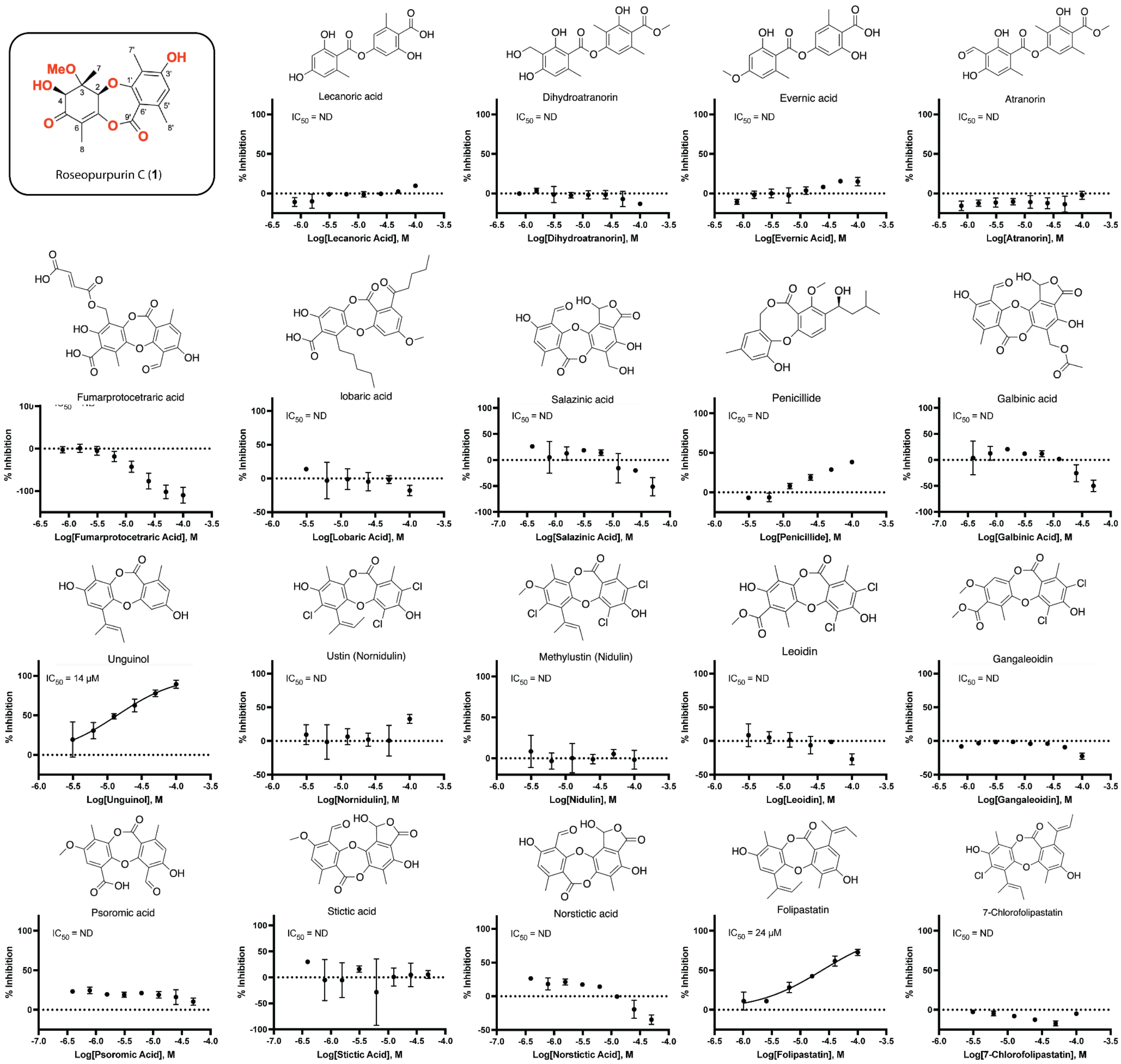

Figure S5. Commercial depsides and depsidones are not potent inhibitors of CDK2/cyclin E1-dependent Histone H1 phosphorylation. 19 commercially available depsides and depsidones were assayed for inhibition of CDK2/cyclin E1-dependent phosphorylation of Histone H1 using the luciferase-dependent ADP-detection assay. Data represent the average ± the standard deviation of the mean, n=2. Inhibition >50% was only observed for unguinol (IC_{50} = 14 µM) and folipastatin (IC_{50} = 24 µM). Fumarprotocetriaric acid treatment increased luminescence values rather than suppressing them. ND = not determined; inhibition did not exceed 50% at the highest concentration tested (100 µM). The structure of **1** is shown for reference.

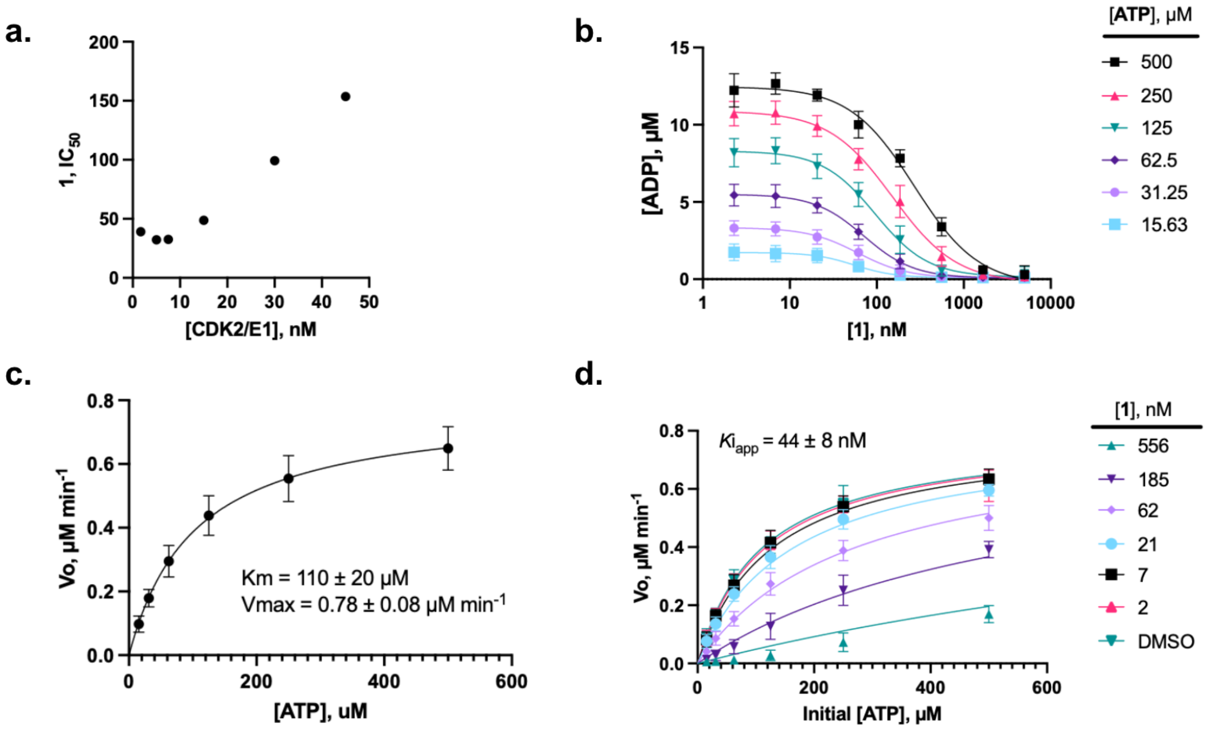

Figure S6. 1 is an ATP-competitive inhibitor of CDK2 displaying tight-binding inhibition, a) 1 displays tight binding inhibition of cyclin E1-activated CDK2 phosphorylation of histone H1. The IC_{50} of **1** was determined at variable concentrations of CDK2/cyclin E1. Tight binding inhibition was observed at enzyme concentrations greater than 7.5 nM. **b)** The IC_{50} of **1** is dependent on the concentration of ATP. Data represent the average ± standard deviation, n=4. Dose response curves were fitted in PRIMS 9 using a 4-parameter inhibitor fit equation. IC_{50} were plotted in **Figure 3c**. **c)** CDK2/cyclin E1 Michaelis-Menten curve for ATP performed with histone H1 peptide. Data represent the average ± standard deviation, n=13. Data were fit using the Michaelis-Menten equation using PRISM 9. Km and Vmax values were calculated for each replicate and are reported as the mean ± standard deviation (n=13). **d**) Michaelis-Menten curve for ATP with variable concentrations of **1** performed with histone H1 peptide. Data represent the mean ± standard deviation, n=4. Curves were fit to a competitive inhibition model in PRISM 9. The K_{iapp} is the average ± standard deviation obtained for the fits from four independent experiments. These data were replotted to obtain the Lineweaver-Burk plots in **Figure 3d.**

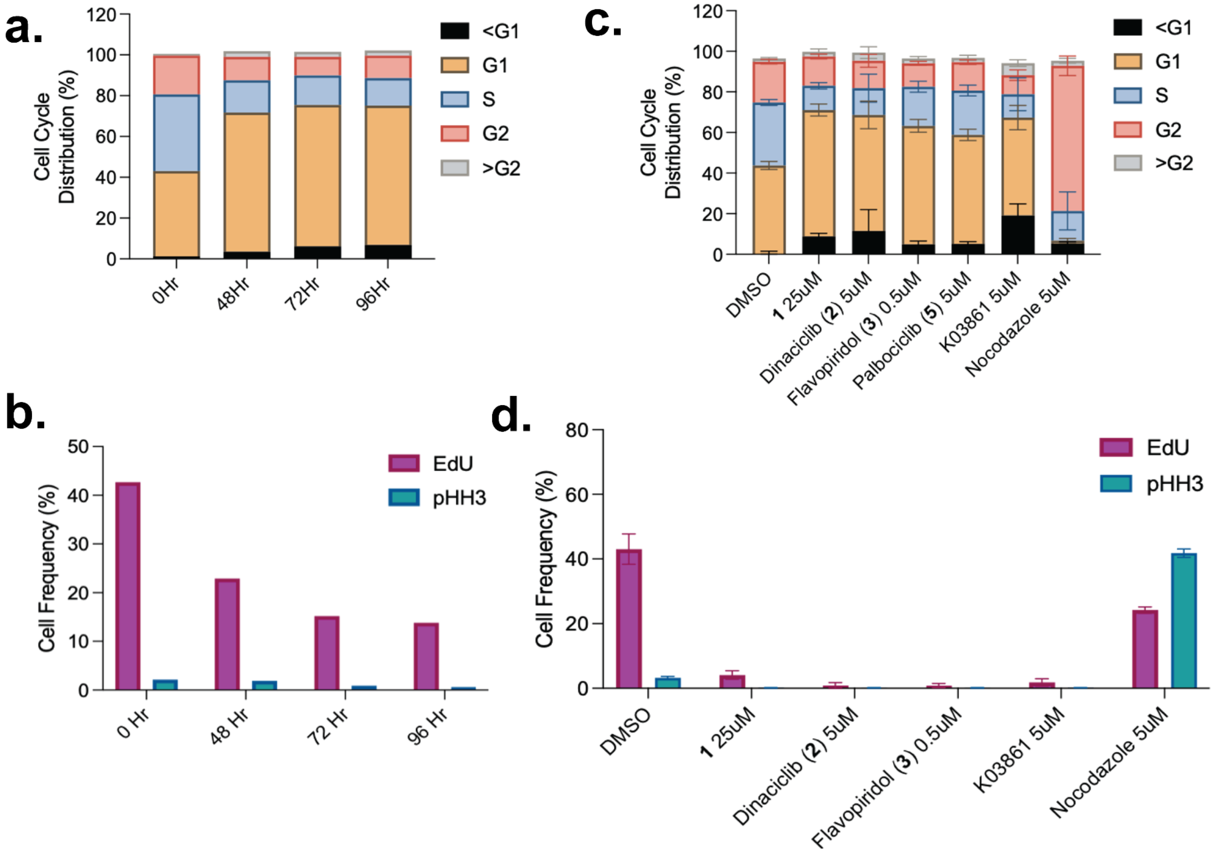

Figure S7. Cell Cycle arrest by serum starvation and cell cycle inhibitors. a) Cell cycle distribution after 48, 72, and 96 hours of serum starvation. **b)** EdU and pHH3 incorporation measured by flow cytometry after 48, 72, and 96 hours of serum starvation. **c)** Cell cycle distribution after 96 hours of serum starvation and 24-hour treatment with cell cycle inhibitory compounds. **d)** EdU incorporation and pHH3 levels measured by flow cytometry after 96 hours of serum starvation and 24-hour treatment with cell cycle inhibitory compounds.

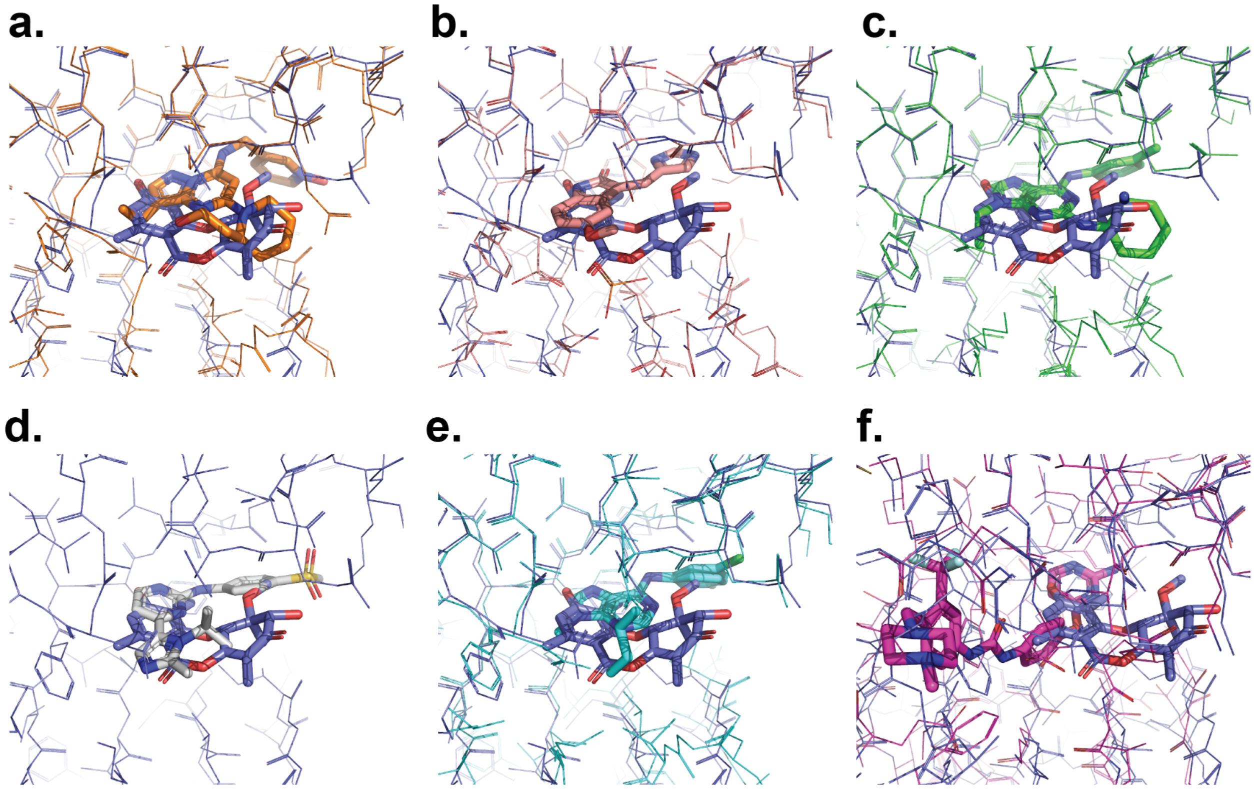

Figure S8. Structural comparison of 1 bound to CDK2 and other CDK2 inhibitors. Overlays of **1**-bound (Blue; PDB: 8OY2) and **(a)** dinaciclib-bound (orange; PDB: 4KD1), **(b)** SU9516-bound (pink; PDB: 3PY0); **(c)** CGP74514A-bound (green; PDB: 6GUK), **(d)** AZA-5438-bound (gray; PDB: 6GUH), **(e)** purvalanol B-bound (cyan; 1CKP), and **(f)** K03861-bound (pink; PDB: 5A14). Structural alignments were made using TM-align. (Zhang and Skolnick 2005).

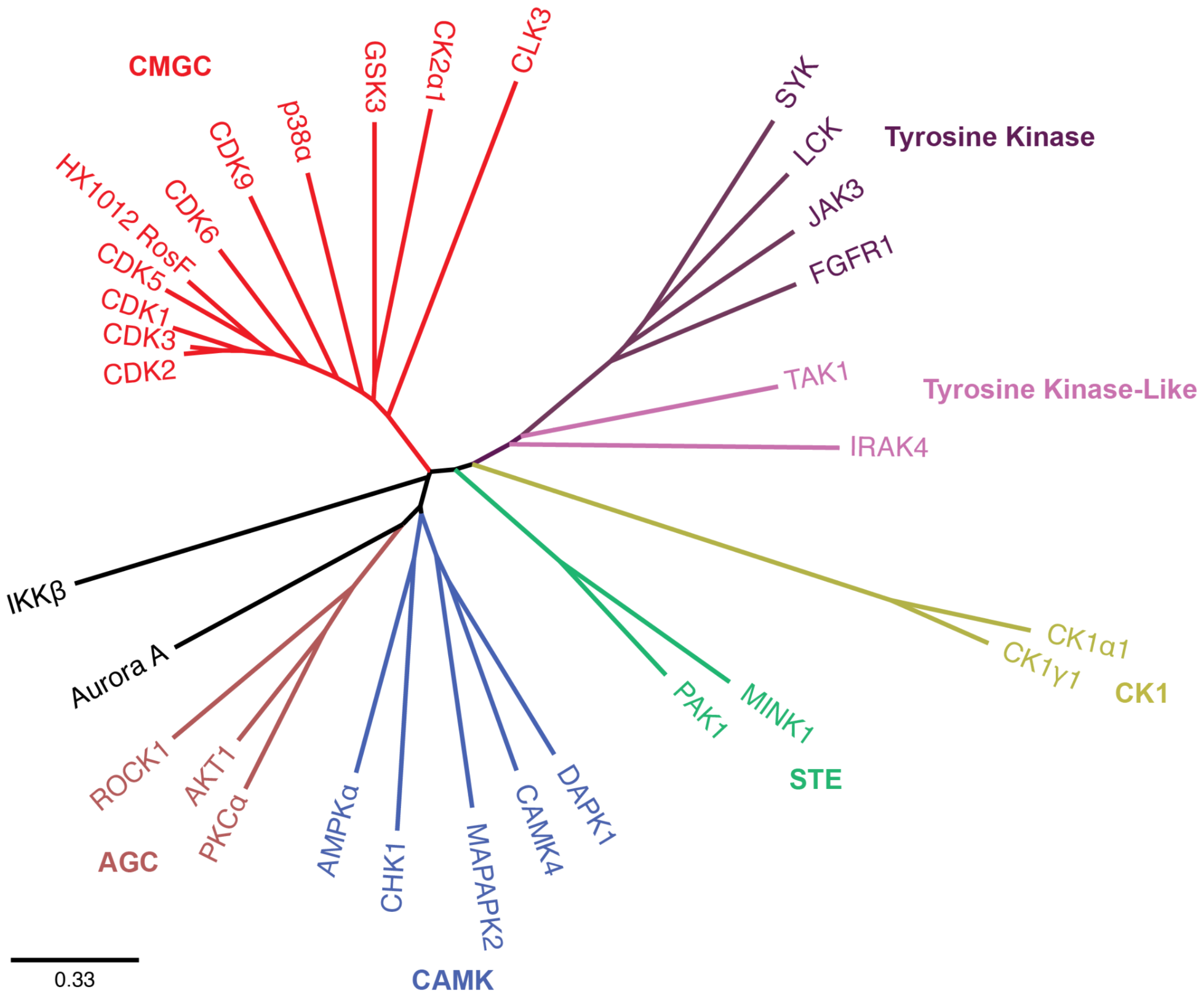

Figure S9. Phylogeny of kinases profiled in the selectivity panel.
Neighbor-joining phylogenetic tree of *A. uvarum* RosF and enzymes profiled in the kinase selectivity panel with 1000 bootstrap values. Branches are colored according to the kinase subfamily to which the kinase belongs.

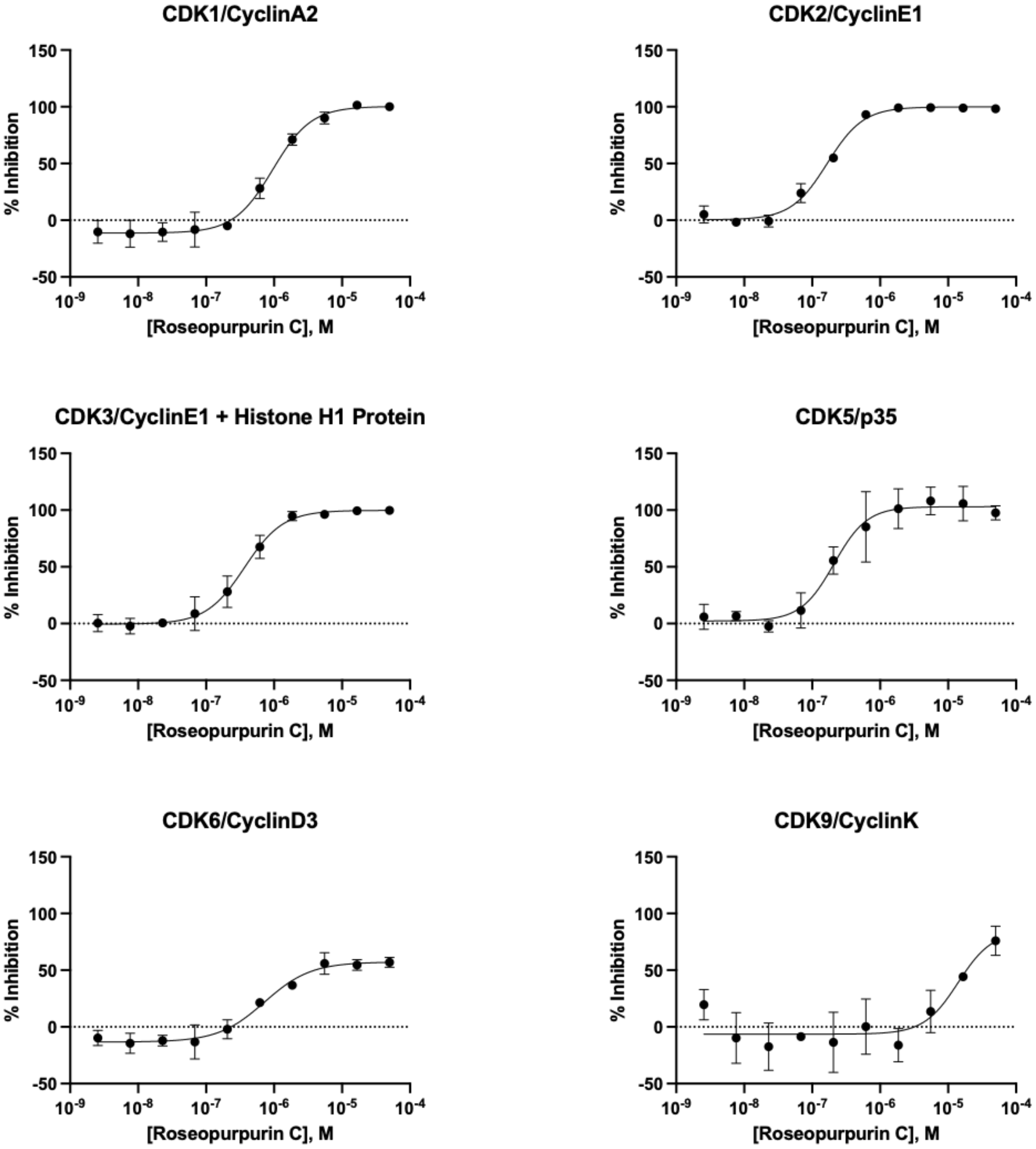
CDK1/CyclinA2
CDK2/CyclinE1
CDK3/CyclinE1 + Histone H1 Protein
CDK5/p35
CDK6/CyclinD3
CDK9/CyclinK
% Inhibition
150
100
50
0
-50
10^{-9}
10^{-8}
10^{-7}
10^{-6}
10^{-5}
10^{-4}
[Roseopurpurin C], M

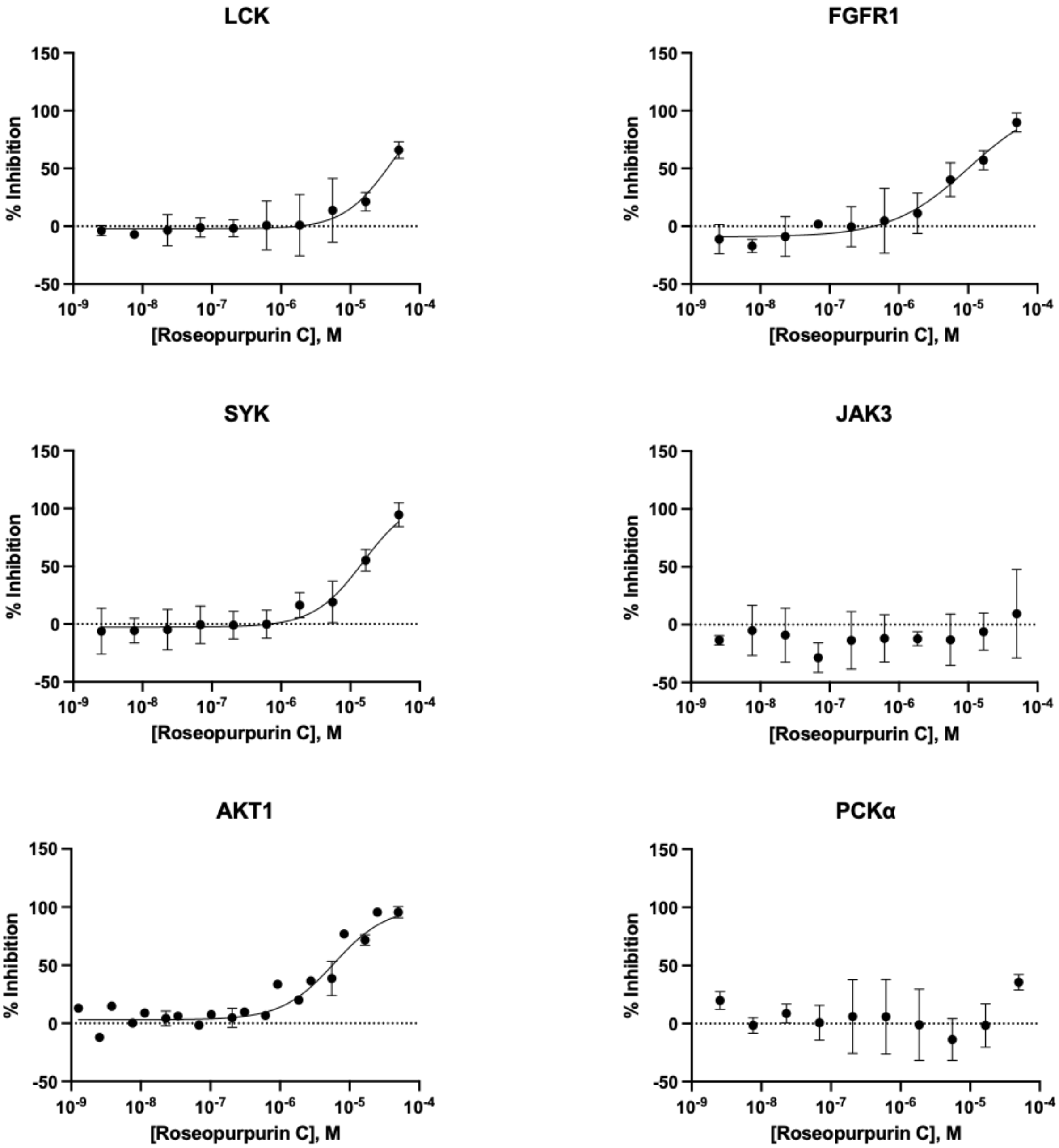
LCK
FGFR1
SYK
JAK3
AKT1
PCKα
% Inhibition
[Roseopurpurin C], M
150
100
50
0
-50
10^{-9}
10^{-8}
10^{-7}
10^{-6}
10^{-5}
10^{-4}

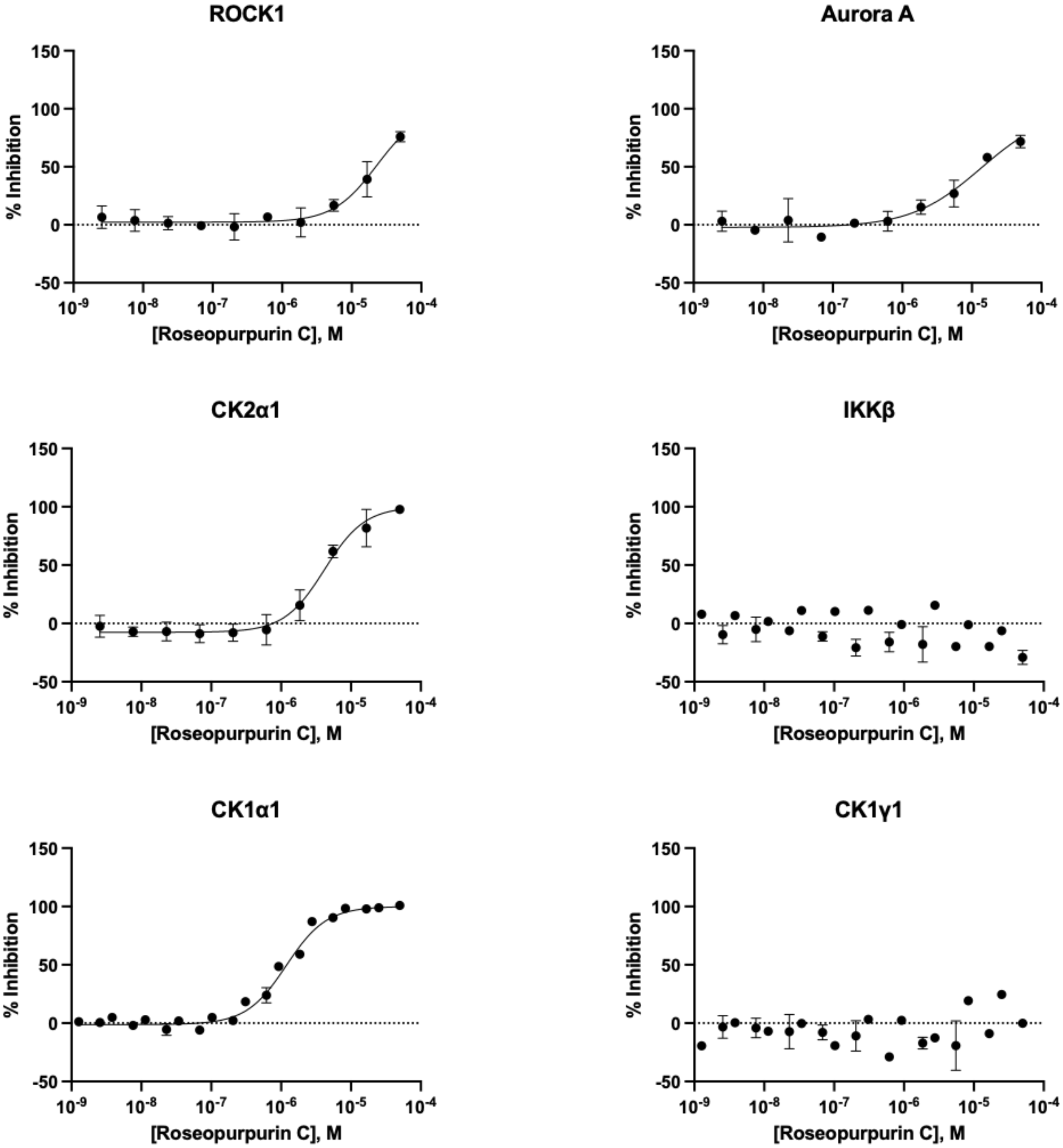
ROCK1
Aurora A
CK2α1
IKKβ
CK1α1
CK1γ1
% Inhibition
[Roseopurpurin C], M
150
100
50
0
-50
10^{-9}
10^{-8}
10^{-7}
10^{-6}
10^{-5}
10^{-4}

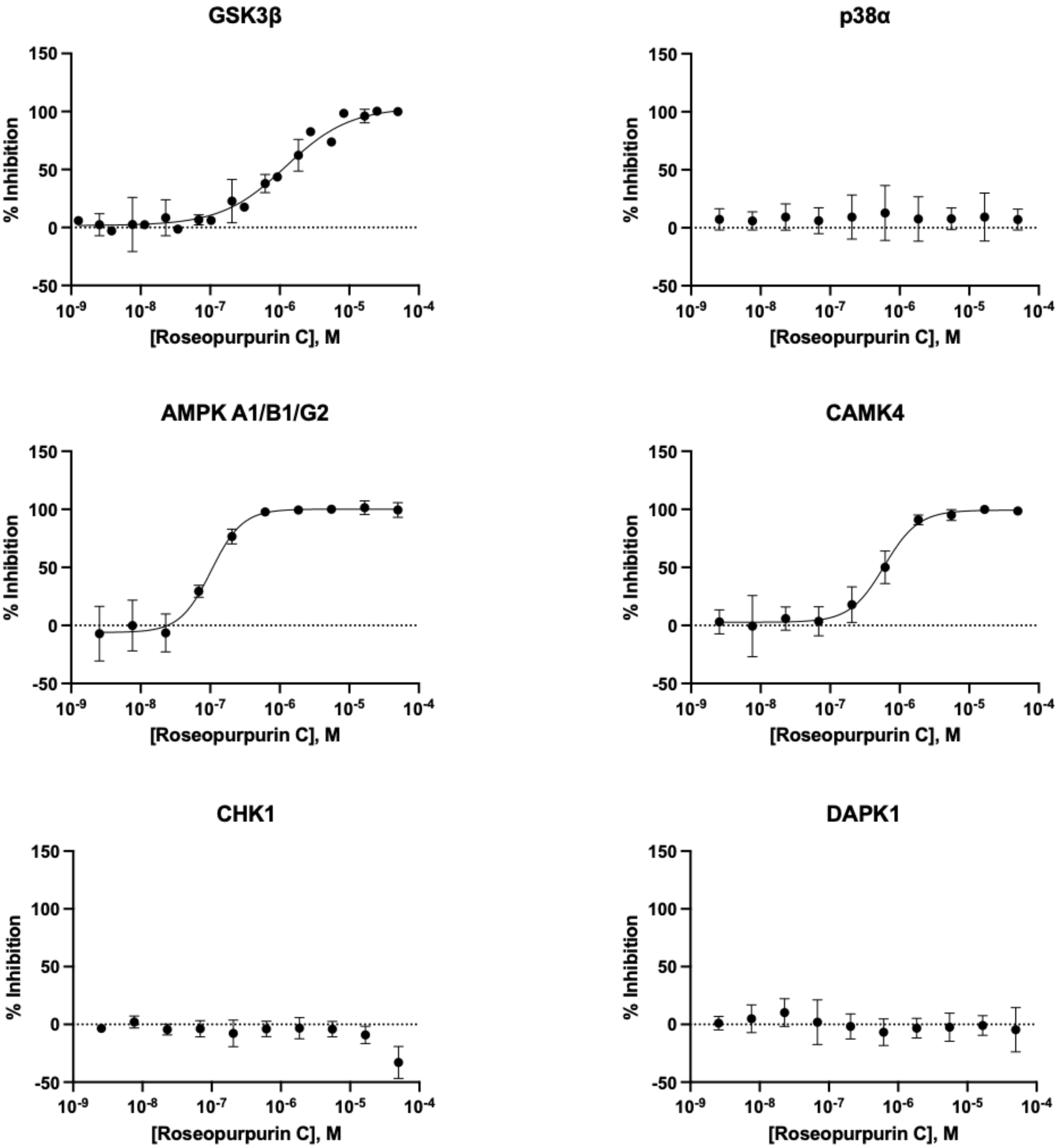
GSK3β
p38α
AMPK A1/B1/G2
CAMK4
CHK1
DAPK1
% Inhibition
150
100
50
0
-50
10^{-9} 10^{-8} 10^{-7} 10^{-6} 10^{-5} 10^{-4}
[Roseopurpurin C], M

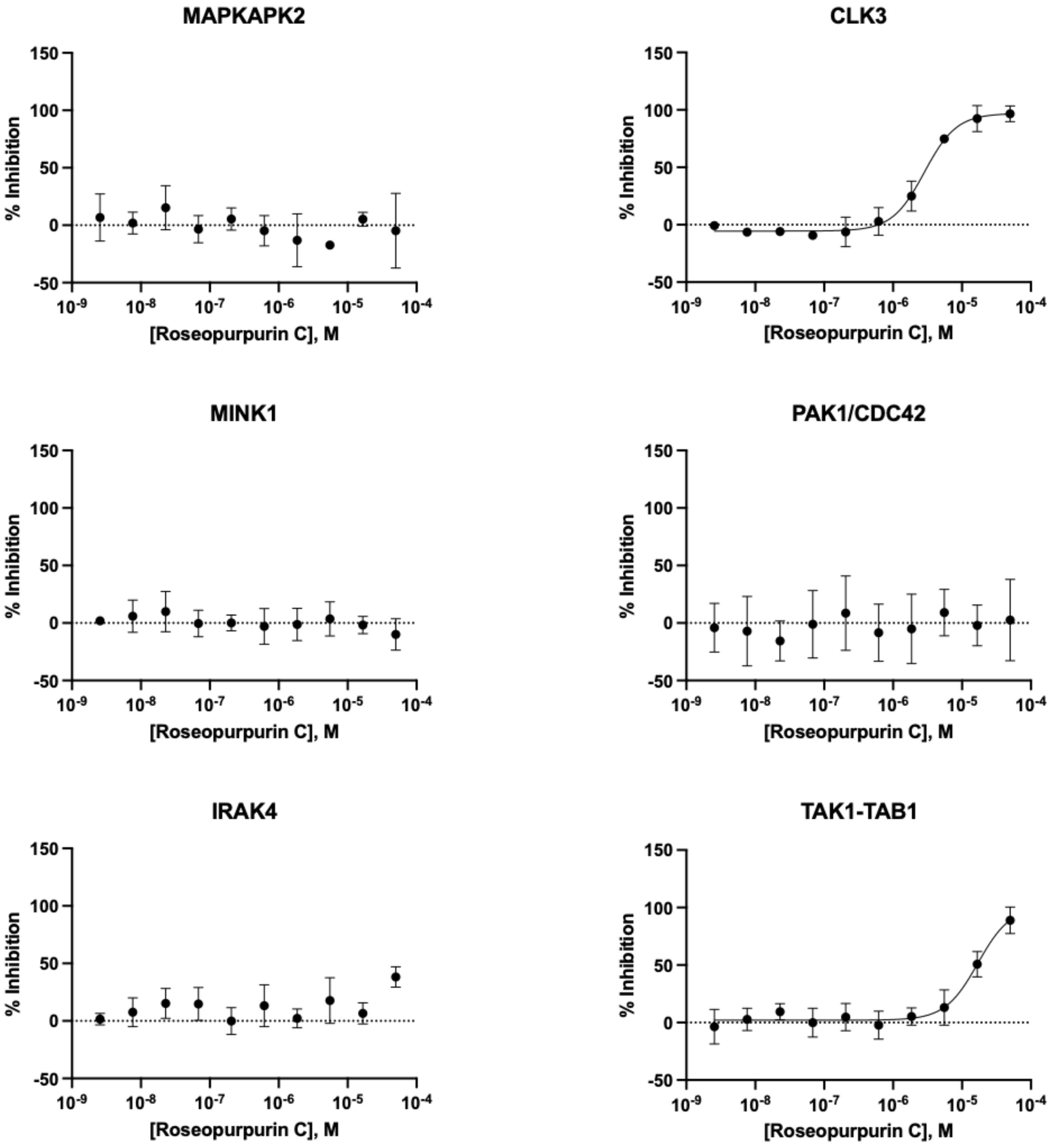

Figure S10. Roseopurpurin C (1) kinase selectivity profile.
Data represents the averages ± standard deviation; n=3. IC_{50} values were determined using PRISM 9 and a 4-parameter curve fit. IC_{50} values are reported in Table 1.

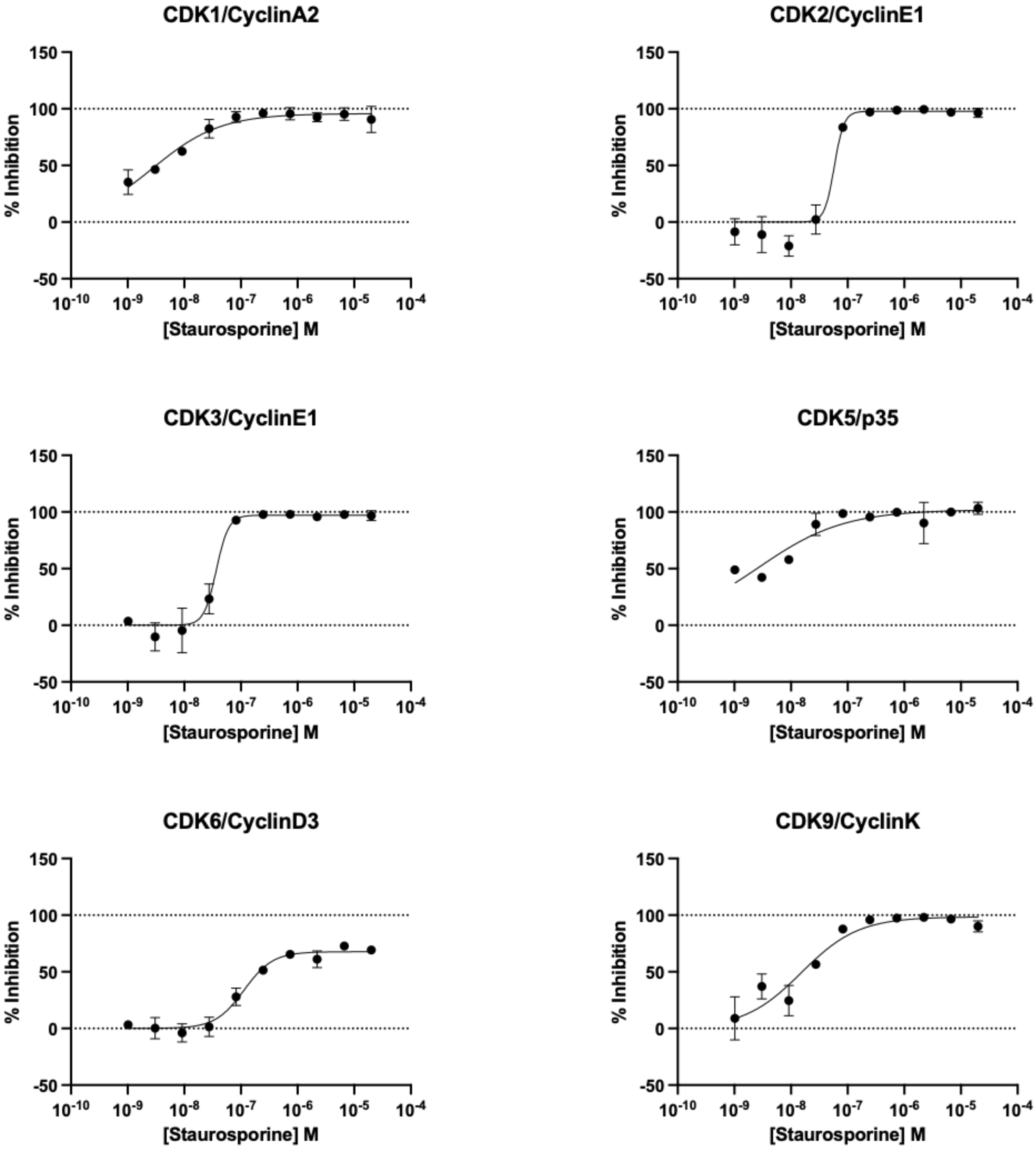
CDK1/CyclinA2
CDK2/CyclinE1
CDK3/CyclinE1
CDK5/p35
CDK6/CyclinD3
CDK9/CyclinK
% Inhibition
150
100
50
0
-50
10^{-10} 10^{-9} 10^{-8} 10^{-7} 10^{-6} 10^{-5} 10^{-4}
[Staurosporine] M

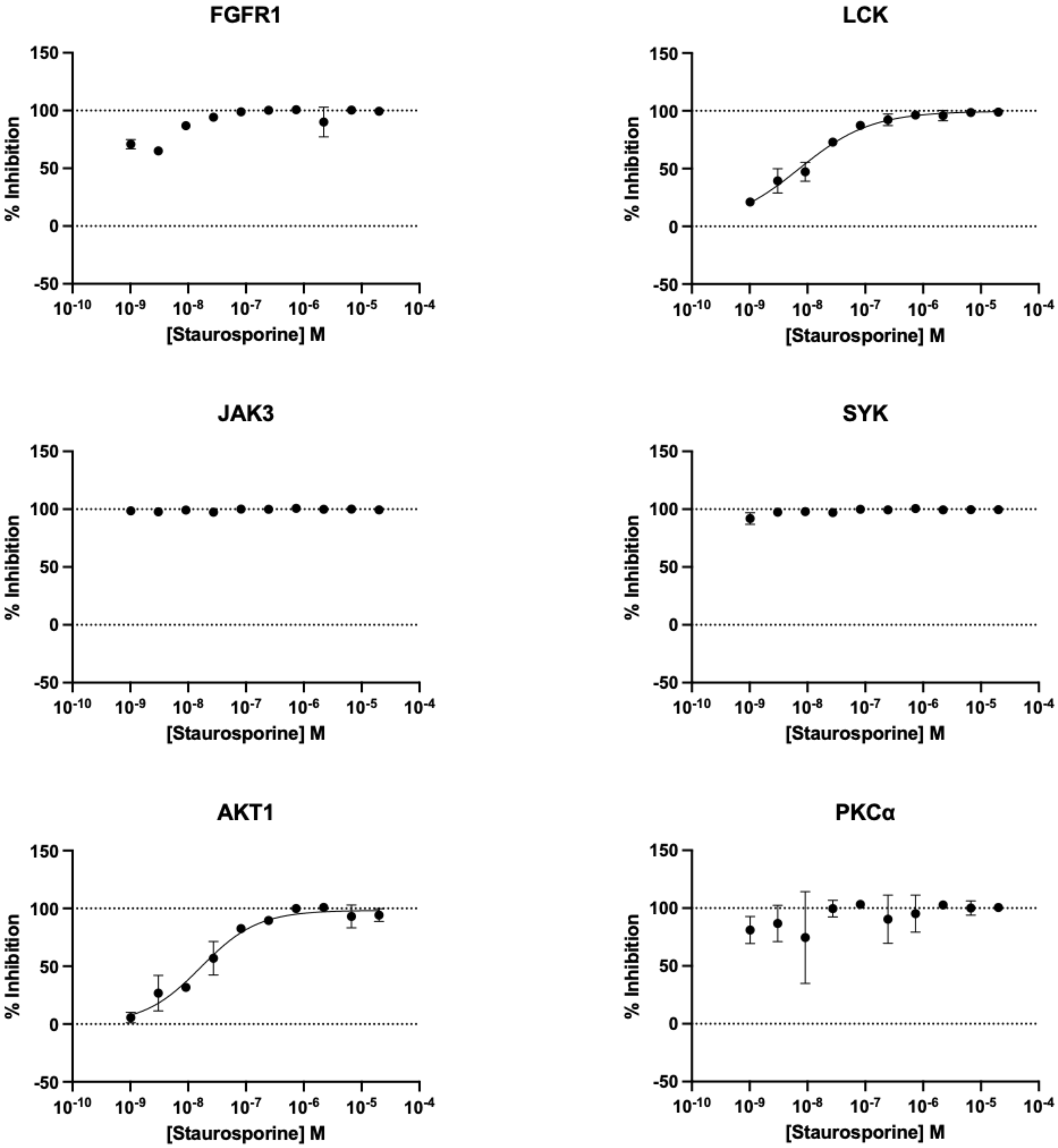
FGFR1
LCK
JAK3
SYK
AKT1
PKCα
% Inhibition
[Staurosporine] M
150
100
50
0
-50
10-10
10-9
10-8
10-7
10-6
10-5
10-4

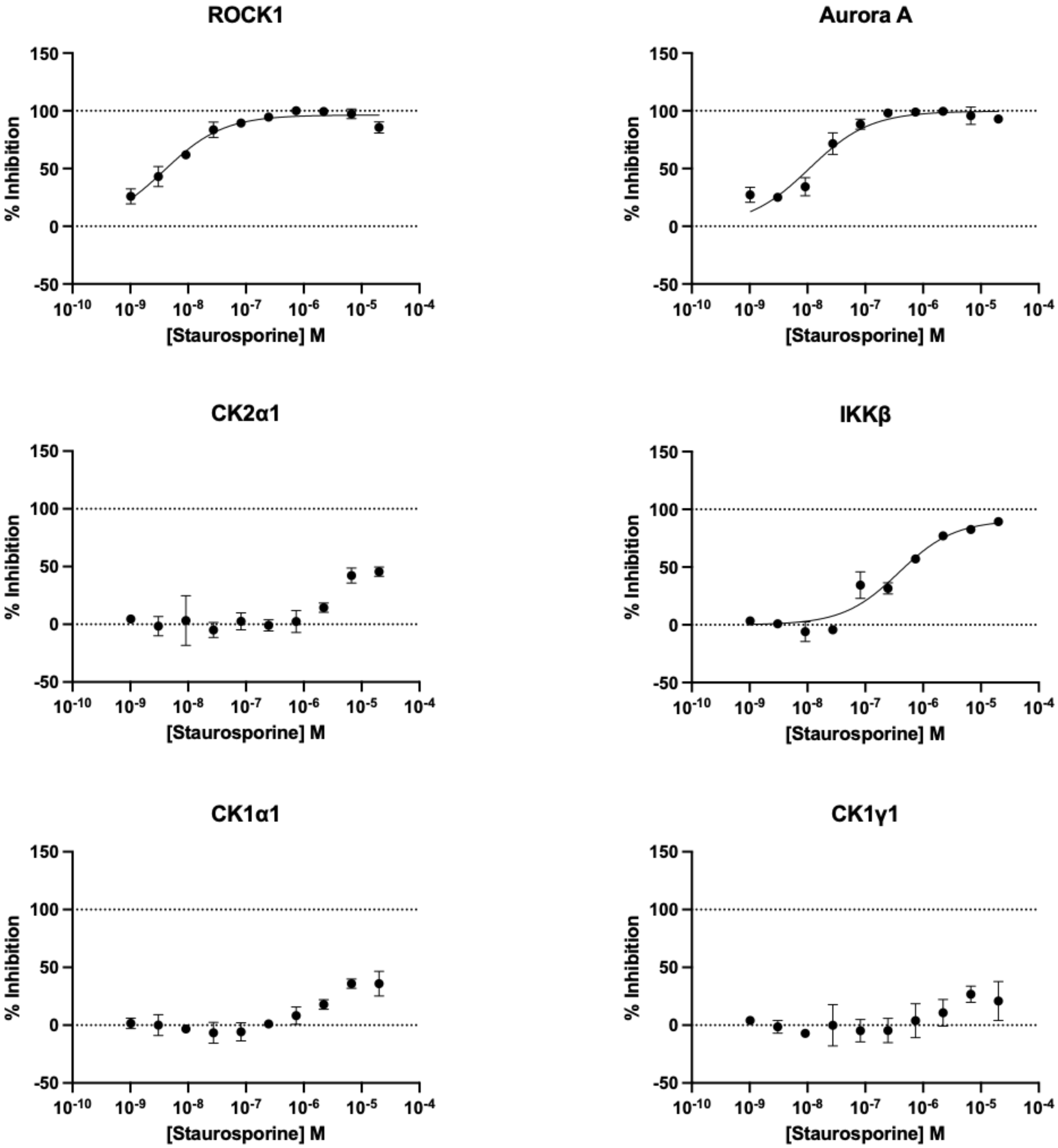
ROCK1
Aurora A
CK2α1
IKKβ
CK1α1
CK1γ1
% Inhibition
[Staurosporine] M
150
100
50
0
-50
10-10
10-9
10-8
10-7
10-6
10-5
10-4

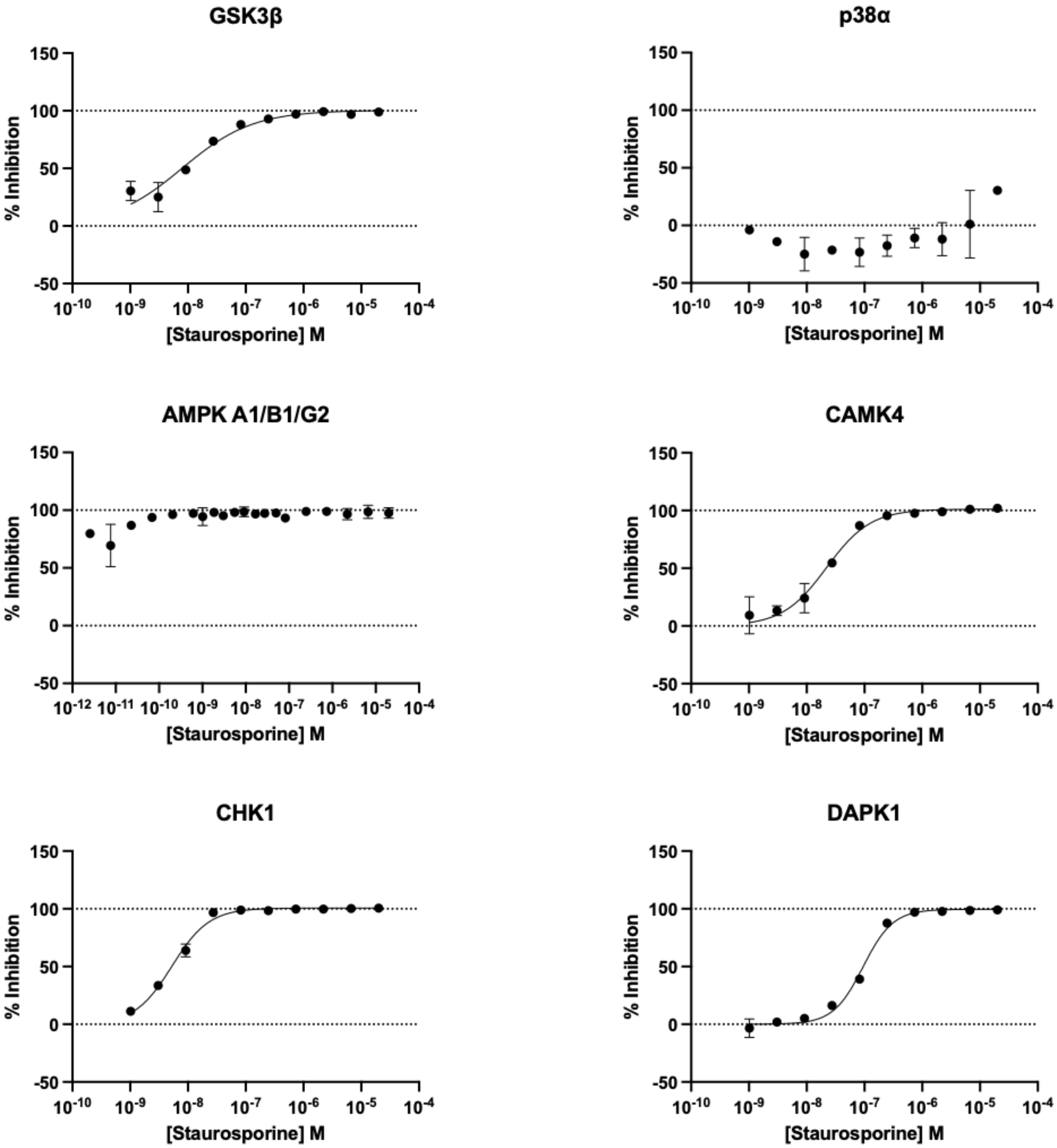
GSK3β
p38α
AMPK A1/B1/G2
CAMK4
CHK1
DAPK1
% Inhibition
[Staurosporine] M

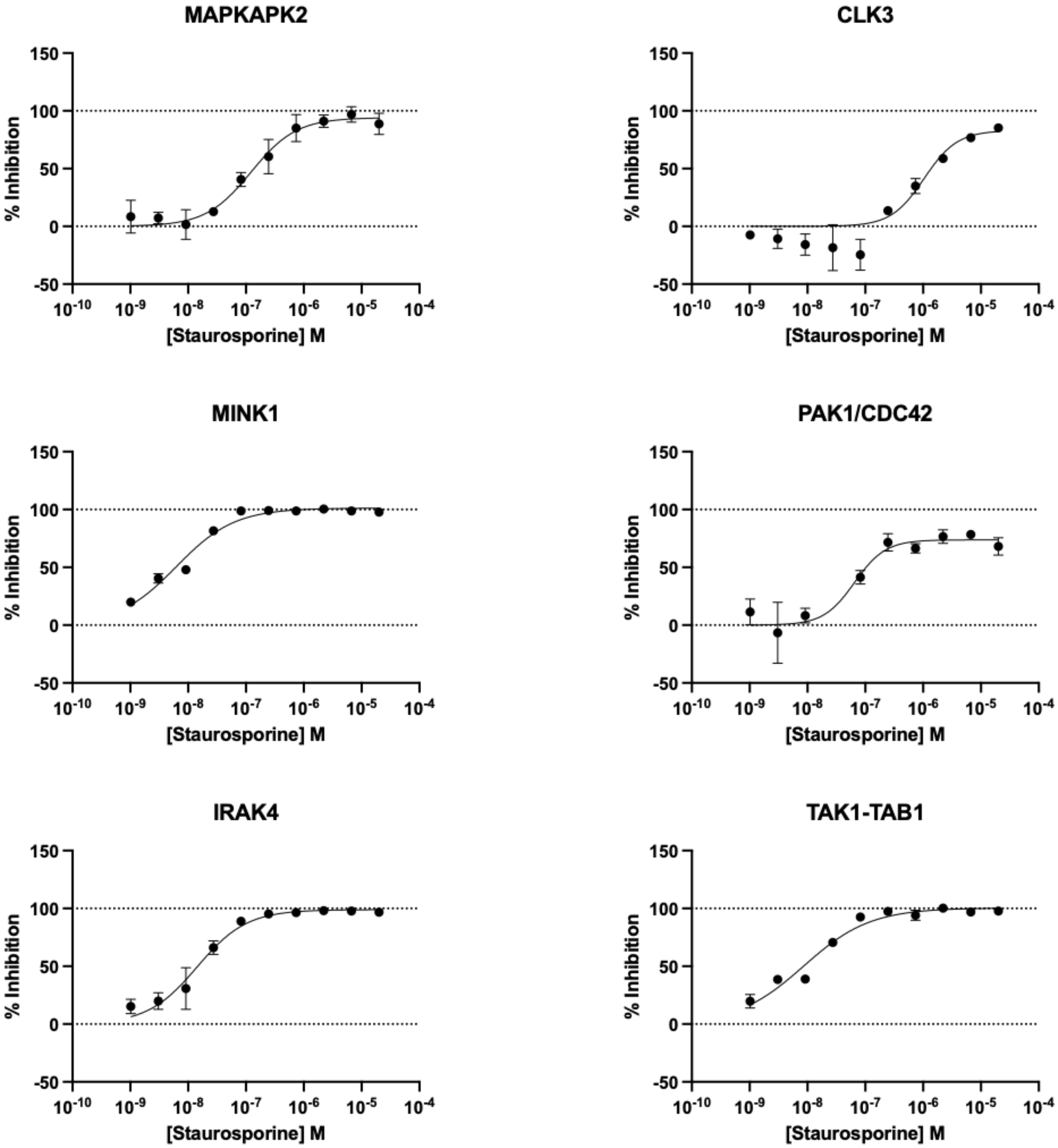

Figure S11. Staurosporine kinase selectivity profile.
Data represents the averages ± standard deviation; n=3. IC50 values were determined using PRISM 9 and a 4-parameter curve fit. IC_{50} values are reported in Table 1.

```
CDK2       ------------MENFQKVEKIGEGTYGVVYKARNK-LTGEVVALKKIRLDTE-TEGVPS   46
CDK1       ------------MEDYTKIEKIGEGTYGVVYKGRHK-TTGQVVAMKKIRLESE-EEGVPS   46
CDK3       ------------MDMFQKVEKIGEGTYGVVYKAKNR-ETGQLVALKKIRLDLE-MEGVPS   46
CDK5       ------------MQKYEKLEKIGEGTYGTVFKAKNR-ETHEIVALKRVRLDDD-DEGVPS   46
CDK6       ---MEKDGLCRADQQYECVAEIGEGAYGKVFKARDLKNGGRFVALKRVRVQTG-EEGMPL   56
AMPK       -----------------LGDTLGVGTFGKVKIGEHQ-LTGHKVAVKILNRQKIRSLDVVG   59
CAMK4      DYWIDGSNRDALSDFFEVESELGRGATSIVYRCKQK-GTQKPYALKVLKKTV-----DKK   84
                                :* *: . *   ..     .  *:* :

CDK2       TAIREISLLKE---LNHPNIVKLLDVIH--------TENKLYLVFEFLHQDLKKFMDASA   95
CDK1       TAIREISLLKE---LRHPNIVSLQDVLM--------QDSRLYLIFEFLSMDLKKYLDSIP   95
CDK3       TAIREISLLKE---LKHPNIVRLLDVVH--------NERKLYLVFEFLSQDLKKYMDSTP   95
CDK5       SALREICLLKE---LKHKNIVRLHDVLH--------SDKKLTLVFEFCDQDLKKYFDSCN   95
CDK6       STIREVAVLRHLETFEHPNVVRLFDVCTVSRT---DRETKLTLVFEHVDQDLTTYLDKVP  113
AMPK       KIKREIQNLKL---FRHPHIIKLYQVIS--------TPTDFFMVMEYVSGG--ELFDYIC  106
CAMK4      IVRTEIGVLLR---LSHPNIIKLKEIFE--------TPTEISLVLELVTGG--ELFDRIV  131
               *:  *     : * ::: * ::              : ::::    .    :.

CDK2       -LTGIPLPLIKSYLFQLLQGLAFCHSHRVLHRDLKPQNLLINTE---GAIKLADFGLARA  151
CDK1       PGQYMDSSLVKSYLYQILQGIVFCHSRRVLHRDLKPQNLLIDDK---GTIKLADFGLARA  152
CDK3       -GSELPLHLIKSYLFQLLQGVSFCHSHRVIHRDLKPQNLLINEL---GAIKLADFGLARA  151
CDK5       --GDLDPEIVKSFLFQLLKGLGFCHSRNVLHRDLKPQNLLINRN---GELKLADFGLARA  150
CDK6       -EPGVPTETIKDMMFQLLRGLDFLHSHRVVHRDLKPQNILVTSS---GQIKLADFGLARI  169
AMPK       KHGRVEEMEARRLFQQILSAVDYCHRHMVVHRDLKPENVLLDAH---MNAKIADFGLSNM  163
CAMK4      EKGYYSERDAADAVKQILEAVAYLHENGIVHRDLKPENLLYATPAPDAPLKIADFGLSKI  191
                        .  :* .: : * . ::***:*  *:*          *:*****:.
```

Figure S12. Sequence alignment of AMPKα1 and CAMK4 with select CDKs.
Residues within 4Å of 1 in the CDK2 structure are bolded. Residues that are mutated relative to CDK2 are colored red. The hinge region of the kinase is boxed. The glutamate residue that potentially serves the critical 4-OH binding role of Asp89 in AMPKα1 and CAMK4 is highlighted in yellow (see **Figure S5 and S6**).

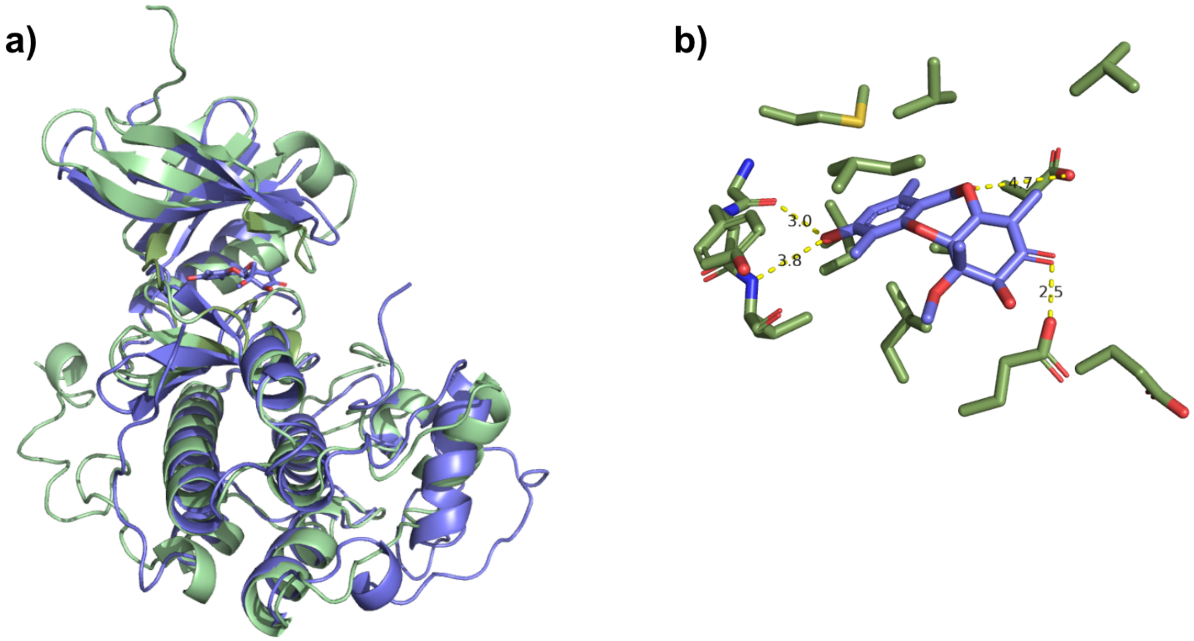

Figure S13. Structural alignment of AMPKα1 and 1-bound CDK2.
a) Structural alignment of **1**-bound CDK2 (blue) and AMPKα1 (green; PDB 4CFF). **b)** Zoom in on the ATP-binding pocket of AMPKα1 with residues predicted to bind **1** based on the structural alignment are displayed. Although AMPKα1 harbors a Asp89Gly mutation (CDK2 numbering), the downstream Glu (Glu100 in AMPKα1) is appropriately positioned to form a H-bond with the 4-OH of **1**.

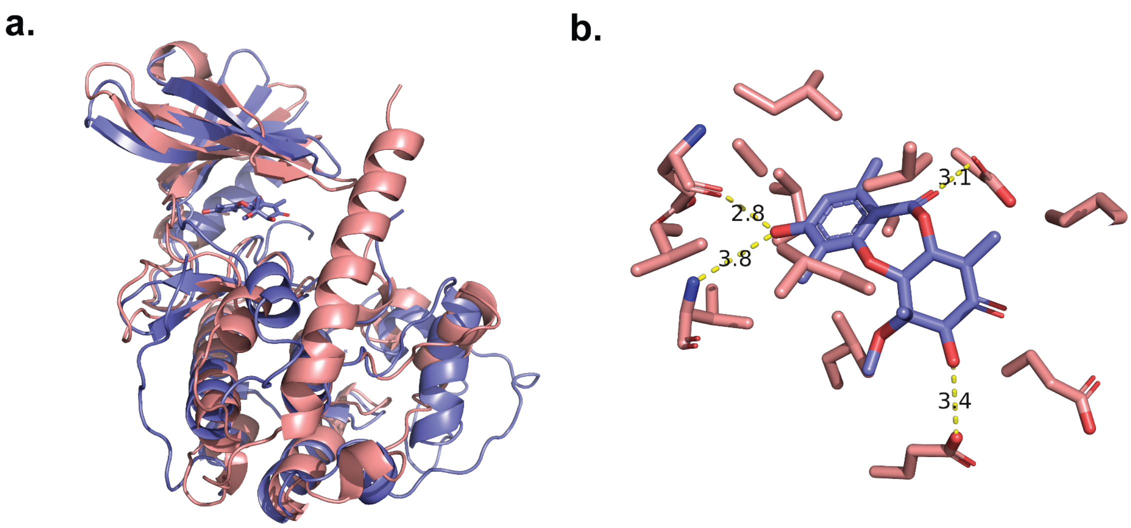

Figure S14. Structural alignment of CAMK4 and 1-bound CDK2.

a) Structural alignment of **1**-bound CDK2 (blue) and CAMK4 (pink; PDB 2W4O). **b)** Zoom in on the ATP-binding pocket of CAMK4 with residues predicted to bind **1** based on the structural alignment are displayed. Although CAMK4 harbors a Asp89Gly mutation (CDK2 numbering), the downstream Glu (Glu125 in CAMK4) is appropriately positioned to form a H-bond with the 4-OH of **1**.

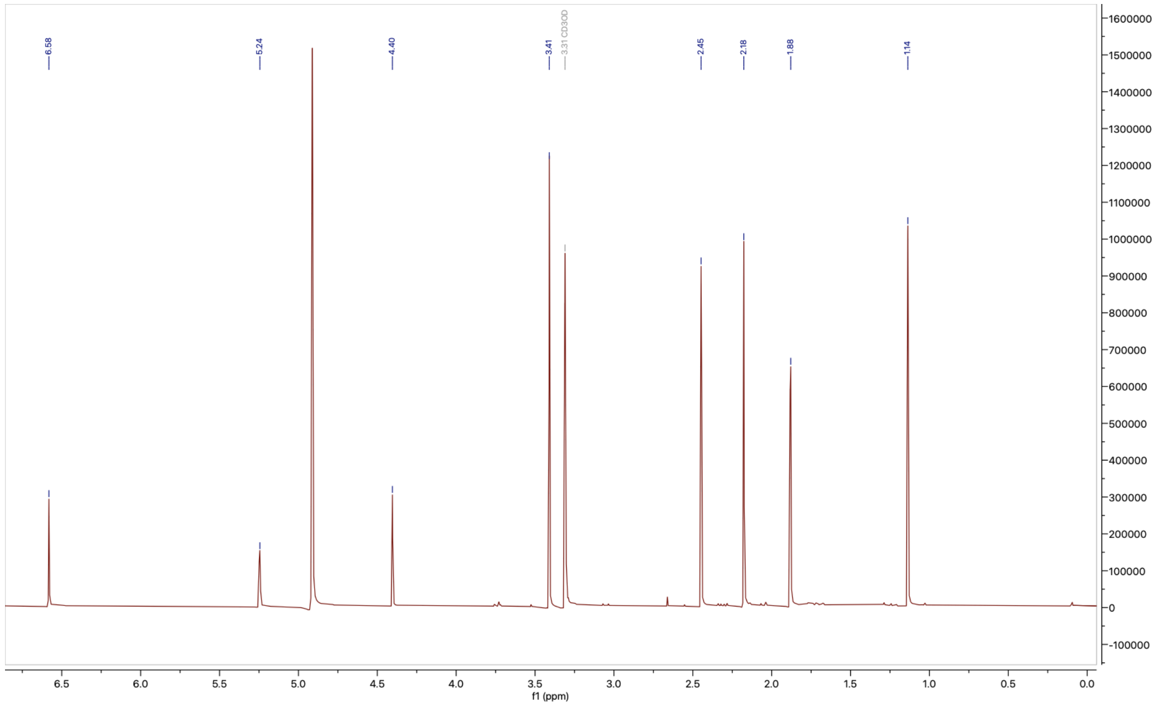

Figure S15. The ^1H NMR spectrum of 1 in CD_3OD (600 MHz)

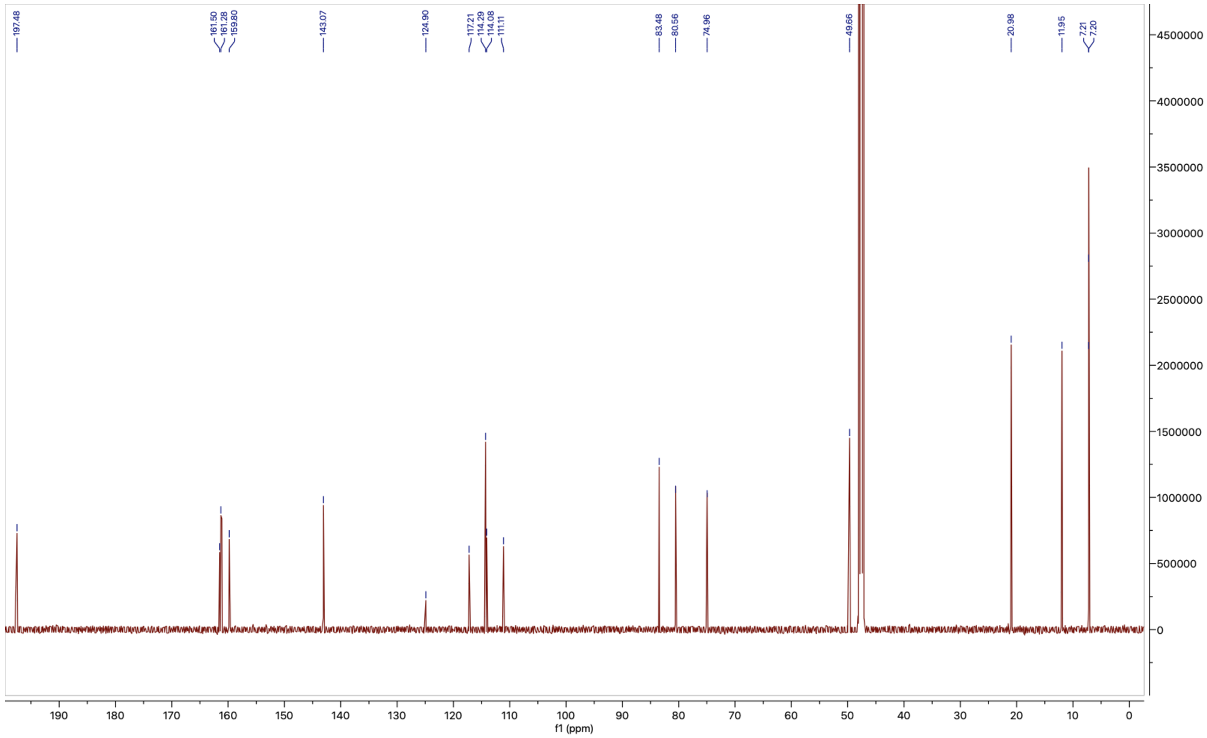

Figure S16. The 13C NMR spectrum of 1 in CD3OD (150 MHz)

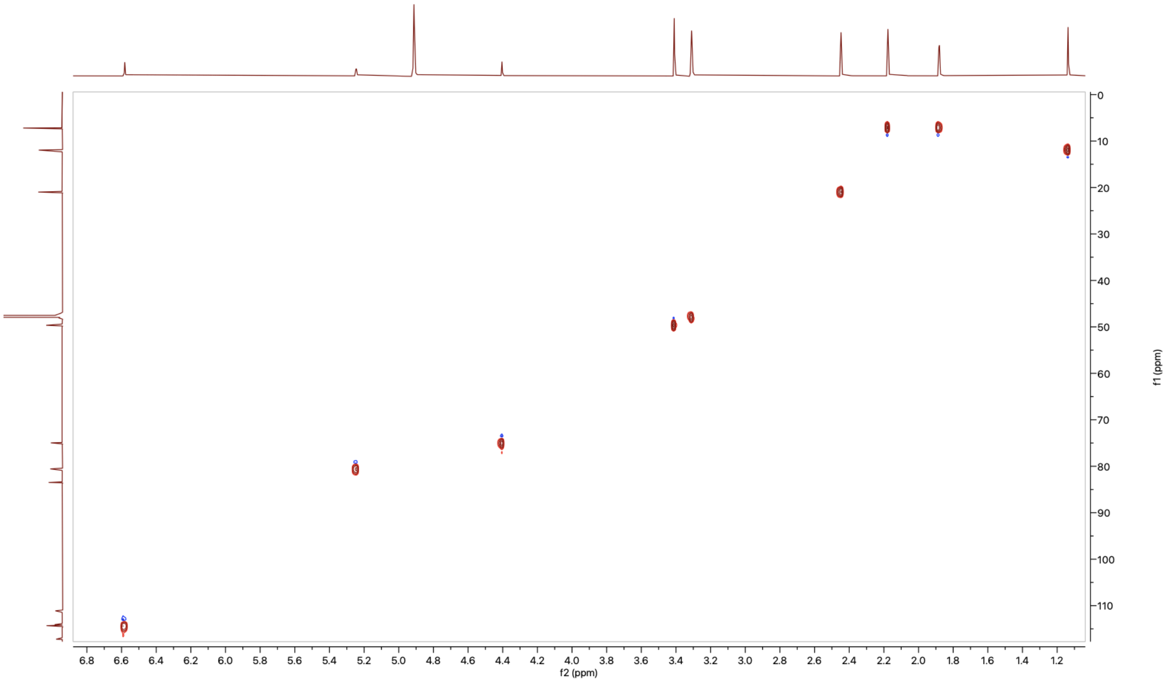

Figure S17. The ^{1}H-^{13}C HSQC NMR spectrum of 1 in CD_3OD (600 MHz)

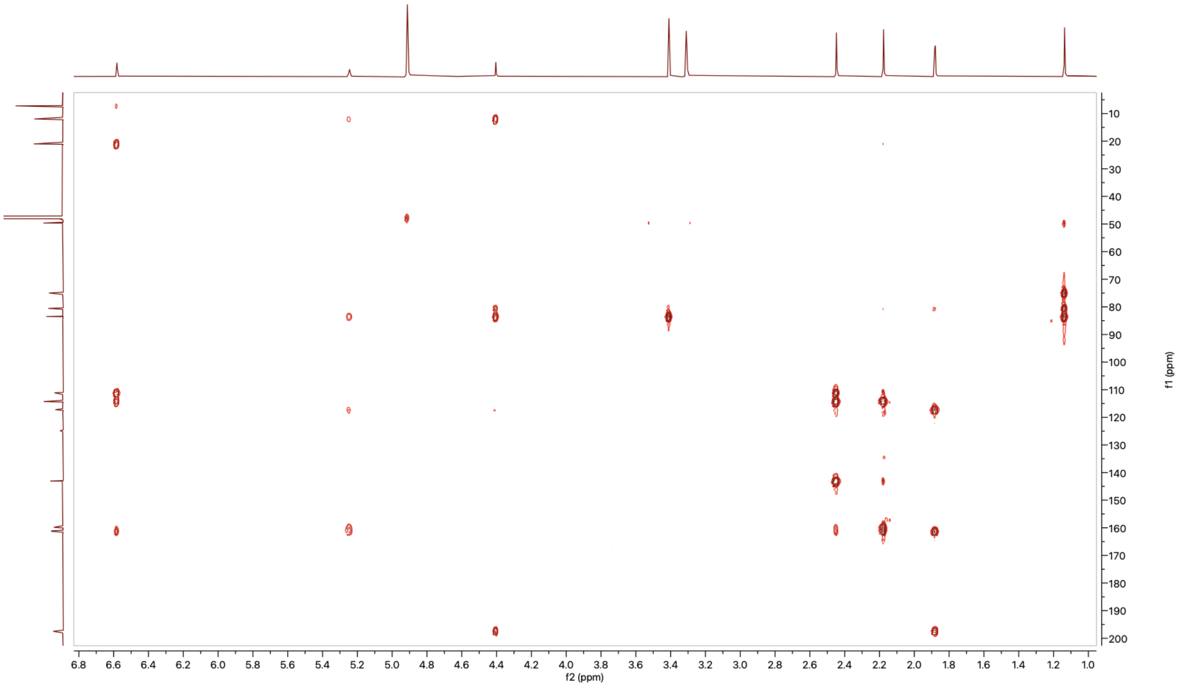

Figure S18. The ^{1}H-^{13}C HMBC NMR spectrum of 1 in CD_3OD (600 MHz)

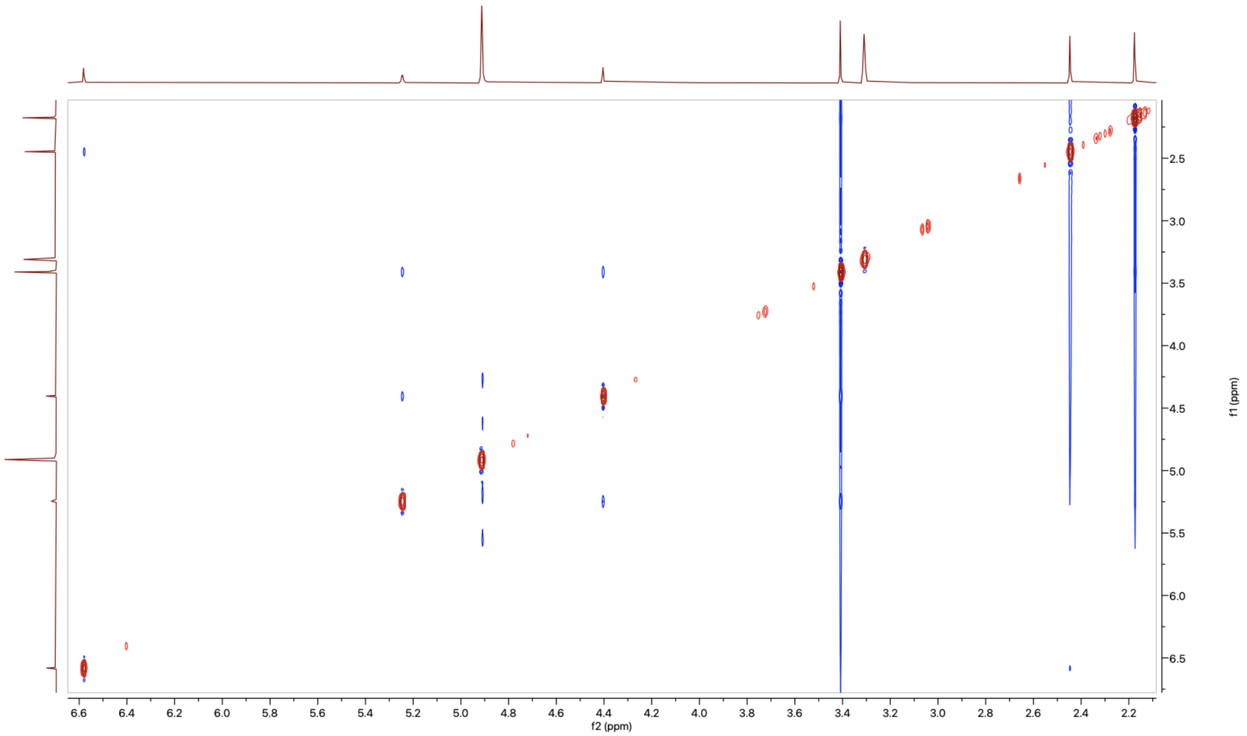

Figure S19. The ^{1}H-^{1}H NOESY NMR spectrum of 1 in CD_3OD (600 MHz)

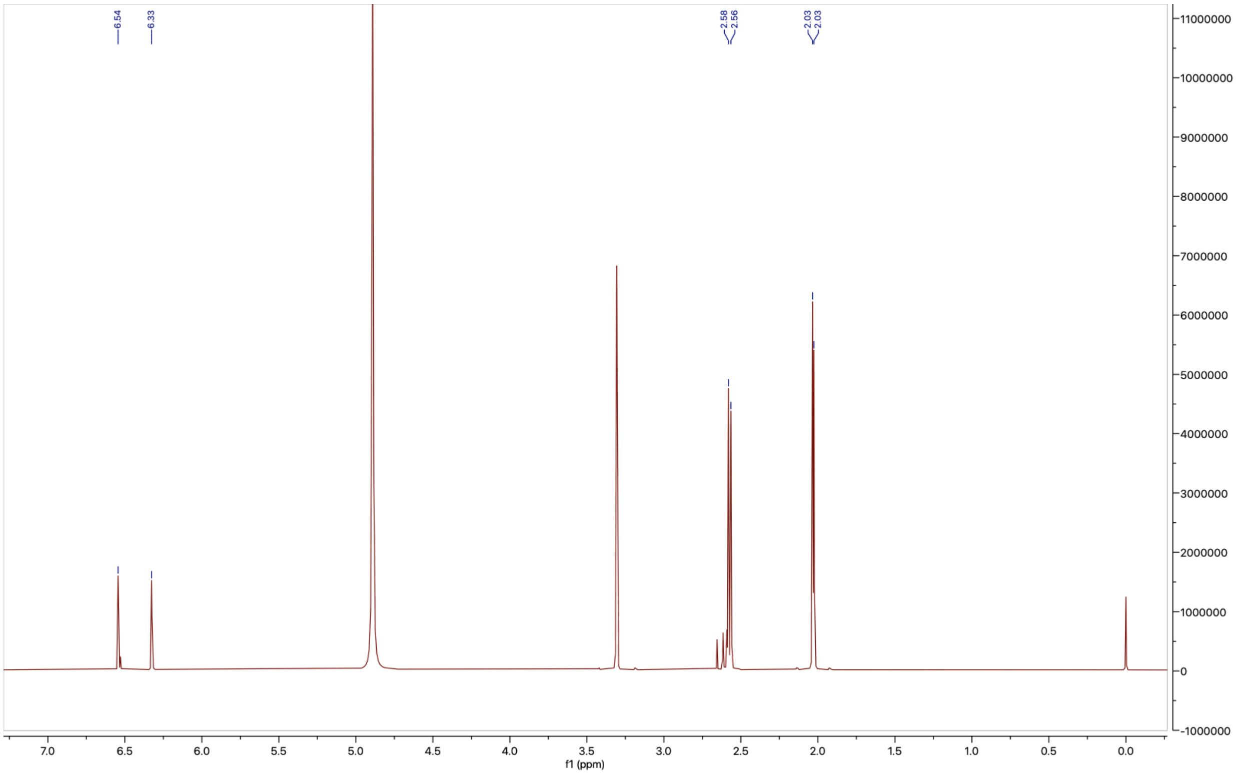

Figure S20. The 1H NMR spectrum of 6 in CD3OD (600 MHz)

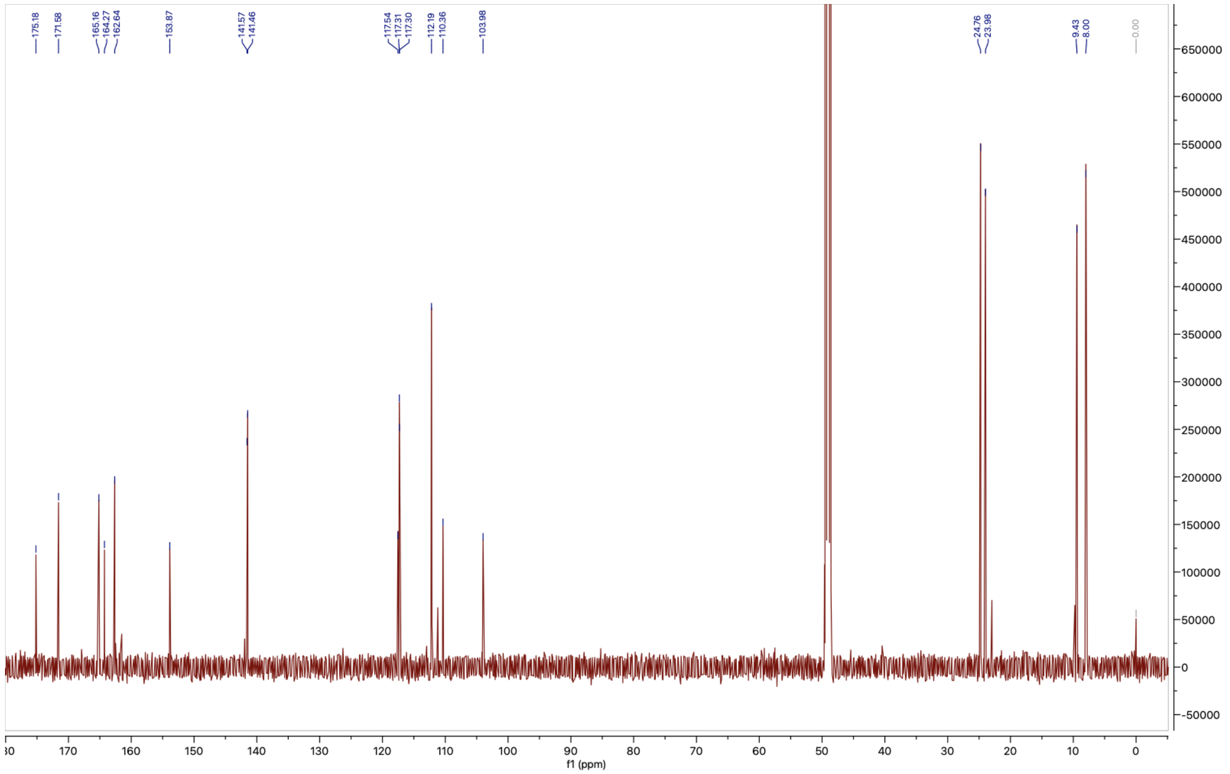

Figure S21. The ^{13}C NMR spectrum of 6 in CD_3OD (150 MHz)

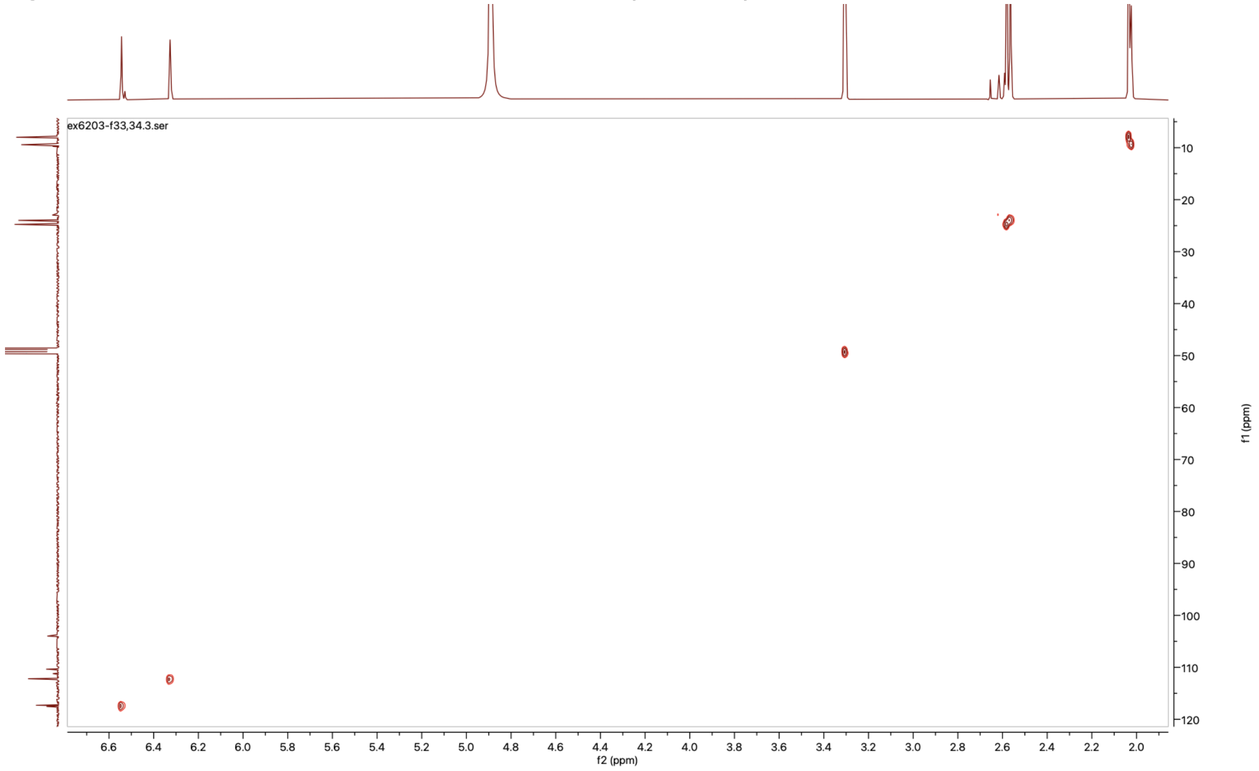

Figure S22. The ^{1}H-^{13}C HSQC NMR spectrum of 6 in CD_3OD (600 MHz)

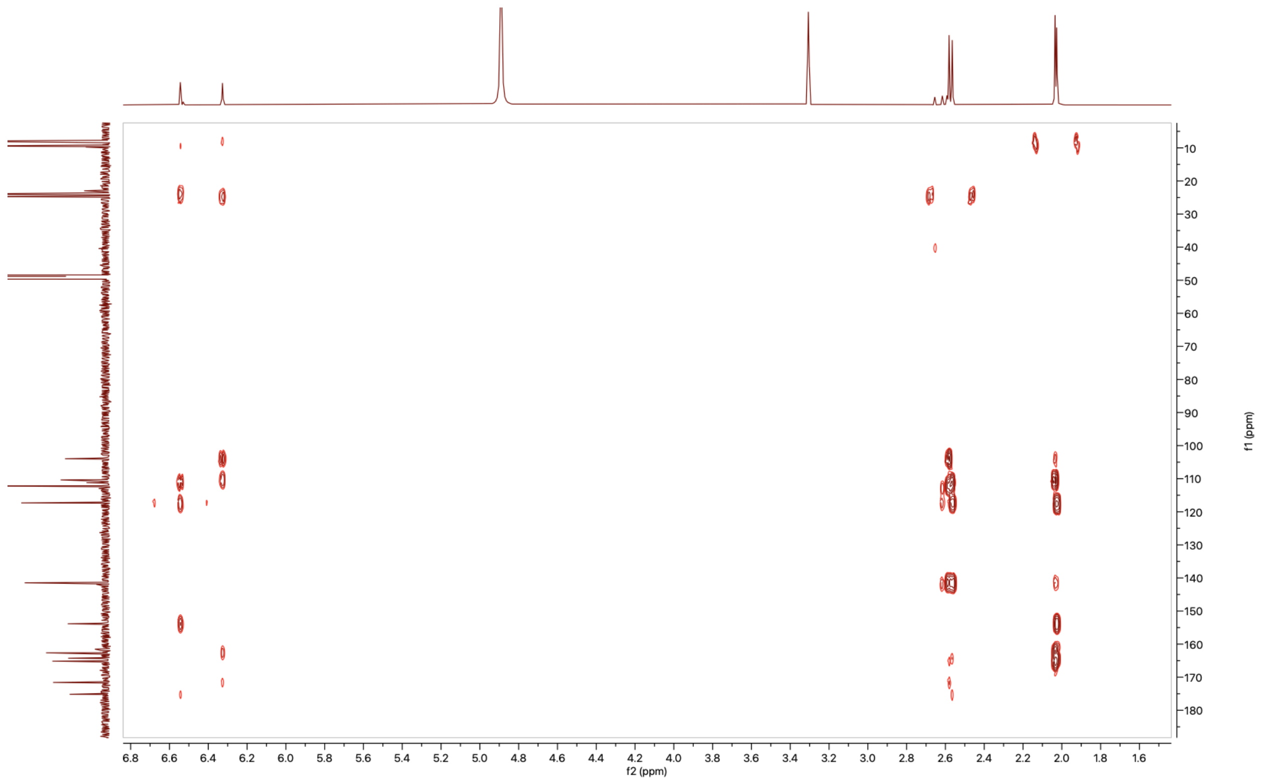

Figure S23. The ^1H-^{13}C HMBC NMR spectrum of 6 in CD_3OD (600 MHz)

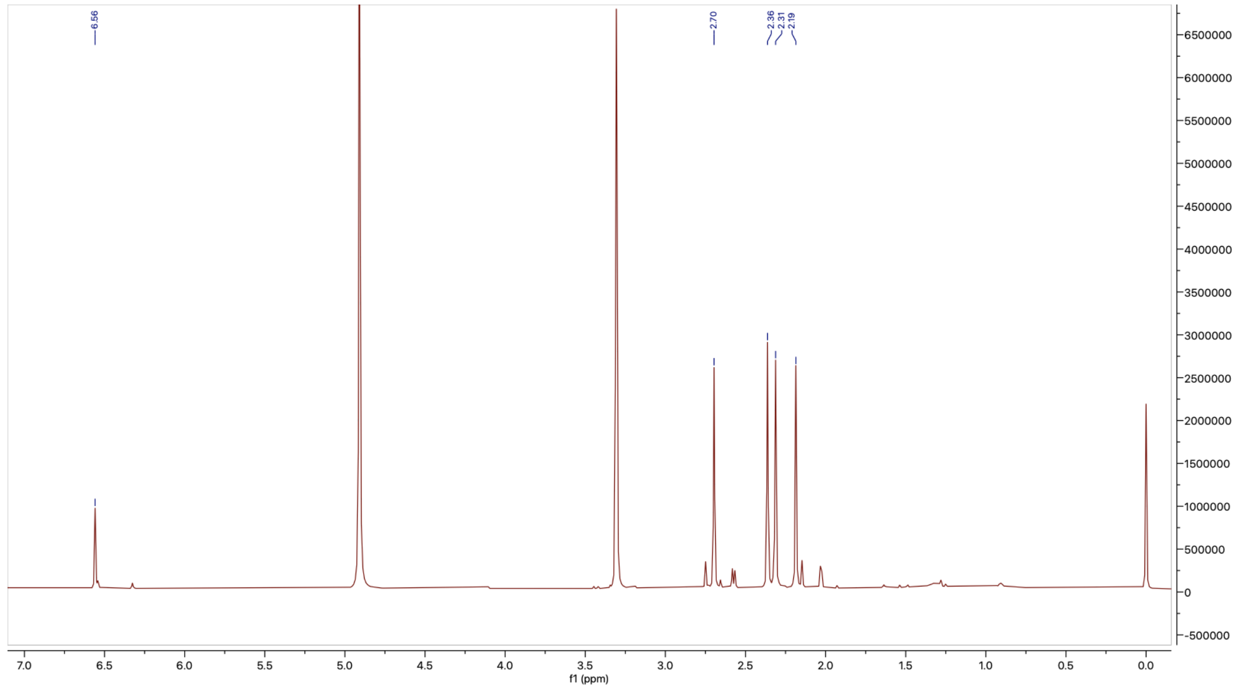

Figure S24. The ^1H NMR spectrum of 7 in CD_3OD (600 MHz)

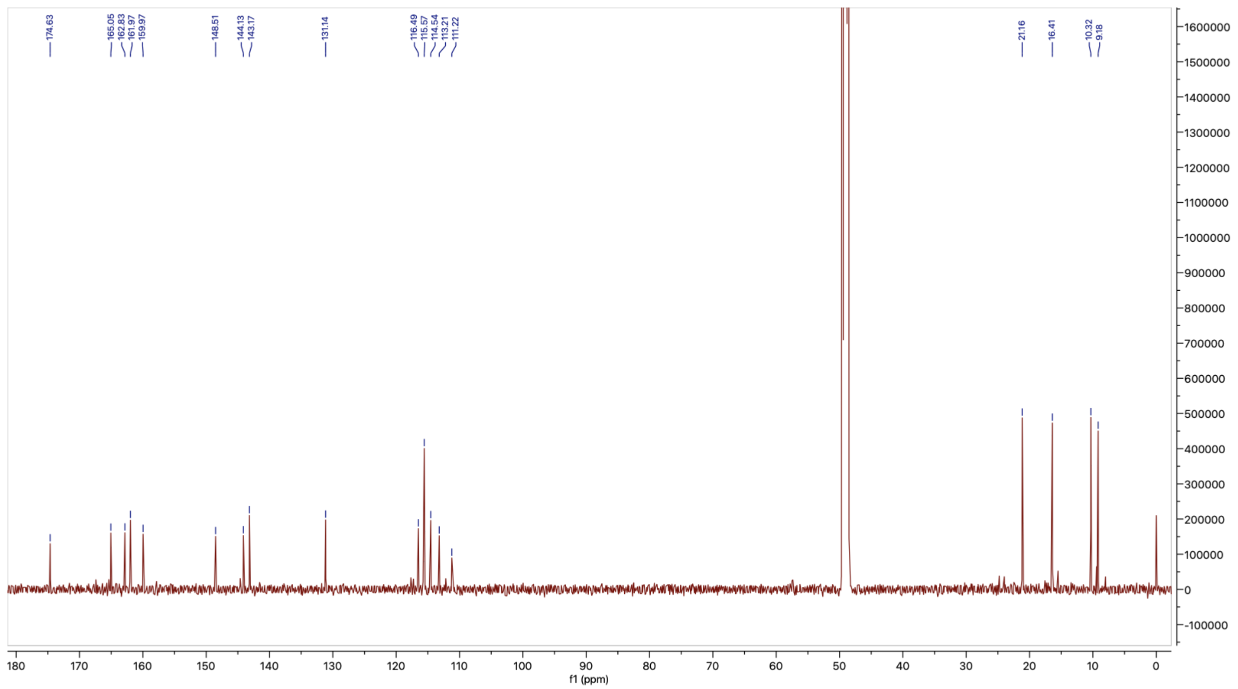

Figure S25. The ^{13}C NMR spectrum of 7 in CD_3OD (150 MHz)

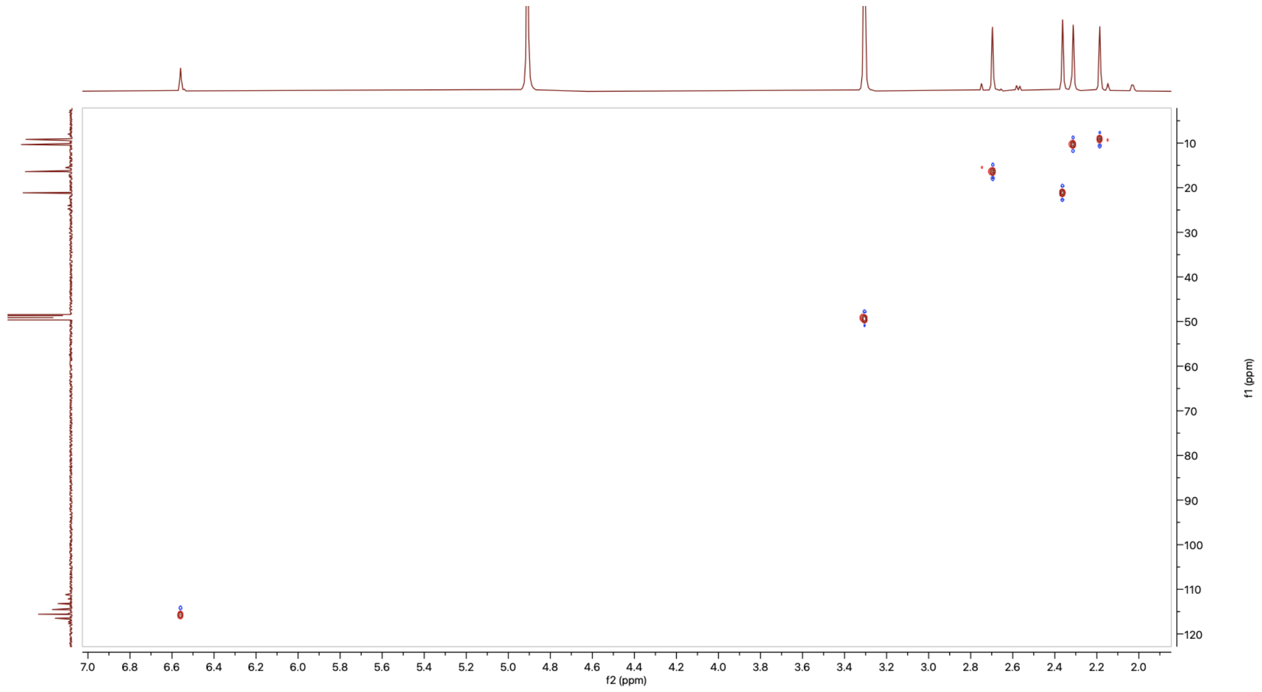

Figure S26. The ^{1}H-^{13}C HSQC NMR spectrum of 7 in CD_3OD (600 MHz)

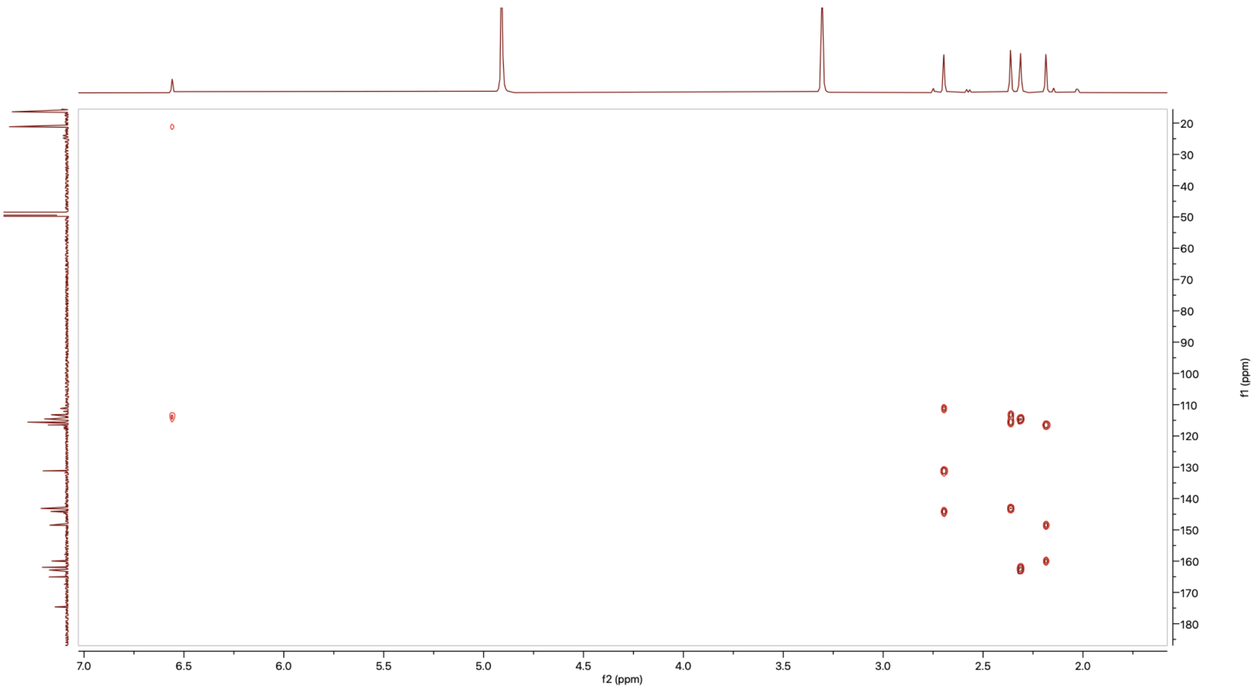

Figure S27. The ^1H-^{13}C HMBC NMR spectrum of 7 in CD_3OD (600 MHz)

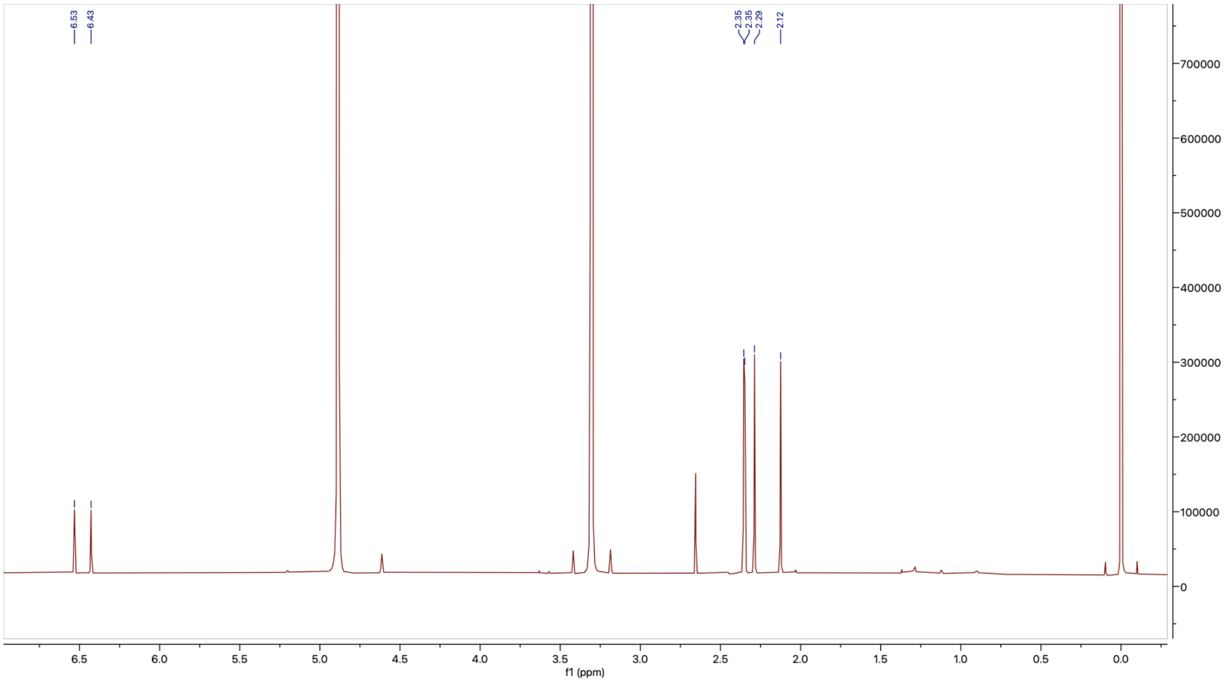

Figure S28. The ^1H NMR spectrum of 8 in CD_3OD (600 MHz)

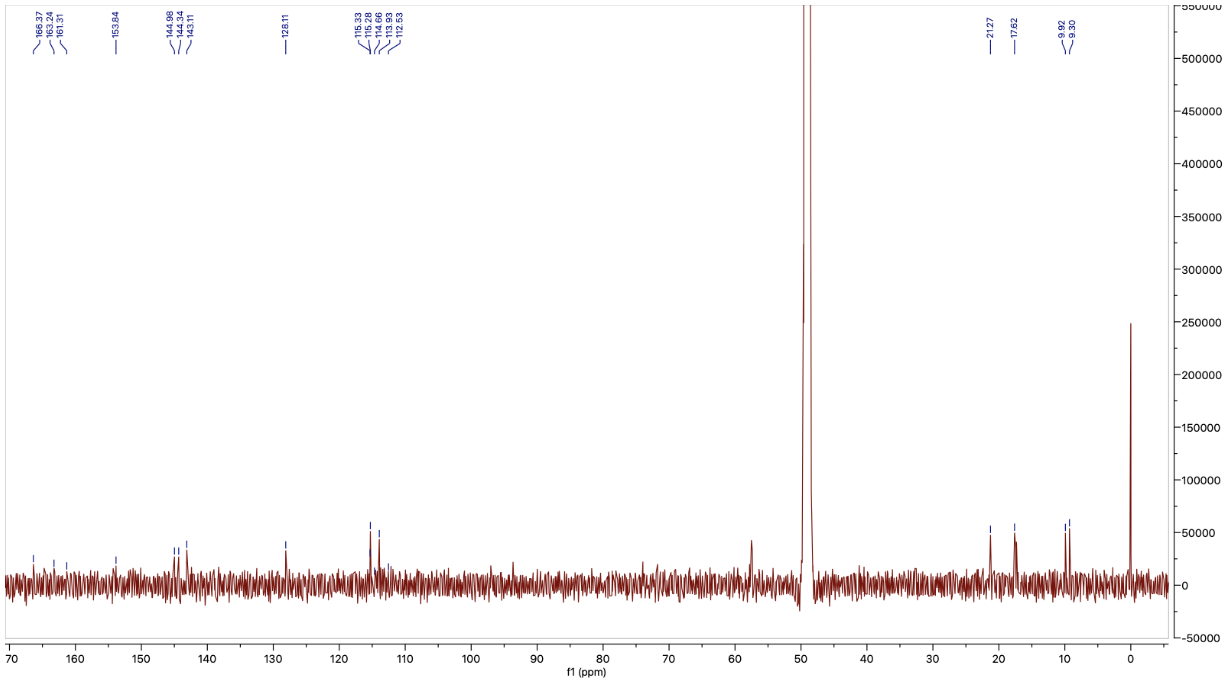

Figure S29. The ^{13}C NMR spectrum of 8 in CD_3OD (150 MHz)

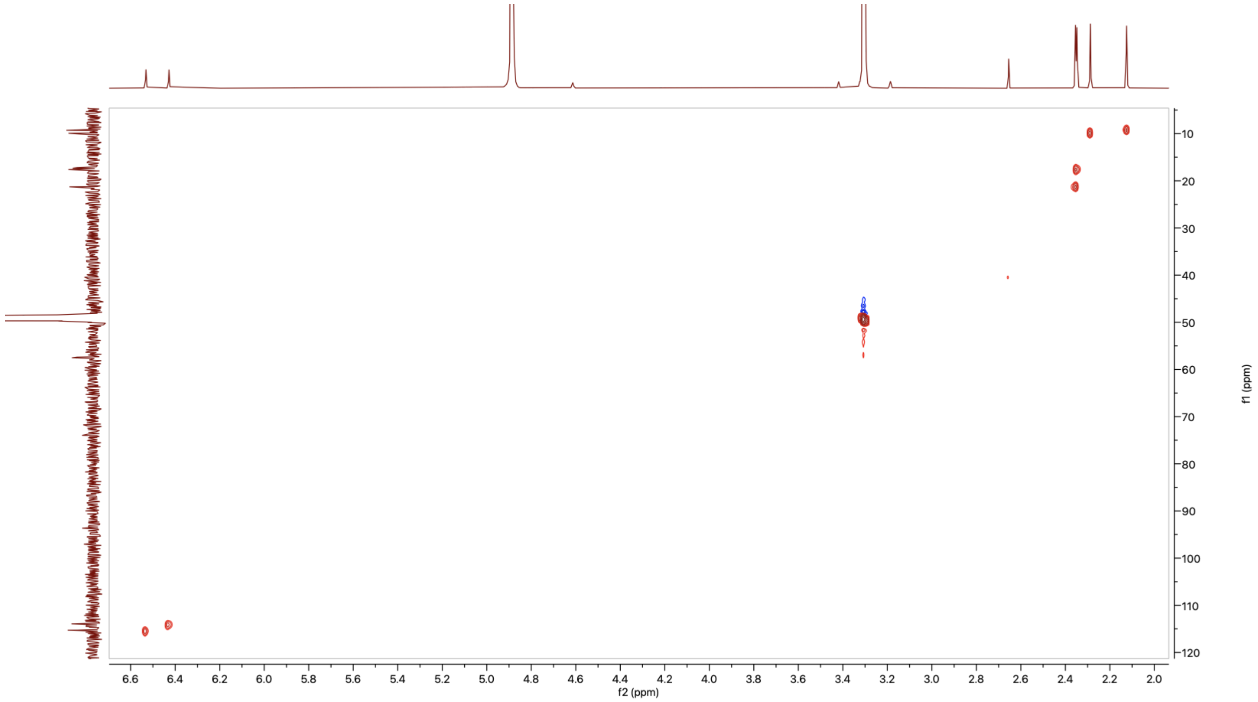

Figure S30. The ^{1}H-^{13}C HSQC NMR spectrum of 8 in CD_3OD (600 MHz)

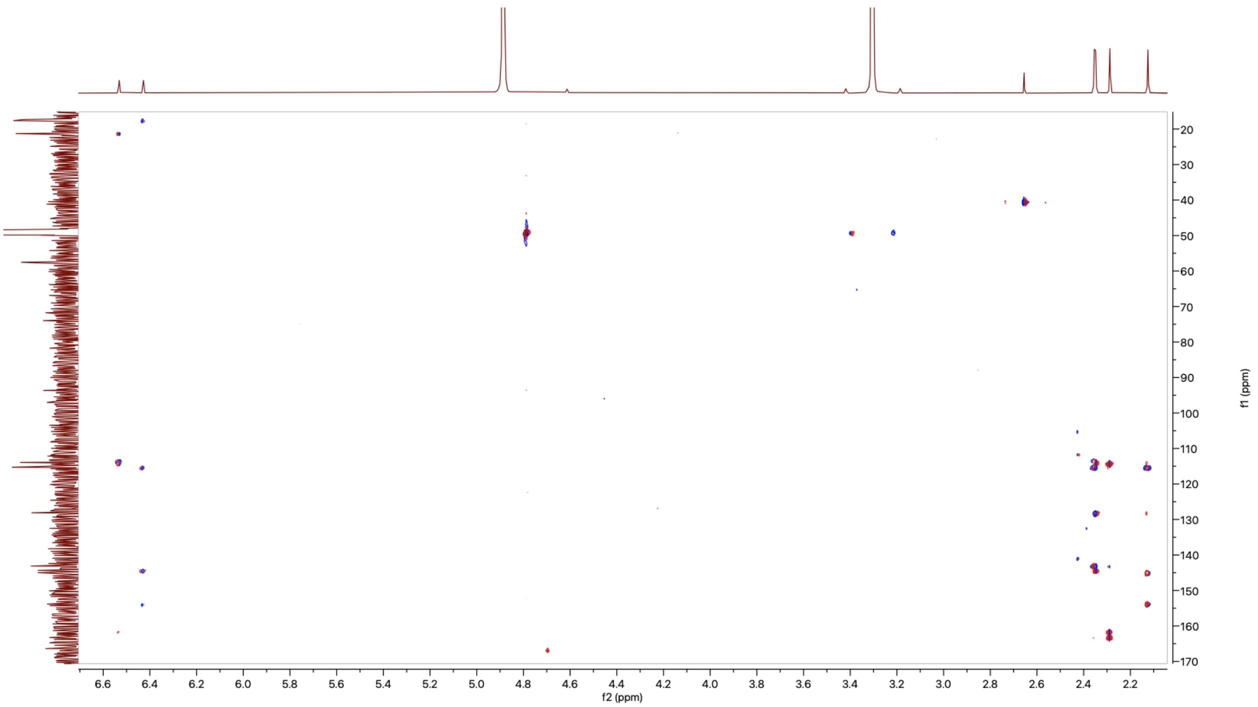

Figure S31. The ^{1}H-^{13}C HMBC NMR spectrum of 8 in CD_3OD (600 MHz)

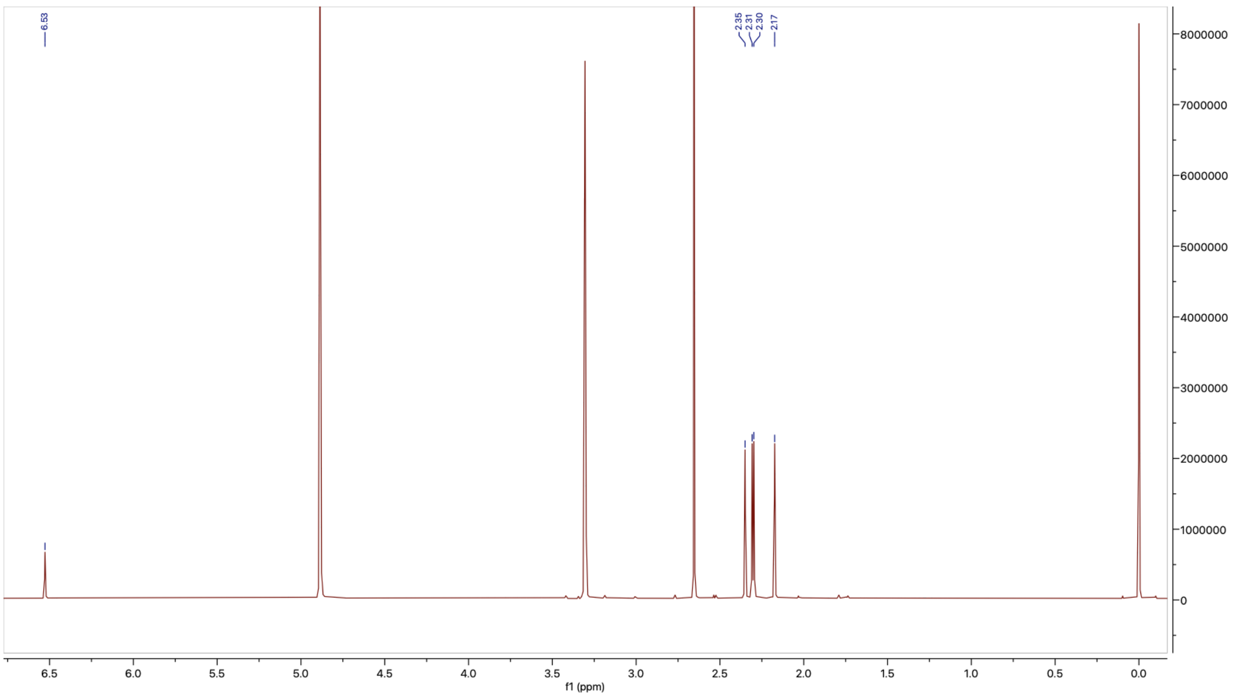

Figure S32. The ^{1}H NMR spectrum of 10 in CD_3OD (600 MHz)

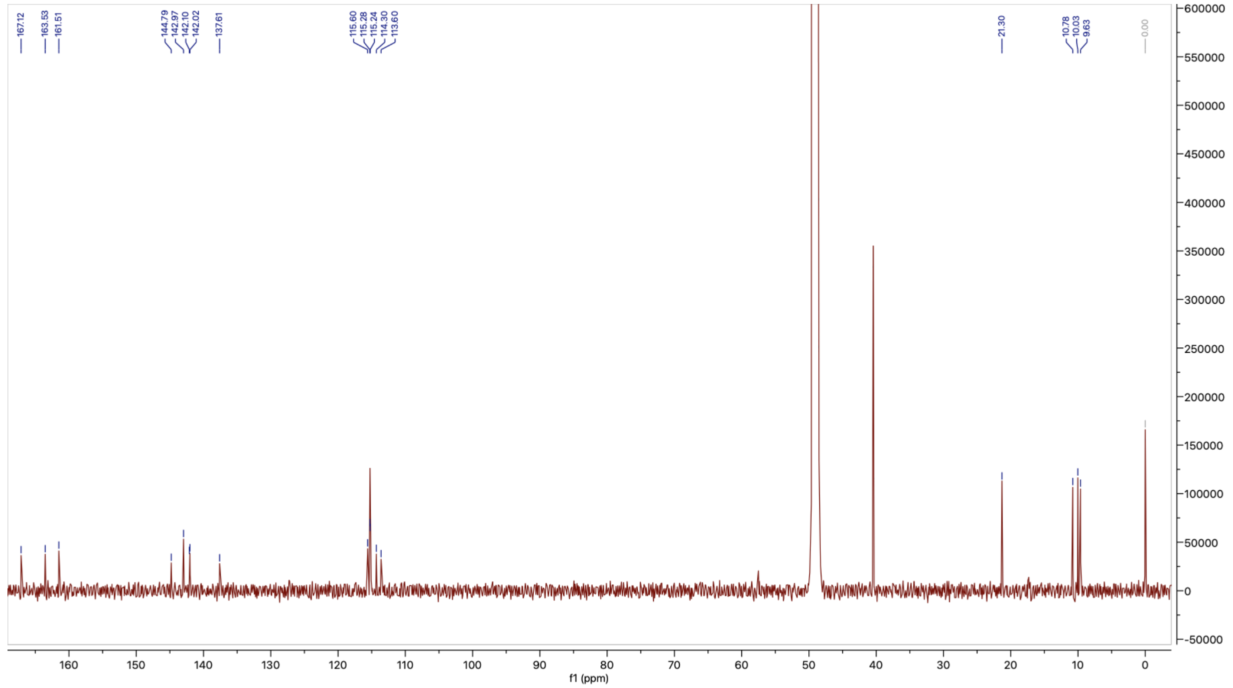

Figure S33. The ^{13}C NMR spectrum of 10 in CD_3OD (150 MHz)

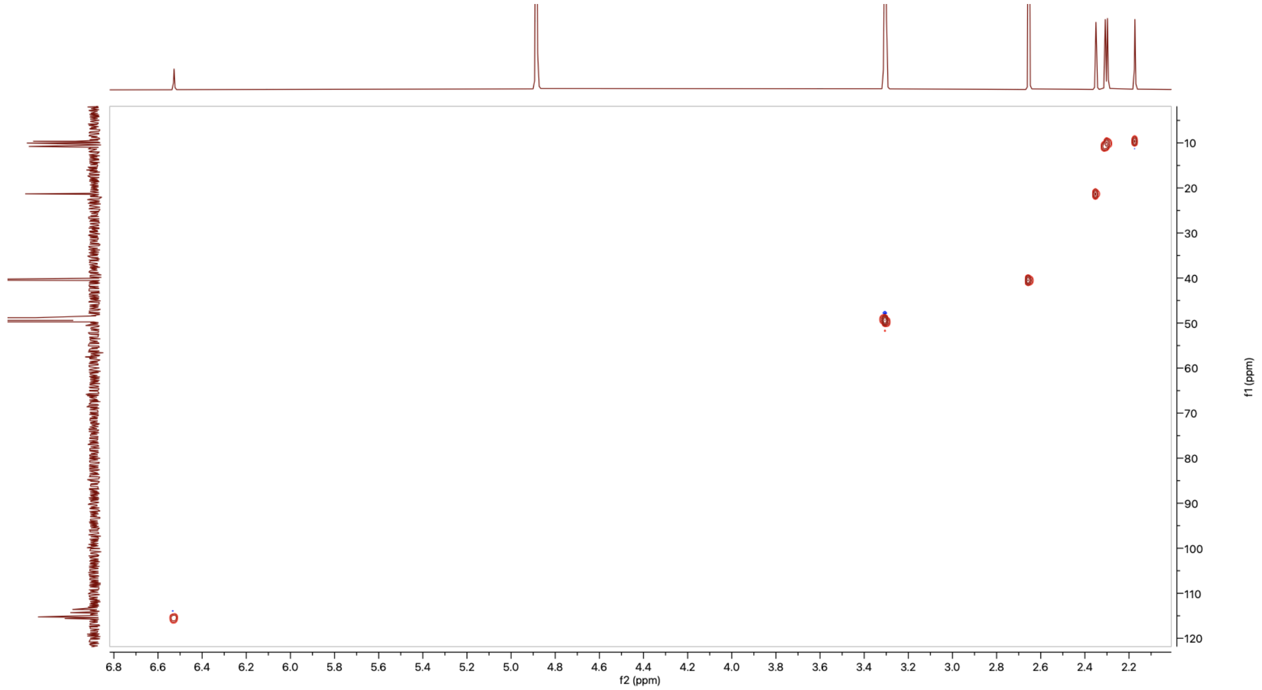

Figure S34. The ^{1}H-^{13}C HSQC NMR spectrum of 10 in CD_3OD (600 MHz)

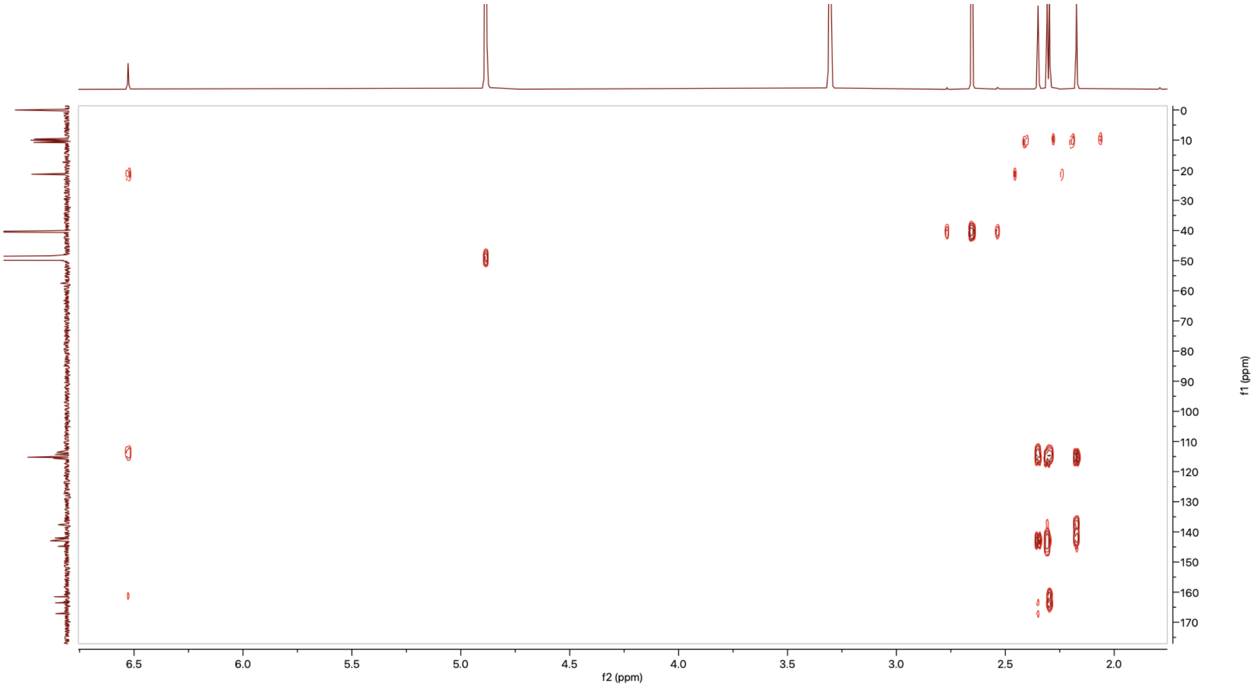

Figure S35. The ^{1}H-^{13}C HMBC NMR spectrum of 10 in CD_3OD (600 MHz)

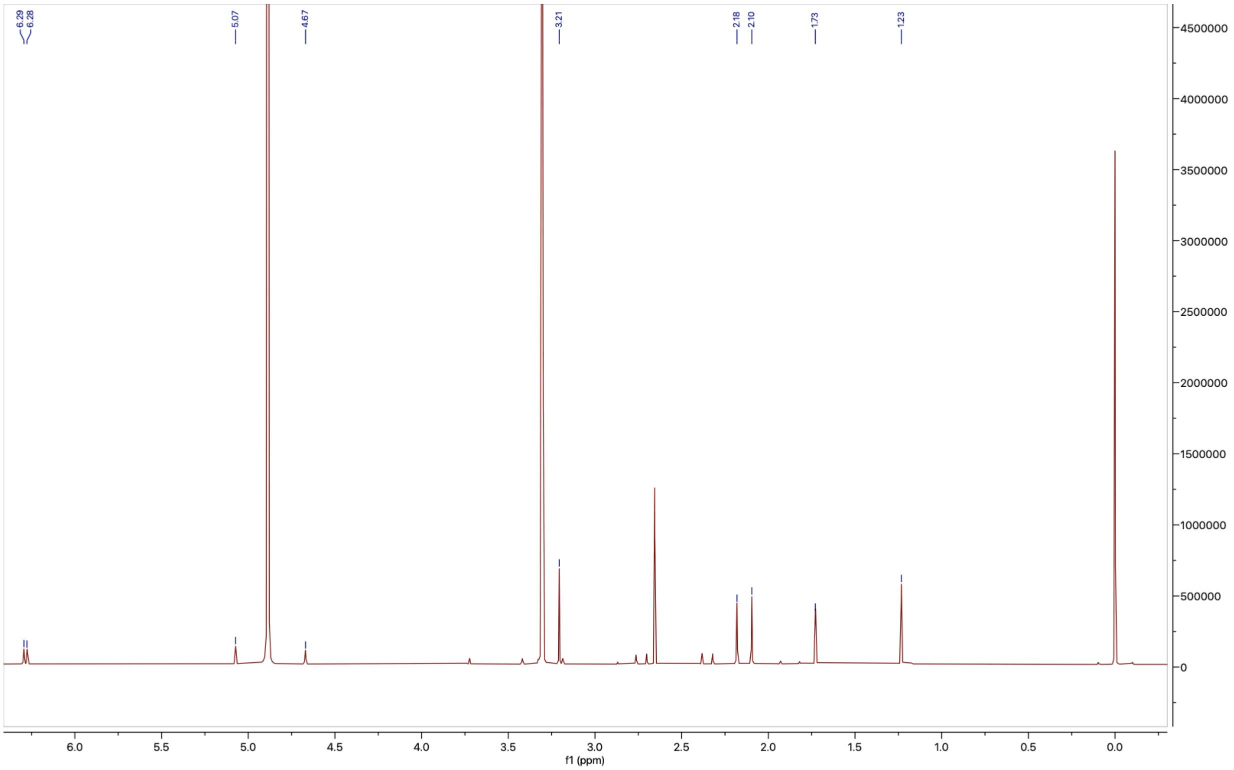

Figure S36. The ^{1}H NMR spectrum of 11 in CD_3OD (600 MHz)

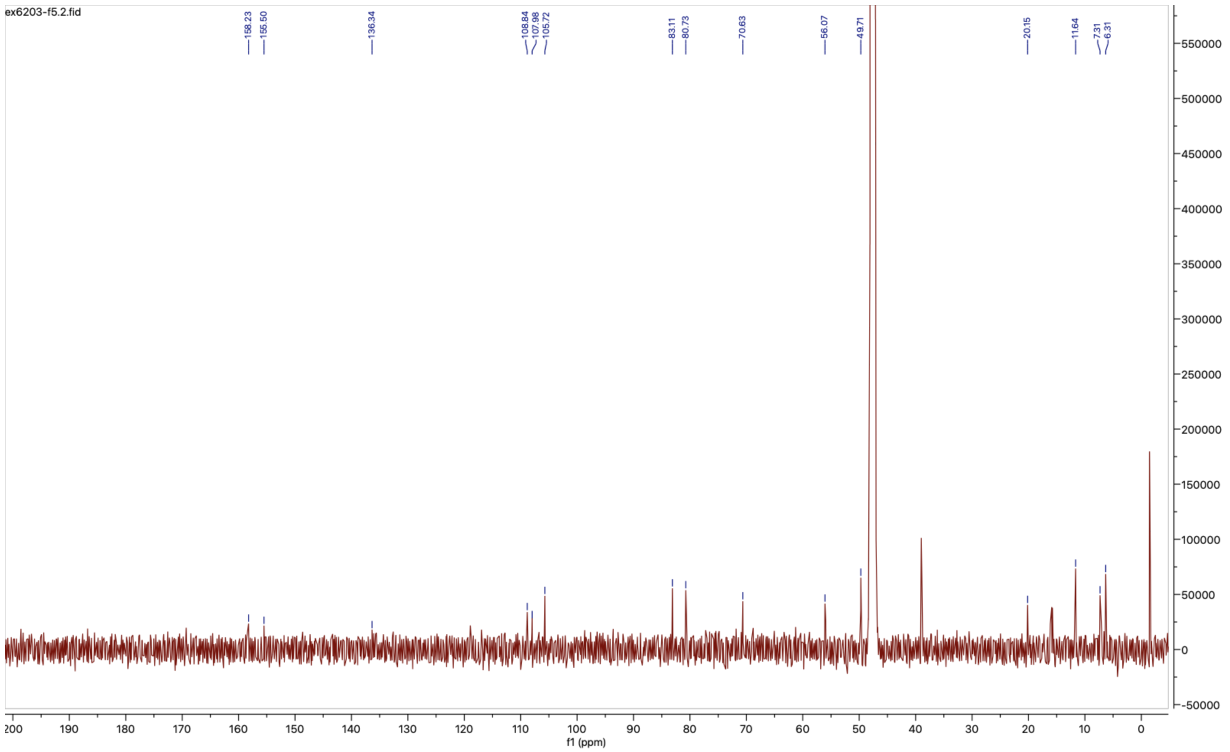

Figure S37. The ^{13}C NMR spectrum of 11 in CD_3OD (150 MHz)

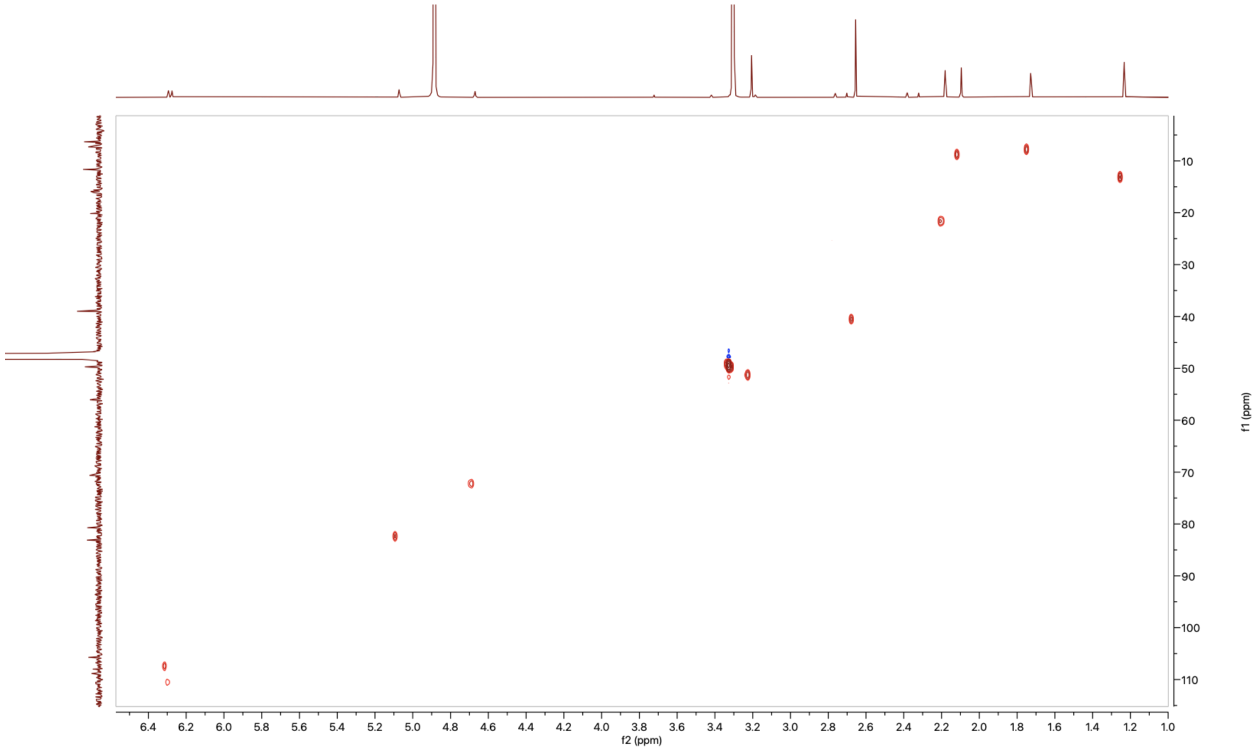

Figure S38. The ^{1}H-^{13}C HSQC NMR spectrum of 11 in CD_3OD (600 MHz)

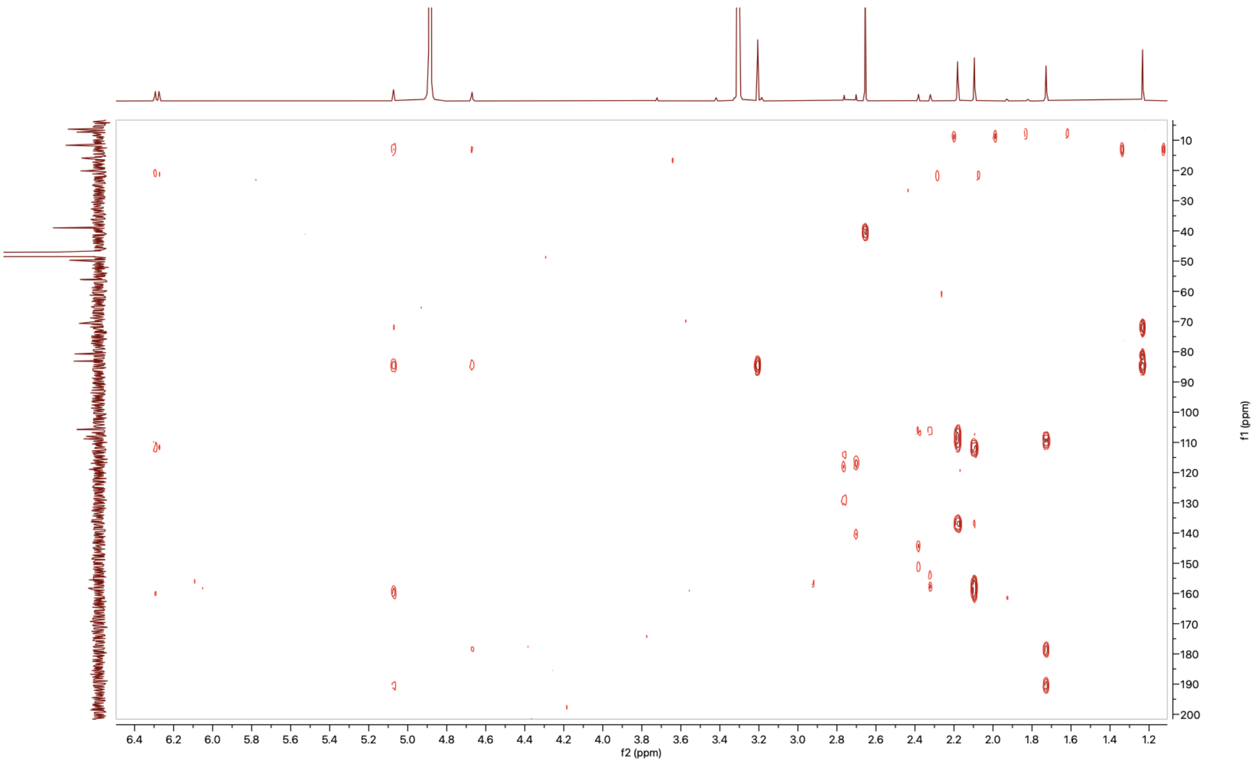

Figure S39. The ^{1}H-^{13}C HMBC NMR spectrum of 11 in CD_3OD (600 MHz)

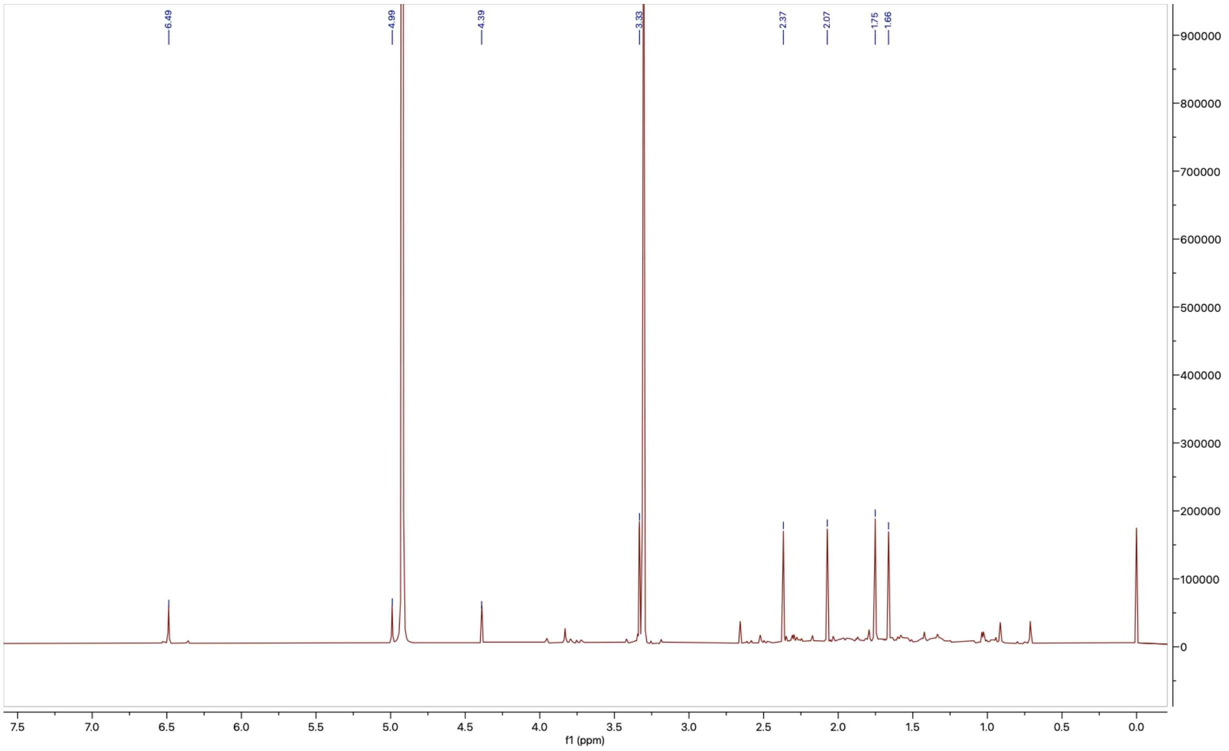

Figure S40. The ^{1}H NMR spectrum of 12 in CD_3OD (600 MHz)

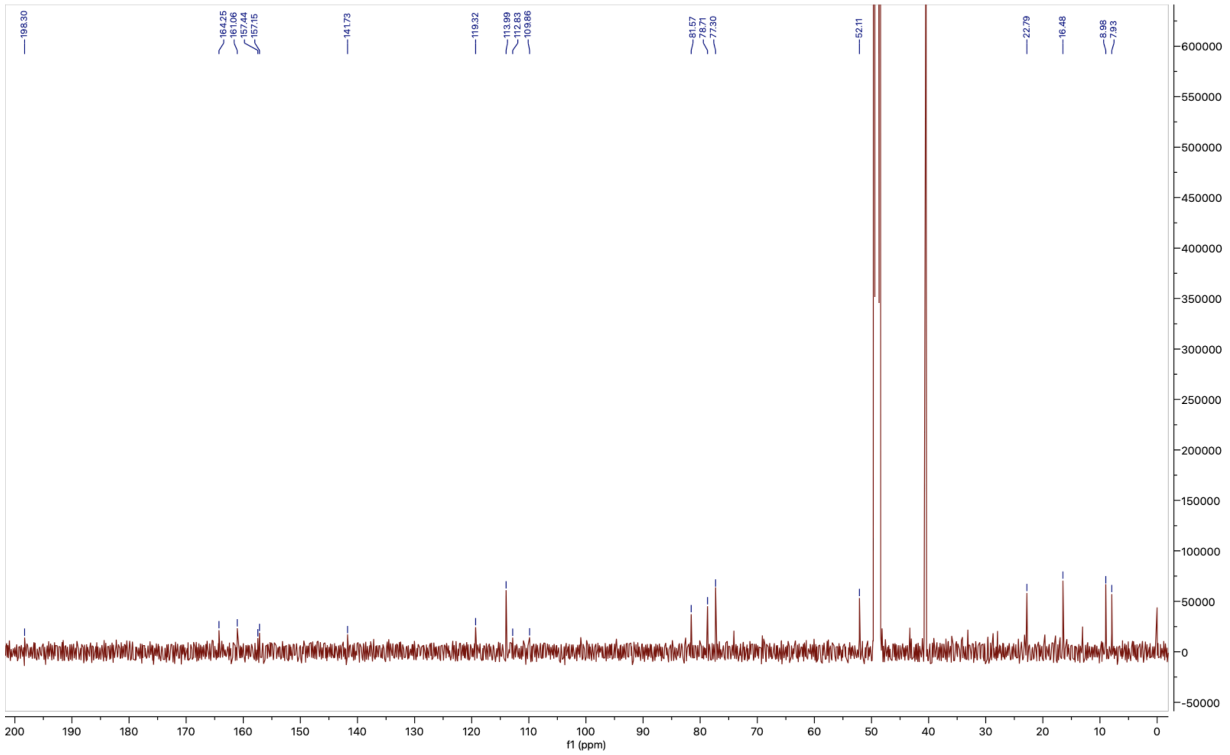

Figure S41. The ^{13}C NMR spectrum of 12 in CD_3OD (150 MHz)

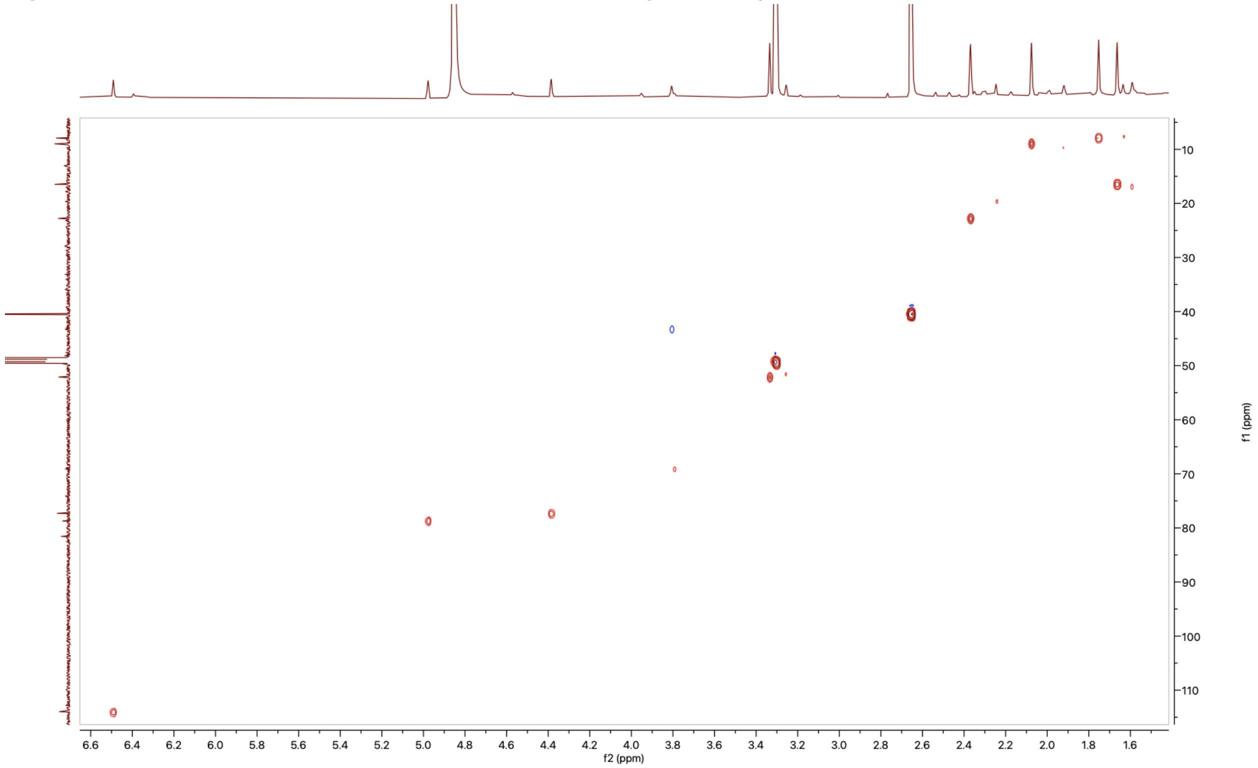

Figure S42 The ^{1}H-^{13}C HSQC NMR spectrum of 12 in CD_3OD (600 MHz)

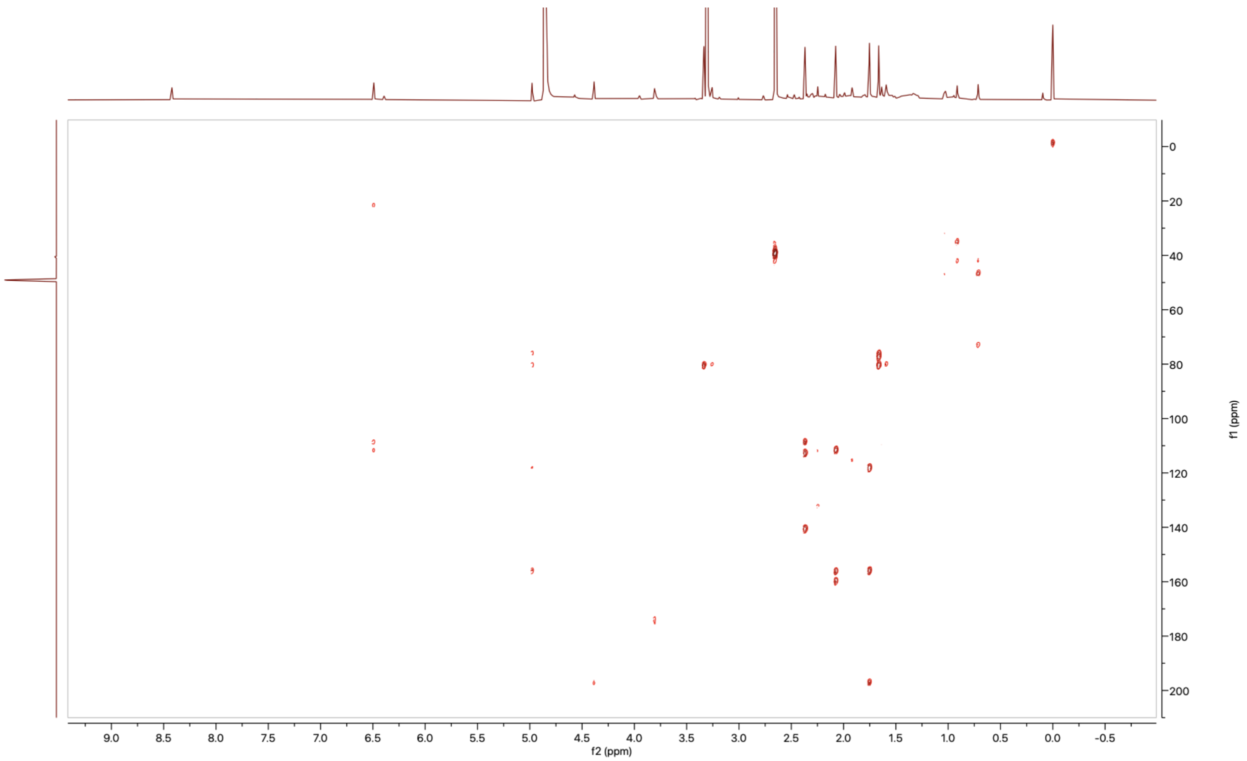

Figure S43. The ^{1}H-^{13}C HMBC NMR spectrum of 12 in CD_3OD (600 MHz)

References

1. Shang, Z. *et al.* Roseopurpurins: Chemical Diversity Enhanced by Convergent Biosynthesis and Forward and Reverse Michael Additions. *Org Lett* **18**, 4340–4343 (2016).
2. Kim, W. et al. Linking a Gene Cluster to Atranorin, a Major Cortical Substance of Lichens, through Genetic Dereplication and Heterologous Expression. *Mbio* **12**, e01111-21 (2021).
3. Sala, T. & Sargent, M. V. Depsidone synthesis. Part 19. Some β-orcinol depsidones. *J Chem Soc Perkin Transactions 1* **0**, 877–882 (1981).
